# New Quinoline Kinase Inhibitors With Good Selectivity for NAK Kinases and Anti‐Tumor Activity Against Ewing Sarcoma

**DOI:** 10.1002/ardp.70184

**Published:** 2026-01-10

**Authors:** Caroline de Bem Gentz, Thais Helena Maciel Fernandes, Marcela Silva Lopes, Lewis Elson, Andreas Krämer, Lucas Rodrigo de Souza, Isadora Serraglio Fortes, Geórgia Silva Pinto, Martha Cestari Silva Martins, Henrique Barros de Lima, André da Silva Santiago, Lauro José Gregianin, Katlin Brauer Massirer, Mário Henrique Bengtson, Rafael Roesler, Stefan Knapp, Stefan A. Laufer, Saulo Fernandes de Andrade

**Affiliations:** ^1^ Pharmaceutical Sciences Graduate Program Universidade Federal do Rio Grande do Sul (UFRGS) Porto Alegre Rio Grande do Sul Brazil; ^2^ Pharmaceutical Synthesis Group (PHARSG), School of Pharmacy Universidade Federal do Rio Grande do Sul (UFRGS) Porto Alegre Rio Grande do Sul Brazil; ^3^ Department of Pharmacology, Institute of Basic Health Sciences Universidade Federal do Rio Grande do Sul (UFRGS) Porto Alegre Rio Grande do Sul Brazil; ^4^ National Science and Technology Institute for Children′s Cancer Biology and Pediatric Oncology ‐ INCT BioOncoPed Porto Alegre Brazil; ^5^ Institute of Pharmaceutical Chemistry Goethe University Frankfurt am Main Germany; ^6^ Center for Medicinal Chemistry (CQMED), Center for Molecular Biology and Genetic Engineering (CBMEG) University of Campinas (UNICAMP) Campinas São Paulo Brazil; ^7^ Cancer and Neurobiology Laboratory, Experimental Research Center, Clinical Hospital (CPE‐HCPA) Universidade Federal do Rio Grande do Sul Porto Alegre Rio Grande do Sul Brazil; ^8^ Department of Pediatrics, School of Medicine Universidade Federal do Rio Grande do Sul Porto Alegre Rio Grande do Sul Brazil; ^9^ Pediatric Oncology Service, Clinical Hospital Universidade Federal do Rio Grande do Sul Porto Alegre Rio Grande do Sul Brazil; ^10^ Department of Pharmaceutical and Medicinal Chemistry, Institute of Pharmaceutical Sciences University of Tübingen Tübingen Germany

**Keywords:** Ewing Sarcoma, GAK, inhibitor, panel of kinases, quinoline

## Abstract

In the past few years, several novel anticancer agents targeting protein kinases have been discovered expanding the available therapeutic arsenal. However, few new therapeutic approaches have been developed for the treatment of childhood cancer. To this end, we have been making efforts to contribute to this important field. Herein, we identified a series of new 4,6‐disubstituted quinoline derivatives from our in‐house quinoline chemical library that showed promising anti‐proliferative activity against Ewing Sarcoma (ES). This interesting observation engaged us to further investigate these derivatives since this type of cancer is among the most common bone cancers in children. Evaluation of the quinoline derivatives against a panel of kinases demonstrated generally narrow selectivity profiles of this compound class. Interestingly, the main kinases that were inhibited belonged to the NAK family of kinases, in particular, the family member cyclin G‐associated kinase (GAK) which was inhibited at nanomolar range in enzyme kinetic assays.

## Graphical abstract

Focusing on the discovery of new therapeutics for childhood cancer, novel quinoline derivatives were synthesized and demonstrated activity against Ewing sarcoma. A target search was performed against a panel of kinases, and the compounds showed selectivity for the NAK family, mainly GAK, which was confirmed by enzyme kinetic assays.

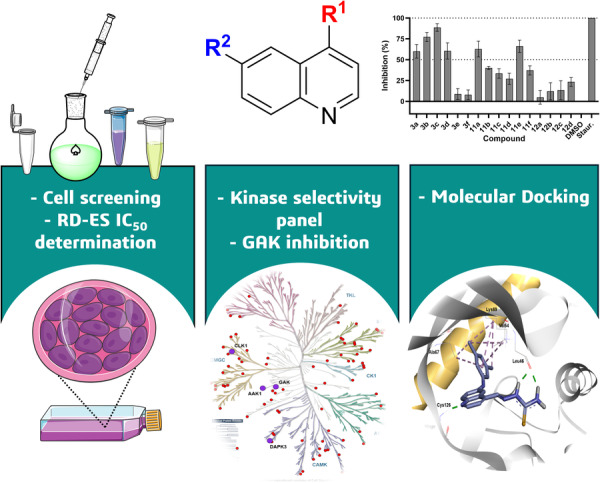

## 1 Introduction

Cancer is a global public health challenge and includes more than 100 different types of diseases. These diseases are characterized by the development of abnormal cells with deregulated growth rate, lack of differentiation, and dysregulation of apoptosis factors that allow cancer to proliferate. Despite the high cure rate for childhood cancer [1], pediatric cancer continues to be the major cause of death among children and young adults between 1 and 19 years. Cancers in children frequently contrast with those in adults regarding the affected organs, as well as their tissue and biological origins. While the majority of adult cancer—such as lungs, breast, and colorectal malignancies—are classified as carcinomas arising from epithelial tissue, mesodermal and embryonal types are more common during childhood. This distinction is evident in the prevalence of leukemias, lymphomas, and sarcomas among younger patients [2].

Despite major advances in adult cancer therapies, progress in pediatric oncology has lagged. In the European Union, the USA, and Japan, a total of 103 molecular targeted drugs, including kinase inhibitors, were approved for adult patients in at least one of these regions, in contrast with only 19 drugs approved for pediatric patients as of February 2020. Among antineoplastic drugs with pediatric indications are the kinase inhibitors Dasatinib, Everolimus, Imatinib, and Nilotinib [3]. On average, pediatric trials begin 6.5 years after adult testing, with delays reaching up to 28 years [2, 4]. Furthermore, classical treatments, such as old non‐specific drugs and radiotherapy that are commonly used to treat pediatric malignancies, carry with them the potential for long‐term consequences, including damage to deoxyribonucleic acid (DNA) and an elevated risk of future malignancies [5]. Therefore, the identification of new promising pediatric anticancer compound especially those targeting the particularities of these diseases is urgent [6].

The National Science and Technology Institute for Children′s Cancer Biology and Pediatric Oncology (INCT BioOncoPed) aims to study therapeutic strategies for major pediatric cancers such as Ewing Sarcoma (ES) and Medulloblastoma. ES is a highly aggressive malignant tumor that develops in bone and soft tissue [7, 8]. It primarily affects children and adolescents, with most cases arising between the ages of 10 and 19. Current treatment relies on a multimodal approach, combining surgical resection, intensive multiagent chemotherapy, and local radiotherapy. While this strategy has improved the 5‐year survival rate of localized ES to over 70%, outcomes for metastatic and recurrent cases remain poor, with survival rates around 30% [9, 10]. Medulloblastoma is the most frequently diagnosed malignant brain tumor in children. Although current treatments can be effective, they are highly toxic, often leaving survivors with lasting neurological impairments [11, 12]. This highlights the urgent need to explore alternative therapeutic strategies for pediatric diseases.

**Figure 1:**
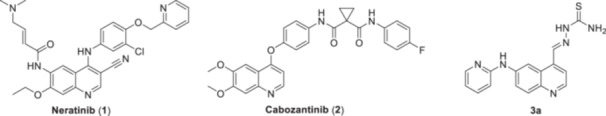
Kinase inhibitors presenting a quinoline core (Neratinib and Cabozantinib) and the novel compound **3a**.

Over the past few years, our group has focused on identifying anticancer and antimicrobial compounds and enzyme inhibitors derived from heterocyclic compounds, particularly those featuring the quinoline core [13, 14, 15, 16, 17, 18, 19]. As a result of several studies carried out with this class, we and other groups have confirmed that it is indeed a privileged structure and the appropriate selection of substituents can lead to the desired selective inhibitory and/or pharmacological activities [13, 14, 15, 16, 17, 18, 19, 20, 21, 22, 23]. Furthermore, these efforts have led to the creation of a diverse chemical library comprising over 300 derivatives, which have been systematically used to identify new bioactive compounds. The development of antitumor drugs from quinoline derivatives is indeed a significant strategy in the 21st century, having already led to the discovery of several modern drugs, including FDA‐approved kinase inhibitors such as Neratinib (**1**) and Cabozantinib (**2**) [24] (Figure 1).

Recently, an important perspective paper published in the Journal of Medicinal Chemistry with robust discussed data highlighted the cell‐based approach as an important strategy for drug discovery. An example of this approach was the discovery of paclitaxel in which 9KB cells were used to evaluate the potential in vitro cytotoxicity of samples obtained from *Taxus brevifolia* in the early steps [25]. Using our quinoline chemical library and in line with this strategy, we employed viability assays to screen our quinoline derivatives in cell lines of interest. This screening led to the identification of compound **3a** (Figure 1), which presented an IC_50_ value of 6.7 µM against the RD‐ES cell line. We chose this compound as the starting point for our study because of its promising biological activity against the RD‐ES cell line and the originality of its substituents. In addition, the well‐documented kinase‐inhibitory potential of quinoline derivatives combined with the urgent need for novel drug candidates for the treatment of ES further supports the optimization of compound **3a**.

## 2 Results and Discussion

### 2.1 Chemistry

**Scheme 1:**
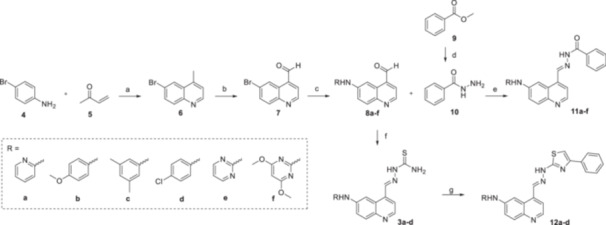
Synthesis of derivatives **3a–d**, **11a–f** and **12a–d**. Reagents and conditions: (a) H_2_SO_4_, dioxane, reflux, 24 h; (b) SeO_2_, H_2_O, dioxane, reflux, 4 h; (c) Pd(OAc)_2_, xantphos, appropriate aniline, Cs_2_CO_3_, dioxane, refllux, 16 h; (d) hydrazine hydrate 16%, EtOH, reflux, 3 h; (e) AcOH, EtOH, 25°C, 4 h; (f) thiosemicarbazide, AcOH, EtOH, reflux, 2‐3 h; (g) 2‐bromoacetophenone, isopropanol, reflux, 1–2 h.

**Scheme 2:**
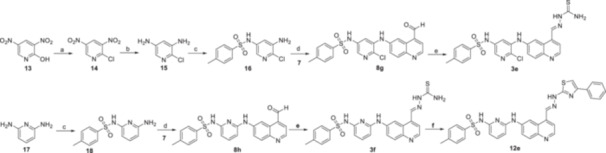
Synthesis of sulfonamide derivatives **3e–f** and **12e**. Reagents and conditions: (a) POCl_3_, DMF, 105°C, 16 h; (b) SnCl_2_, EtOH, reflux, 2 h; (c) *p*‐toluenesulfonyl chloride, pyridine, EtOH, reflux, 18 h; (d) Pd(OAc)_2_, xantphos, appropriate aniline, Cs_2_CO_3_, dioxane, reflux, 16 h; (e) thiosemicarbazide, AcOH, EtOH, reflux, 2‐3 h; (f) 2‐bromoacetophenone, isopropanol, reflux, 1–2 h.

The derivatives of compound **3a** were synthesized in four or five steps as shown in Scheme 1, using 4‐bromoaniline (**4**) and 3‐buten‐2‐one (**5**) as starting materials for the quinoline ring formation via a modified Skraup synthesis. In the second step, the methyl group of 6‐bromo‐4‐methylquinoline **6** was oxidized with selenium dioxide, resulting in aldehyde **7** [26]. Next, a palladium‐catalyzed cross‐coupling reaction with the appropriate amine was performed, affording compounds **8a–f** that were immediately used to prepare series **3** and/or **11** [13]. For the thiosemicarbazone **3a**–**d** formation, **8a–d** were treated with thiosemicarbazide. Regarding hydrazone series **11**, first benzhydrazide **10** was prepared from methyl benzoate **9** by treatment with hydrazine hydrate, then, **10** was reacted with **8a**–**f** [27, 28, 29]. We also described here the preparation of the sulfonamide aminopyridines **16** and **18** that were used to synthesize the thiosemicarbazones **3e**–**f** as shown in Scheme 2. The sulfonamide aminopyridine **18** was prepared directly from the commercially available diamine **17** and tosyl chloride. On the other hand, it was necessary to synthesize diamine **15** from 2‐hydroxy‐3,5‐nitropyridine **13** in two steps. In the first step, the 2‐hydroxyl group was converted into the halide **14** using POCl_3_, and then dinitro groups were reduced using SnCl_2_. Finally, compounds **3a**–**d** and **3f** were cyclized with 2‐bromoacetophenone to afford derivatives **12a–e** [30].

### 2.2 Cellular Screening and IC_50_ Determination

We chose to investigate the activity of our inhibitor set against two INCT prioritized pediatric cancer types such as medulloblastoma, the most common malignant childhood brain tumor, and ES, an aggressive pediatric tumor with poor prognosis [31, 32]. We selected the desmoplastic cerebellar medulloblastoma cell line Daoy, and the epithelial ES cell line RD‐ES for evaluation of the cytotoxic activity of the compounds by MTT viability assays at a fixed compound concentration of 10 µM (Table 1).

**Table 1:** Percentage of cell viability affected at 10 µM using the cell lines RD‐ES and Daoy.

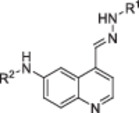				
Entry	R^1^	R^2^	% viability 10 µM (RD‐ES)^a^	% viability 10 µM (Daoy)^a^
**3b**	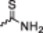	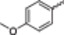	51.5 ± 0.0%	96.3 ± 0.0%
**3c**	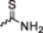	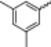	25.7 ± 0.1%	44.6 ± 0.0%
**3d**	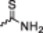	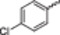	63.1 ± 0.1%	85.5 ± 0.0%
**3e**	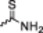	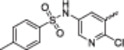	93.8 ± 0.1%	95.0 ± 0.0%
**3f**	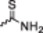	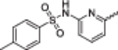	59.4 ± 0.0%	92.4 ± 0.0%
**11a**	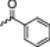	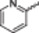	47.2 ± 0.1%	82.3 ± 0.1%
**11b**	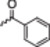	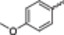	42.1 ± 0.2%	45.4 ± 0.1%
**11c**	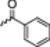	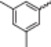	61.6 ± 0.1%	70.0 ± 0.2%
**11d**	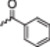	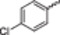	50.4 ± 0.1%	89.1 ± 0.0%
**11e**	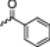	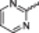	43.4 ± 0.0%	65.9 ± 0.0%
**11f**	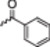	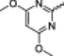	42.3 ± 0.0%	77.5 ± 0.0%
**12a**	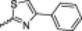	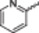	74.9 ± 0.0%	62.1 ± 0.1%
**12b**	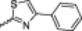	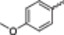	78.9 ± 0.2%	81.7 ± 0.1%
**12c**	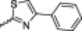	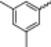	66.8 ± 0.1%	85.0 ± 0.0%
**12d**	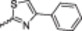	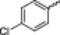	50.4 ± 0.2%	86.0 ± 0.0%
**12e**	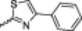	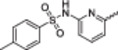	70.3 ± 0.1%	100 ± 0.0%
**Vincristine**	—	—	40.55 ± 0.0%	—
**AZD5363**	—	—	—	49.65 ± 2.6%

^a^ Data represent the mean ± SD of at least two independent experiments, each performed in technical triplicate.

In general, compounds were more potent affecting the viability of RD‐ES compared with Daoy; for this reason, we concentrate on RD‐ES cells. Overall, the thiazole series (**12**) was inactive against RD‐ES, whereas the hydrazones (**11**) and thiosemicarbazones (**3**) provided active derivatives. The collected data indicate that substituent variation at the 6‐position of the quinoline ring does not significantly affect the activity of hydrazone derivatives (42%–62% viability at 10 µM) but has a great impact on thiosemicarbazone series (25%–94% viability at 10 µM). Thus, the potency of thiosemicarbazone derivatives ranged from slightly to very active depending on the 6‐substituent, as we can observe for the dimethylphenyl substituent in **3c** (25.7%) that could increase the activity at least twofold in comparison with other substituents. The heterogeneity among the thiosemicarbazone activities could be explained by the smaller size of this group, compared with the hydrazone, allowing greater variability in how the compounds interact with the biological target.

Among the active compounds of both series, the focus was to determine the IC_50_ of the compounds that exhibited more than 50% RD‐ES toxicity (Table 2).

**Table 2:** IC_50_ values (viability) of the most active compounds against RD‐ES cell line.

Compound	IC_50_ RD‐ES (µM)^a^
**3a**	6.8 ± 0.3
**3c**	1.1 ± 0.0
**11a**	5.0 ± 0.6
**11b**	3.6 ± 0.7
**11d**	5.1 ± 0.2
**11e**	6.4 ± 1.8
**11f**	6.1 ± 0.5
**12d**	> 10
**Vincristine**	0.005 ± 0.0006

^a^ Data represent the mean ± SD of three independent experiments, each performed in technical triplicate. Compounds were evaluated at six different concentrations (50.0–2.5 µM or 7.2–0.225 µM). Vincristine was used as a positive control (0.3–0.0047 µM).

Most of the evaluated compounds presented an IC_50_ < 10 µM. It is noteworthy that, for series **11**, almost all tested substituents resulted in compounds with significant activity, excluding the dimethylphenyl. By contrast, this same substituent provided the most active derivative of series **3** (**3c**). Except for **11b**, which demonstrated comparable activity against both tumor cell lines, the selectivity for RD‐ES cell line associated with marked cytotoxicity revealed an interesting profile of the derivatives. These results encouraged us to further investigate their effects and to elucidate the underlying mechanism of action of these compounds.

### 2.3 Screening Against a Kinase Panel Revealed Narrow Selectivity

**Figure 2:**
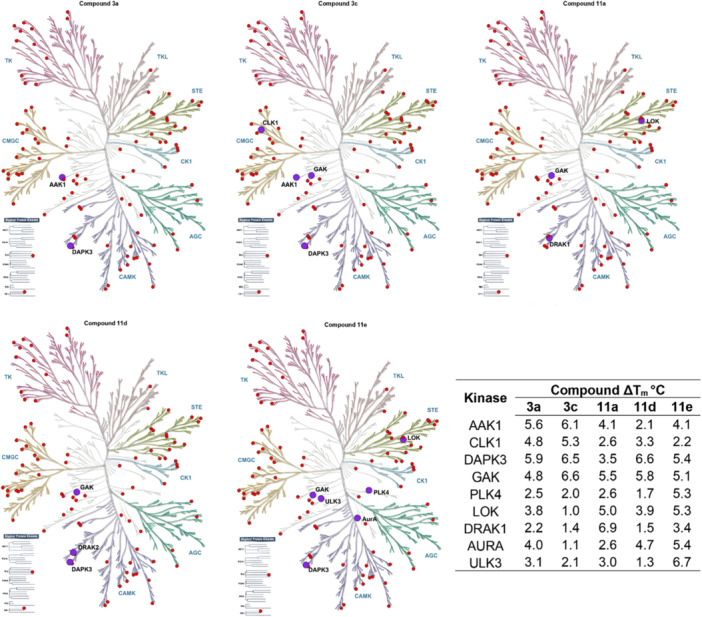
Selectivity evaluation. The protein kinases used for the thermal shift assay are highlighted as red dots in the dendrogram of the human kinome. Potential targets (Δ*T*_m_ > 5°C) are marked as purple dots. The graphic was generated with KinMap [34].

**Figure 3:**
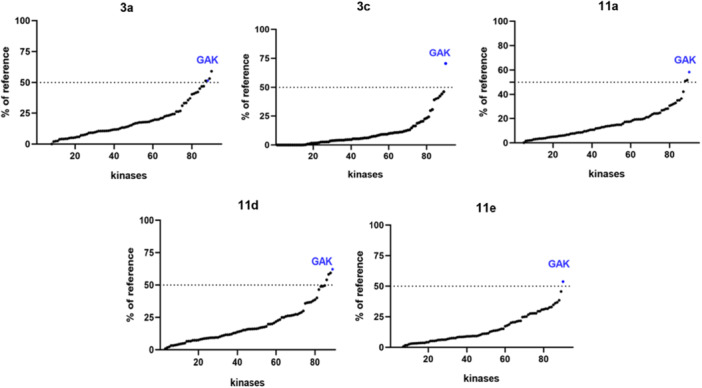
Selectivity evaluation. Waterfall plots of compounds against a panel of kinases. GAK is highlighted as a blue point.

Given the strong potential of quinoline compounds for kinase inhibition, we started the target search by evaluating their interactions with a kinase panel. A thermal shift screen was conducted to evaluate the selectivity of the compounds against a panel of recombinant kinases [33]. This assay detects protein−ligand interactions by measuring shifts in protein thermal stability. Inhibitor binding increases the melting temperature (*T*_m_), whereas a higher *T*_m_ shift usually correlates with higher affinity. However, thermal shifts may differ between kinases, and we therefore included Staurosporine as a general positive control. Considering the previous study, we selected a Δ*T*_m_ > 5°C as a cut‐off for significant hits. In this work, 95 kinases were examined (Figures 2 and 3). It is important to mention that this selectivity panel did not cover the kinome, and activity on other kinases can not be excluded. In addition, the panel contained also typical off‐targets of kinase inhibitors outside the kinase family including bromodomains and a few other proteins [35].

Overall, the screened compound set showed significant temperature shifts for kinases belonging to the NAK (Numb‐associated) kinase family. The kinases belonging to this family are AAK1 (adaptor‐associated kinase 1), BIKE (BMP‐2‐inducible kinase), GAK (cyclin G‐associated kinase), and MPSK1 (palmitoylated serine/threonine kinase 1), also known as STK16, which was not included in our panel. These kinases are known to mediate vesicular transport in clathrin‐coated vesicles and have been widely studied as targets for developing antiviral treatment strategies. However, their potential as targets in oncology are less known [36, 37, 38].

Interestingly, in a knockdown study in which 673 potential targets were knocked down in a bone cancer cell line, only nine kinases were relevant for cell growth, including GAK [39]. Besides, bone cancer is a major clinical challenge due to its aggressive nature and resistance to treatment, and GAK has gained attention due to its possible correlation with poor patient prognosis [39]. In our study, this enzyme was widely inhibited by our inhibitor series. Furthermore, the two cell lines that we used in the experiments differ in GAK RNA expression (RD‐ES—59 nTPM; Daoy—30.1 nTPM) as indicated in Human Protein Atlas [40, 41]. Clearly, compounds were more active against RD‐ES, which could suggest favorable selectivity of the compounds for this cell line.

The results of the panel assay also indicated that, overall, only a few kinases interacted with the compounds. In particular, compounds **3a**, **3c**, **11a**, and **11d** have narrow selectivity profiles with only two to four kinases of the 95 evaluated kinases showing significant Δ*T*_m_ shifts. The major off‐target of the compounds was DAPK3 that was involved in interaction with 4 (**3a**, **3c**, **11d**, and **11e**) of the evaluated compounds. Interestingly, compound **11a** (4‐*N*‐acylhydrazone) did not interact with DAPK3, while **3a** (4‐thiosemicarbazone) showed a significant *T*_m_ shift. This result suggests that the substituent at the 4‐position of the quinoline could modulate the compound's off‐targets. Besides, **11a** also targeted DRAK1 and LOK that are relevant kinases for drug discovery. These kinases are being used as targets in the development of new anticancer compounds against glioblastoma multiforme [42], uveal melanoma [43], and ES [44].

### 2.4 GAK Inhibitors Were Active in Enzyme Kinetic Assays

**Figure 4:**
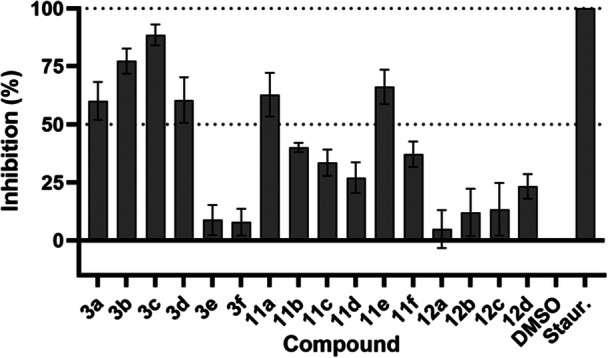
GAK inhibition assessment by the synthesized compounds at 1 µM. Staurosporine and DMSO were set as positive (100% inhibition) and negative (0% inhibition) controls for normalization, respectively. Data represent the mean ± SEM of three independent experiments, with at least two technical replicates each.

Encouraged by the selectivity data and the potential link of GAK with sarcoma cell proliferation, we proceeded with enzyme kinetic assays to show that our compounds would also inhibit GAK kinase activity. The initial inhibition screening was performed at 1 µM to find the most promising derivatives concerning GAK inhibition. The results are shown in Figure 4.

Compounds that inhibited ≥ 50% of GAK activity were selected to determine the IC_50_ and *K*_i_ values. As expected, based on the screening, the derivatives **3a**, **3b**, **3c**, **3d**, **11a**, and **11e** were active in the nanomolar range, indicating high potency against GAK (Table 3). All the derivatives that were effective to inhibit GAK belong to hydrazone or thiosemicarbazone series, while the thiazole series was inactive against the enzyme. In general, these data were in agreement with the cellular activity in Ewing′s Sarcorma cell line. Interestingly, the thiosemicarbazone **3c** was the most active derivative against the RD‐ES cells and against the enzyme (*K*_i_ = 66 nM). On the other hand, hydrazone **11c**, an analog of **3c**, did not perform well in the cell‐based and enzymatic assays, despite these compounds possess the same 3,5‐dimethylphenyl group. Probably, the bulky 3,5‐dimethylphenyl substituent was deleterious for fitting the phenylacylhydrazone that is also a large group. This hypothesis can be strengthened by comparing the *K*_i_ values of thiosemicarbazone **3a** (*K*_i_ = 199 nM) to hydrazone **11a** (*K*_i_ = 234 nM) that are very similar bearing 2‐pyridinyl group at the 6‐position. In this case, the 2‐pyridinyl group, smaller than the 3,5‐dimethylphenyl, results in compounds with similar activity (**3a** and **11a**).

**Table 3:** IC_50_ values of the most active compounds against GAK.

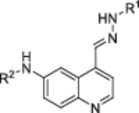				
Entry	R^1^	R^2^	IC_50_ (nM)^a^	Ki (nM)^a^
**3a**	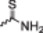	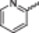	621 ± 50	199 ± 16
**3b**	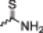	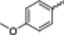	357 ± 37	114 ± 12
**3c**	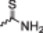	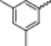	207 ± 11	66 ± 3
**3d**	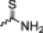	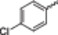	569 ± 101	199 ± 16
**11a**	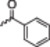	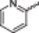	739 ± 93	236 ± 30
**11e**	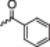	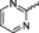	625 ± 115	200 ± 37
**Staur.**	—	—	47 ± 18	15 ± 6

^a^ Data represent the mean ± SEM of at least two independent experiments, with at least two technical replicates each. Compounds were evaluated at a 14‐point serial dilution (twofold dilution).

**Figure 5:**
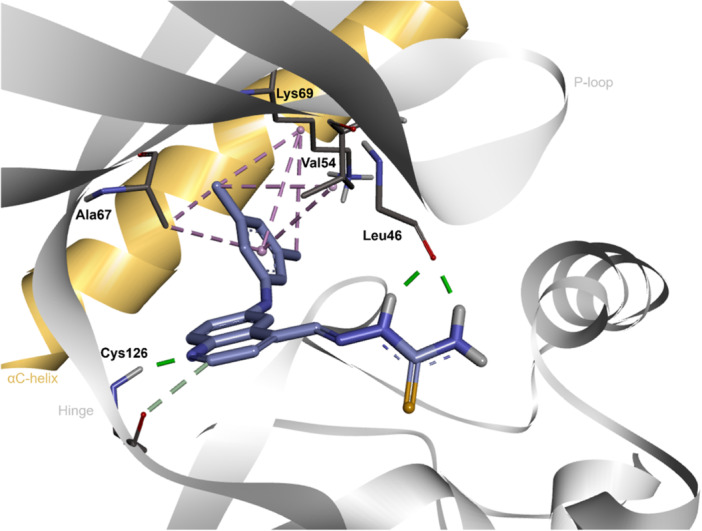
Proposed interactions between **3c** and GAK (PDB: 5Y7Z). Hydrogen bonds are represented by green dashed lines; hydrophobic interactions are represented by pink dashed lines. This figure was prepared using BIOVIA Discovery Studio Visualizer 2025 [45].

To better understand the binding of this new class, we performed an in silico study and proposed the key interactions involved in the molecular recognition of **3c** at the ATP‐binding site of GAK (Figure 5). To assess the accuracy of the docking software, we carried out a redocking experiment using the crystallographic ligand, Gefitinib. The root‐mean‐square deviation (RMSD), which quantifies the distance between the experimental and predicted ligand poses, was used as a parameter, with the value of 2.0 Å set as the cutoff. The obtained RMSD of 1.64 Å indicates a reliable and accurate pose prediction.

For the most active derivative **3c**, we found that the 3,5‐dimethylphenyl substructure fits well in the hydrophobic region I, and it is involved in a complex net of hydrophobic interactions with Val54, Lys6,9 and Ala67. In this way, our major hypothesis is that the binding of this class is guided by two major features that converge with the binding mode of the ligand, Gefitinib. One feature is the accommodation of the hydrophobic substituent in 6‐position of the quinoline scaffold in the hydrophobic region I. The second one is the placement of the quinoline ring in the hinge region, highlighting the H‐bond between the backbone amide group of Cys126 and the quinoline nitrogen. In our study, we observed that several poses of **3c** presented this type of recognition suggesting this is a good proposal. Of note, this compound was the only one able to form two H‐bonds with the backbone amide group of Leu46. The thiosemicarbazone, as a smaller substituent in comparison with hydrazone group, showed more flexibility increasing the potency of **3c**. Indeed, for **11**‐series derivatives docked poses (Figure S8), the hydrazone group at 4‐position accommodates a distinct of thiosemicarbazone of the derivative **3c**. Possibly, this divergence occurs to accommodate the bulky hydrazone group. Based on this, the decreased activity of **11c** could be attributed to the difficulty to fit two bulky groups (6‐dimethylphenyl and 4‐hydrazone) in the active site of GAK. Figure 5 represents the best docked pose for **3c** considering the score, simultaneous presence of more H‐bonds as well as shortest distances of them, and hydrophobic interactions.

## 3 Conclusion

Our cell‐based screening strategy resulted in the discovery of 4,6‐disubstituted quinolines as active compounds with strong effects on cell viability using the ES cell line. Seven compounds displayed promising activity against this cell line in the low micromolar range. Importantly, optimization of the initial hit compound **3a** gave four derivatives with improved potency. Among them, compound **3c** was the most active with an IC_50_ value of 1 µM against RD‐ES. The structure–activity relationship (SAR) study indicated that the cytotoxic activity of the thiosemicarbazone series **3** depended heavily on the appropriate substitution at the 6‐position of the quinoline. Evaluation of selected derivatives against a kinase panel revealed overall selectivity toward the NAK family, consistent with genetic evidence in ES, suggesting that the cell growth inhibition may result from modulation of GAK activity. Six derivatives inhibited this enzyme in the nanomolar range, including **3c**, which was the most active. To the best of our knowledge, this is the first report of compounds that simultaneously inhibit GAK and exhibit activity against ES cells. Together with previous studies that demonstrated GAK as a promising target to bone cancer, this brings the perspective to further investigate in this direction.

## 4 Experimental

### 4.1 Chemistry

#### 4.1.1 General

Reactants were obtained from commercial suppliers and used without further purification. Column chromatography was performed on silica gel Fluka (Sigma‐Aldrich) 0.035‐0.070 mm. Solvents were distilled, when necessary, before use. Melting points were determined on Buchi B‐545 apparatus. The ^1^H and ^13^C NMR spectra were obtained on Bruker 400 nuclear magnetic resonance spectrometer. Proton and carbon shifts (δ) are given with respect to TMS (tetramethylsilane). For new compounds, the purity was determined using Shimadzu 20AT HPLC equipped with a reversed‐phase column (C8, 5 μm, 250 × 4.60 mm, Phenomenex) and Diode Array Detector. The purity and retention time (*t*_R_) are properly indicated in each compound. For NMR characterization, the carbons and hydrogens at 6‐position were attributed as C‘ and H‘, and at 4‐position were attributed as C“ and H“.

#### 4.1.2 Synthesis of 6‐bromo‐4‐methylquinoline (6)

To a stirred solution of 4‐bromoaniline **4** (1 g, 5.8 mmol) in dioxane (10 mL) was added sulfuric acid (0.46 mL, 8.7 mmol) and, after, methyl vinyl ketone **5** (0.75 mL, 8.7 mmol) dropwise. The mixture was kept under reflux for 3 h, and then a second portion of methyl vinyl ketone (0.75 mL) was added dropwise. After 24 h, the mixture was alkalized with a saturated solution of Na_2_CO_3_ (40 mL), and the aqueous layer was extracted with dichloromethane (3 × 50 mL). The combined organic layers were dried over Na_2_SO_4_, filtered, and concentrated under reduced pressure. The crude was purified by silica gel column chromatography using a mixture of *n*‐hexane/EtOAc (8:2) as eluent, affording a brown solid in 43% average yield. ^1^H NMR (400 MHz, CDCl_3_) *δ* (ppm): 8.76 (d, 1H, *J* = 4.4 Hz, H2), 8.12 (t, 1H, *J* = 1.7 Hz, H5), 7.95 (d, 1H, *J* = 8.9 Hz, H8), 7.75 (dd, 1H, *J* = 8.9, 1.7 Hz, H7), 7.23 (d, 1H, *J* = 4.4 Hz, H3), 2.65 (s, 3H, CH_3_).^13^C NMR (100 MHz, CDCl_3_) *δ* (ppm): 150.6 (C2), 146.7 (C8a), 143.6 (C4), 132.7 (C7), 131.9 (C8), 129.6 (C5), 126.4 (C4a), 122.7 (C3), 120.5 (C6), 18.7 (CH_3_). In accordance with ref. [46].

#### 4.1.3 Synthesis of 6‐bromoquinoline‐4‐carbaldehyde (7)

To a stirred solution of 6‐bromo‐4‐methylquinoline **6** (100 mg, 45.0 mmol) in dioxane (5 mL) was added water (0.2 mL) and, after, selenium dioxide (65 mg, 58.5 mmol). The mixture was kept under reflux for 4 h. Then the mixture was filtered on Celite, washed with dichloromethane, and concentrated under reduced pressure. The crude was purified by silica gel column chromatography using a mixture of *n*‐hexane/EtOAc (7:3) as eluent, affording an orange solid in 73% average yield. ^1^H NMR (400 MHz, CDCl_3_) *δ* (ppm): 10.38 (s, 1H, CHO), 9.19 (d, 1H, *J* = 2.2 Hz, H5), 9.16 (d, 1H, *J* = 4.4 Hz, H2), 8.01 (d, 1H, *J* = 9.2 Hz, H8), 7.84 (dd, 1H, *J* = 9.2, 2.2 Hz, H7),7.77 (d, 1H, *J* = 4.4 Hz, H3). ^13^C NMR (100 MHz, CDCl_3_) *δ* (ppm): 192.5 (CHO), 150.7 (C2), 147.7 (C8a), 135.7 (C4), 133.8 (C8), 131.4 (C7), 127.1, 127.0 (C3, C5), 124.6, 124.4 (C6, C4a). In accordance with ref. [15, 47].

#### 4.1.4 Synthesis of Benzhydrazide (10)

To a stirred solution of methyl benzoate **9** (0.93 mL, 7.35 mmol) in absolute ethanol (125 mL) was added hydrazine hydrate 16% (24.5 mL, 117.5 mmol). The reaction was kept under reflux for 3 h. After cooling to room temperature, the mixture was extracted with EtOAc (3 × 30 mL). The organic layers were combined, dried over Na_2_SO_4_, filtered, and concentrated under reduced pressure. The crude was purified by silica gel column chromatography using a mixture of *n*‐hexane:EtOAc (3:7) as eluent, affording a yellowish solid in 53% average yield. ^1^H NMR (400 MHz, CDCl_3_) *δ* (ppm): 7.78–7.72 (m, 3H, NH, H2Phenyl), 7.58–7.47 (m, 1H, H4Phenyl), 7.43 (dd, *J* = 8.3, 6.8 Hz, 2H, H3Phenyl), 4.31–4.01 (m, 2H, NH_2_). ^13^C NMR (100 MHz, CDCl_3_) *δ* (ppm): 168.7 (C═O), 132.6 (C1Phenyl), 131.9 (C4Phenyl), 128.7 (C3Phenyl), 126.9 (C2Phenyl). In accordance with ref. [48].

#### 4.1.5 General Procedure for Hydrazones Derivatives (11a–b, e)

To a stirred solution of 6‐bromo‐4‐carbaldehyde‐quinoline **7** (1.0 eq.) in dioxane (2‐3 mL) were added, in order: Pd(OAc)_2_ (10 mol%), Xantphos (10 mol%), appropriate aniline (2.0 eq.), and Cs_2_CO_3_ (2.0 eq.) in a reaction flask. The mixture was kept at 100°C for 16 h, then allowed to cool to room temperature, filtered on Celite, washed with dichloromethane (30–40 mL), and concentrated under reduced pressure. The crude was purified by silica gel column chromatography using a mixture of *n*‐hexane/EtOAc as eluent, affording the 6‐substituted derivatives **8a**–**b**, and **e** that were used immediately after purification due to the unstable nature of the aldehyde. To a stirred solution of appropriate 6‐arylamine‐4‐carbaldehyde‐quinoline **4** (1.0 eq.) in absolute ethanol (2–6 mL), were added 50–100 µL of glacial acetic acid and benzhydrazide **10** (1.06 eq.) at room temperature. After 4 h, the mixture was alkalized with 10% (w/v) NaHCO_3_ solution (0.5–1.0 mL). Then, 15 mL of brine was added to the mixture, and the aqueous layer was extracted with EtOAc (3 × 20 mL). The combined organic layers were dried over Na_2_SO_4_, filtered, and concentrated under reduced pressure. The crude was purified by silica gel column chromatography using a mixture of *n*‐hexane/EtOAc as eluent, affording **11a–b**, and **e** in 49%–70% yield over two steps.

**(** ***E*** **)‐** ***N*** **′‐((6‐(pyridin‐2‐ylamino)quinolin‐4‐yl)methylene)benzohydrazide (11a):** Starting from **8a** (129.0 mg, 0.52 mmol). Intermediate aldehyde eluent *n*‐hexane:EtOAc (6:4). Compound **11a** eluent *n*‐hexane:EtOAc (4:6). Yellow solid. 49% yield (2 steps). mp: 241°C–244°C. ^1^H NMR (400 MHz, DMSO‐*d*_6_) *δ* (ppm): 12.29 (s, 1H, NH) 9.59 (s, 1H, NH), 9.16–9.14 (m, 2H, CH═N, H5), 8.74 (d, 1H, *J* = 4.4 Hz, H2), 8.34 (d, 1H, *J* = 4.4 Hz, H6py), 8.00–7.98 (m, 3H, H7, H3”), 7.91‐7.82 (m, 2H, H8, H4”), 7.70–7.56 (m, 4H, H8, H4py, H3”), 7.05 (d, 1H, *J* = 7.4 Hz, H3py), 6.86 (dd, 1H, *J* = 7.4, 4.4 Hz, H5py). ^13^C NMR (100 MHz, DMSO‐*d*_6_) *δ* (ppm): 164.0 (C═O), 155.8 (C2py), 148.0 (C2), 147.4, 145.1, 145.0 (C6py, C6, C═NNH), 141.1 (C8a), 138.1 (C4py), 136.3 (C4), 133.6 (C1”), 132.5 (C4”), 130.6, 129.1 (C3”, C4a), 128.3 (C2”), 126.7 (C7), 124.3 (C8), 119.7 (C3), 115.7 (C5py), 111.8 (C5), 107.4 (C3py). HRMS (*m/z*): [MH]^+^ calc for C_22_H_18_N_5_O, 368.1506; found, 368.1502. HPLC: purity > 99%; t_R_ (ACN 50:50 TFA 0.1%): 2.75 min.

**(** ***E*** **)‐** ***N*** **′‐((6‐((4‐methoxyphenyl)amino)quinolin‐4‐yl)methylene)benzohydrazide (11b):** Starting from **8b** (334.0 mg, 1.08 mmol). Intermediate aldehyde eluent *n*‐hexane:EtOAc (8:2). Compound **11b** eluent *n*‐hexane:EtOAc (7:3). Red solid. 70% yield (2 steps). mp: 242°C–244°C. ^1^H NMR (400 MHz, DMSO‐*d*_6_) *δ* (ppm): 12.21 (s, 1H, NH), 8.96 (s, 1H, NH), 8.66–8.55 (m, 2H, H2, CH═N), 8.10 (s, 1H, H3), 7.98 (d, 2H, *J* = 7.2 Hz, H2”), 7.89 (d, 1H, *J* = 9.2 Hz, H8), 7.69 (d, 1H, *J* = 3.2 Hz, H5), 7.65–7.52 (m, 3H, H3”, H4”), 7.48 (dd, 1H, *J* = 9.2, 3.2 Hz, H7), 7.33 (d, 2H, *J* = 8.4 Hz, H2′), 6.99 (d, 2H, *J* = 8.4 Hz, H3′), 3.73 (s, 3H, CH_3_). ^13^C NMR (100 MHz, DMSO‐*d*_6_) δ (ppm): 163.7 (C═O), 154.8 (C4′), 146.0 (C2), 145.8 (C6), 144.6 (C═NNH), 144.5 (C8a), 135.4, 135.1 (C4, C1′), 133.7 (C1”), 132.5 (C4”), 131.3 (C4a), 129.0 (C3”), 128.2 (C2”), 127.1 (C7), 123.2, 121.4, 120.7 (C3, C8, C3”), 115.3 (C2′), 101.3 (C5), 55.6 (OMe). HRMS (*m/z*): [MH]^+^ calc for C_24_H_21_N_4_O_2_, 397.1659; found, 397.1655. HPLC: purity > 99%; t_R_ (ACN 50:50 TFA 0.1%): 4.57 min.

**(** ***E*** **)‐** ***N*** **′‐((6‐(pyrimidin‐2‐ylamino)quinolin‐4‐yl)methylene)benzohydrazide (11e):** Starting from **8e** (33.0 mg, 0.13 mmol). Intermediate aldehyde eluent *n*‐hexane:EtOAc (6:4). Compound **11e** eluent *n*‐hexane:EtOAc (1:1). Yellow solid. 59% yield (2 steps). mp: 248°C–250°C. ^1^H NMR (400 MHz, DMSO‐*d*_6_) *δ* (ppm): 12.27 (s, 1H, NH), 10.14 (s, 1H, NH), 9.28 (s, 1H, CH═N), 9.16 (s, 1H, H5), 8.79 (d, 1H, *J* = 4.4 Hz, H2), 8.62 (d, 2H, *J* = 4.4 Hz, H3′), 8.00–7.97 (m, 4H, H3, H3”, H4”), 7.84 (s, 1H, H8), 7.64‐7.57 (m, 3H, H7, H2”), 6.96 (t, 1H, *J* = 4.4 Hz, H4′). ^13^C NMR (100 MHz, DMSO‐*d*_6_) *δ* (ppm): 163.9 (C═O), 160.3 (C1′), 158.7 (C3′), 148.0 (C2), 145.4, 144.8, 139.9 (C═NNH, C6, C8a), 136.7 (C4), 133.7 (C1”), 132.5 (C4”), 130.4, 129.1 (C3”, C4a), 128.3 (C2”), 126.3 (C7), 124.7 (C8), 119.5 (C3), 113.6 (C4′), 109.4 (C6). HRMS (*m/z*): [MH]^+^ calc for C_21_H_17_N_6_O, 369.1458; found, 369.1452. HPLC: purity > 99%; *t*_R_ (ACN 50:50 TFA 0.1%): 3.09 min.

#### 4.1.6 General Procedure for Hydrazone Derivatives (11c–d, f)

To a stirred solution of 6‐bromo‐4‐carbaldehyde‐quinoline **7** (1.0 eq.) in dioxane (3 mL) were added in order: Pd(OAc)_2_ (10 mol%), Xantphos (10 mol%), appropriate aniline (2.0 eq.), and Cs_2_CO_3_ (2.0 eq.) in a reaction flask. The mixture was kept at 100°C for 16 h, then allowed to cool to room temperature, filtered on Celite, washed with dichloromethane (40 mL), and concentrated under reduced pressure. The crude was purified by silica gel column chromatography using a mixture of *n*‐hexane/EtOAc as eluent, affording the 6‐substituted derivatives **8c–d**, and **f** that were used immediately after purification due to the unstable nature of the aldehyde. To a stirred solution of appropriate 6‐arylamine‐4‐carbaldehyde‐quinoline (1.0 eq.) in absolute ethanol (5 mL), were added 50 µL of glacial acetic acid and benzhydrazide **10** (1.06 eq.) at room temperature. The reaction was monitored by TLC, and upon precipitation of the product (after 3–4 h), the mixture was filtered. The precipitated products were obtained in sufficient purity to be used without further purification, affording **11c–d**, and **f** in 44%–60% yield over two steps.

**(** ***E*** **)‐** ***N*** **′‐((6‐((3,5‐dimethylphenyl)amino)quinolin‐4‐yl)methylene)benzohydrazide (11c):** Starting from **8c** (33.0 mg, 0.12 mmol). Intermediate aldehyde eluent *n*‐hexane:EtOAc (7:3). Orange solid. 60% yield (2 steps). mp: 240°C–242°C. ^1^H NMR (400 MHz, DMSO‐*d*_6_) *δ* (ppm): 12.15 (s, 1H, NH), 9.01 (s, 1H, NH), 8.68 (d, 1H, *J* = 4.5 Hz, H2), 8.60 (s, 1H, H5), 8.13 (s, 1H, CH═N), 7.95–7.92 (m, 3H, H2”, H8), 7.74 (d, *J* = 4.5 Hz, 1H, H3), 7.73–7.53 (m, 4H, H7, H3”, H4”), 6.90 (s, 2H, H2′), 6.59 (s, 1H, H4′), 2.25 (s, 6H, CH_3_).^13^C NMR (100 MHz, DMSO‐*d*_6_) *δ* (ppm): 163.2 (C═O), 146.2 (C2), 145.0, 144.3, 143.0, (C6, C═NNH, C8a), 142.2 (C1′), 138.4 (C3′), 135.0 (C4), 133.2 (C1”), 132.0 (C4”), 130.9 (C4a), 128.6 (C3”), 127.7 (C2”), 126.5 (C7), 123.3, 123.0 (C8, C4′), 119.6 (C3), 115.7 (C2′), 103.8 (C5), 21.2 (CH_3_). HRMS (*m/z*): [MH]^+^ calc for C_25_H_23_N_4_O, 395.1866; found, 395.1864. HPLC: purity > 99%; t_R_ (ACN 50:50 TFA 0.1%): 6.76 min.

**(** ***E*** **)‐** ***N*** **′‐((6‐((4‐chlorophenyl)amino)quinolin‐4‐yl)methylene)benzohydrazide (11 d):** Starting from **8d** (90.0 mg, 0.32 mmol). Intermediate aldehyde eluent *n*‐hexane:EtOAc (8:2). Orange solid. 55% yield (2 steps). mp: 240°C–244°C. ^1^H NMR (400 MHz, DMSO‐*d*_6_) *δ* (ppm): 12.16 (s, 1H, NH), 8.96 (s, 1H, NH), 8.91 (s, 1H, CH═N), 8.71 (d, 1H, *J* = 4.4 Hz, H2), 8.47 (s, 1H, H5); 7.98–7.96 (m, 3H, H3, H2”), 7.69–7.54 (m, 5H, H7, H8, H3”, H4”), 7.42 (s, 4H, H2′, H3′). ^13^C NMR (100 MHz, DMSO‐*d*_6_) *δ* (ppm): 163.8 (C═O), 146.9, 146.5 (C2, C1′), 144.8, 142.7, 141.6 (C‐6, C═NNH, C8a), 135.4 (C4), 133.7 (C1”), 132.5 (C4”), 131.4 (C4a), 129.8 (C3′), 129.1 (C3”), 128.2 (C2”), 126.4 (C7), 124.3, 123.9, 121.9 (C3, C8, C4′), 119.5 (C2′), 104.5 (C5). HRMS (*m/z*): [MH]^+^ calc for C_23_H_18_ClN_4_O, 401.1164; found, 401.1159. HPLC: purity 99%; *t*_R_ (ACN 50:50 TFA 0.1%): 6.34 min.

**(** ***E*** **)‐** ***N*** **′‐((6‐((4,6‐dimethoxypyrimidin‐2‐yl)amino)quinolin‐4‐yl)methylene)benzohydrazide (11 f):** Starting from **8f** (25.0 mg, 0.08 mmol). Intermediate aldehyde eluent *n*‐hexane:EtOAc (7:3). Yellow solid. 44% yield (2 steps). mp: 257°C–259°C. ^1^H NMR (400 MHz, DMSO‐*d*_6_) *δ* (ppm): 12.22 (s, 1H, NH), 9.99 (s, 1H, NH), 9.17 (s, 1H, CH═N), 9.03 (s, 1H, H5), 8.81 (d, 1H, *J* = 3.8 Hz, H2), 8.03–7.95 (m, 4H, H8, H3”, H4”), 7.80 (d, 1H, *J* = 3.8 Hz, H3), 7.66–7.57 (m, 3H, H7, H2”), 5.71 (s, 1H, py), 3.91 (s, 6H, CH_3_). ^13^C NMR (100 MHz, DMSO‐*d*_6_) *δ* (ppm): 171.9 (C3′), 163.9 (C═O), 159.2 (C1′), 148.2 (C2), 145.2, 145.0, 139.6 (C6, C8a, C = NNH), 137.1 (C4), 133.6 (C1”), 132.6 (C4”), 130.3, 129.1 (C3”, C4a), 128.1 (C2”), 126.2 (C7), 125.1 (C8), 119.5 (C3), 110.6 (C5), 81.7 (C4′), 54.2 (OMe). HRMS (*m/z*): [MH]^+^ calc for C_23_H_21_N_6_O_3_, 429.1670; found, 429.1669. HPLC: purity > 99%; t_R_ (ACN 50:50 TFA 0.1%): 4.48 min.

#### 4.1.7 Synthesis of 2‐chloro‐3,5‐dinitropyridine (14)

In a Schlenk flask were added, as following: 2‐hydroxy‐3,5‐nitropyridine **13** (800 mg, 4.3 mmol), freshly distilled oxychloride phosphorus (2.5 mL, 27.5 mmol), and DMF dropwise (240 µL, 3.1 mmol). The mixture was kept at 105°C under stirring for 16 h. Then, it was allowed to cool at room temperature and neutralized with a saturated solution of NaHCO_3_ (32 mL). The aqueous layer was extracted with dichloromethane (2 × 25 mL), and the organic layers were combined, dried over Na_2_SO_4_, filtered, and concentrated under reduced pressure. The crude was purified by silica gel column chromatography using a mixture of *n*‐hexane/EtOAc (7:3) as eluent, affording a yellow solid in 93% yield. ^1^H NMR (400 MHz, CD_3_OD) *δ* (ppm): 9.45 (d, 1H, *J* = 2.4 Hz, H6py), 9.22 (d, 1H, *J* = 2.4 Hz, H4py). ^13^C NMR (100 MHz, CD_3_OD) *δ* (ppm): 148.7 (C6py), 148.3 (C3py), 146.4 (C2py), 135.4 (C4py), 131.3 (C5py). In accordance with ref. [49].

#### 4.1.8 Synthesis of 2‐chloropyridine‐3,5‐diamine (15)

To a stirred solution of 2‐chloro‐3,5‐dinitropyridine **14** (100 mg, 0.5 mmol) in absolute ethanol (5 mL) tin (II) chloride (932 mg, 5 mmol) was added. The mixture was kept under reflux for 2 h. Then, 5 mL of NaOH 2.5 M solution was added, and the aqueous layer was extracted with EtOAc (3 × 15 mL). The organic layers were combined, dried over Na_2_SO_4_, filtered, and concentrated under reduced pressure. The crude was purified by silica gel column chromatography using a mixture of *n*‐hexane/EtOAc (50% to 100%) as eluent (1:1—150 mL; 100% EtOAc—100 mL), affording a pale‐yellow solid in 75% yield. ^1^H NMR (400 MHz, CD_3_OD) *δ* (ppm): 7.13 (d, 1H, *J* = 2.4 Hz, H6py), 6.53 (d, 1H, *J* = 2.4 Hz, H4). ^13^C NMR (100 MHz, CD_3_OD) δ (ppm): 146.5 (C6py), 142.6 (C2py), 126.0 (C3py), 125.8 (C4py), 109.6 (C5py). In accordance with ref. [49].

#### 4.1.9 General Procedure for Sulfonamide Formation

To a stirred solution of appropriate amine (1.0 eq.) in absolute ethanol (2 mL) were added appropriate sulfonyl chloride (1.0 eq.) and, after, pyridine (4.0 eq.). The reaction was kept under reflux temperature for 18 h. Afterwards, was allowed to cool at room temperature, EtOAc (15–25 mL) and water (15–25 mL) were added, and the pH was alkalinized to 8 with NaHCO_3_ solution (0.5–1.5 mL), and the aqueous layer was extracted two times with EtOAc (15–25 mL). The organic layers were combined, dried over Na_2_SO_4_, filtered, and concentrated under reduced pressure. The crude was purified by silica gel column chromatography using a mixture of *n*‐hexane/EtOAc as eluent, affording the sulfonamides in 13%–36% yield.

***N*** **‐(5‐amino‐6‐chloropyridin‐3‐yl)−4‐methylbenzenesulfonamide (16):** Starting from **15** (199.0 mg, 1.83 mmol). Eluent *n*‐hexane:EtOAc (7:3). White solid. 36% yield. mp: 168°C–170°C. ^1^H NMR (400 MHz, CD_3_OD) *δ* (ppm): 7.66 (d, 2H, *J* = 8.2 Hz, H2phenyl), 7.32 (d, 2H, *J* = 8.2 Hz, H3phenyl), 7.26 (d, 1H, *J* = 2.4 Hz, H2py), 7.06 (d, 1H, *J* = 2.4 Hz, H4py), 2.37 (s, 3H, CH_3_). ^13^C NMR (100 MHz, CD_3_OD) *δ* (ppm): 145.6 (C5py), 143.2 (C3py), 137.8 (C4phenyl), 136.7 (C1phenyl), 132.5 (C5py), 130.9 (C2phenyl), 129.9 (C6py), 128.4 (C3phenyl), 115.5 (C5py), 21.6 (CH_3_). HRMS (*m/z*): [MH]^+^ calc for C_12_H_13_ClN_3_O_2_S, 298.0412; found, 298.0410. HPLC: purity > 99%; t_R_ (ACN 50:50 TFA 0.1%): 6.63 min.

***N*** **‐(6‐aminopyridin‐2‐yl)−4‐methylbenzenesulfonamide (18):** Starting from **17** (76.0 mg, 0.53 mmol). Eluent *n*‐hexane:EtOAc (8:2—100 mL; 7:3—100 mL; 100% EtOAc—50 mL). White solid. 13% yield. mp: 206°C–208°C. ^1^H NMR (400 MHz, CD_3_OD) *δ* (ppm): 7.66 (d, 2H, *J* = 8.2 Hz, H2phenyl), 7.63 (d, 1H, *J* = 2.4 Hz, H3py), 7.43 (d, 1H, *J* = 2.4 Hz, H5py), 7.31 (d, 2H, *J* = 8.2 Hz, H3phenyl), 6.95 (t, 1H, *J* = 2.4 Hz, H4py), 2.37 (s, 3H, CH_3_). ^13^C NMR (100 MHz, CD_3_OD) *δ* (ppm): 146.9 (C2py), 145.3 (C6py), 137.8 (C4py), 136.9, 133.2, 130.7 (C3py, C5py, C1phenyl), 130.6 (C2phenyl), 128.2 (C3phenyl), 114.4 (C4phenyl), 21.4 (CH_3_). HRMS (*m/z*): [MH]^+^ calc for C_12_H_14_N_3_O_2_S, 264.0801; found, 264.0799. HPLC: purity > 99%; t_R_ (ACN 50:50 TFA 0.1%): 3.81 min.

#### 4.1.10 General Procedure for Thiosemicarbazone Derivatives (3a–f)

To a stirred solution of 6‐bromo‐4‐carbaldehyde‐quinoline **7** (1.0 eq.) in dioxane (3 mL) were added in order: Pd(OAc)_2_ (10 mol%), Xantphos (10 mol%), appropriate aniline (2.0 eq.), and Cs_2_CO_3_ (2.0 eq.) in a reaction flask. The mixture was kept at 100°C for 16 h, then allowed to cool to room temperature, filtered on Celite, washed with dichloromethane (30 mL), and concentrated under reduced pressure. The crude was purified by silica gel column chromatography using a mixture of *n*‐hexane/EtOAc as eluent, affording the 6‐substituted derivatives **8a–d, g–h** that were used immediately after purification due to the unstable nature of the aldehyde. To a stirred solution of appropriate 6‐arylamine‐4‐carbaldehyde‐quinoline **8** (1.0 eq.) in absolute ethanol (5 mL), were added 50 µL of glacial acetic acid and thiosemicarbazide (1.0 eq.) under reflux. The reaction was monitored by TLC, and after the precipitate formation, between 2 and 3 h, the mixture was filtered off and washed with cold deionized water (5–10 mL). The derivatives were obtained in 38%–53% yield over two steps. The precipitated products were sufficiently pure and did not require further purification.

**(** ***E*** **)−2‐((6‐(pyridin‐2‐ylamino)quinolin‐4‐yl)methylene)hydrazine‐1‐carbothioamide (3a):** Starting from **8a** (71.6 mg, 0.29 mmol). Intermediate aldehyde eluent *n*‐hexane:EtOAc (6:4). Yellow solid. 51% yield (2 steps). mp: 241°C–243°C. ^1^H NMR (400 MHz, DMSO‐*d*_6_) *δ* (ppm): 11.94 (s, 1H, NH), 9.59 (s, 1H, NH), 9.17 (d, *J* = 2.2 Hz, 1H, H5), 8.77 (s, 1H, CH═N), 8.69 (d, *J* = 4.6 Hz, 1H, H2), 8.56 (s, 1H, NH2), 8.32 (dd, *J* = 5.0, 1.4 Hz, 1H, H6py), 8.14 (s, 1H, NH_2_), 8.04 (d, *J* = 4.6 Hz, 1H, H3), 7.96 (d, *J* = 9.1 Hz, 1H, H8), 7.81 (dd, *J* = 9.1, 2.2 Hz, 1H, H7), 7.68–7.63 (m, 1H, H4py), 6.98 (d, *J* = 8.4 Hz, 1H, H3py), 6.89–6.85 (m, 1H, H5py). ^13^C NMR (100 MHz, DMSO‐*d*_6_) *δ* (ppm): 178.4 (C═S), 155.4 (C2py), 147.5 (C6py), 146.7 (C2), 144.6 (C6), 140.5, 138.9, 137.5 (C═NNH, C8a, C4py), 135.5, 130.2 (C4, C4a), 126.2 (C7), 123.6 (C8), 119.1, 115.3, 111.6 (C3, C3py, C5py), 106.6 (C5). HRMS (*m/z*): [MH]^+^ calc for C_16_H_15_N_6_S, 323.1073; found, 323.1062. HPLC: purity 99%; t_R_ (ACN 50:50 TFA 0.1%): 2.46 min.

**(** ***E*** **)−2‐((6‐((4‐methoxyphenyl)amino)quinolin‐4‐yl)methylene)hydrazine‐1‐carbothioamide (3b):** Starting from **8b** (25.6 mg, 0.09 mmol). Intermediate aldehyde eluent *n*‐hexane:EtOAc (8:2). Orange solid. 47% yield (2 steps). mp: 238°C–240°C. ^1^H (400 MHz, DMSO‐*d*_6_) δ (ppm): 11.72 (s, 1H, NH), 8.65 (s, 1H, CH═N), 8.56 (d, 1H, *J* = 4.8 Hz, H2), 8.50 (s, 1H, NH), 8.44 (s, 1H, NH), 8.06 (s, 1H, NH), 7.97 (d, 1H, *J* = 4.8 Hz, H3), 7.87 (d, 1H, *J* = 9.2 Hz, H8), 7.60 (d, 1H, *J* = 2.4 Hz, H5), 7.42 (dd, 1H, *J* = 9.2, 2.4 Hz, H7), 7.23 (d, 2H, *J* = 8.8 Hz, H2′), 6.97 (d, 2H, *J* = 8.8 Hz, H3′), 3.76 (s, 3H, CH_3_). ^13^C NMR (100 MHz, DMSO‐*d*_6_) δ (ppm): 178.7 (C═S), 155.3 (C4′), 145.9 (C2), 144.8, 144.5, 139.2 (C6, C═NNH, C8a), 135.2, 134.9 (C4, C1′), 131.5 (C4a), 127.5 (C7), 122.9 (C8), 122.3 (C8), 119.1 (C3), 115.3 (C2′), 100.2 (C5), 55.8 (CH_3_). HRMS (*m/z*): [MH]^+^ calc for C_18_H_18_N_5_OS, 352.1227; found, 352.1221. HPLC: purity > 99%; t_R_ (ACN 50:50 TFA 0.1%): 3.48 min.

**(** ***E*** **)−2‐((6‐((3,5‐dimethylphenyl)amino)quinolin‐4‐yl)methylene)hydrazine‐1‐carbothioamide (3c):** Starting from **8c** (49.2 mg, 0.18 mmol). Intermediate aldehyde eluent *n*‐hexane:EtOAc (7:3). Yellow solid. 53% yield (2 steps). mp: 264°C–266°C. ^1^H NMR (400 MHz, DMSO‐*d*_6_) *δ* (ppm): 11.72 (s, 1H, NH), 8.68 (s, 1H, CH═N), 8.64–8.59 (m, 2H, NH, H2), 8.41 (s, 1H, NH), 8.07–8.00 (m, 2H, NH, H3), 7.91 (d, 1H, *J* = 9.2 Hz, H8), 7.78 (d, 1H, *J* = 2.6 Hz, H5), 7.53 (dd, 1H, *J* = 9.2, 2.6 Hz, H7), 6.86 (s, 2H, H2′), 6.62 (s, 1H, H4′), 2.27 (s, 6H, CH_3_). ^13^C NMR (100 MHz, DMSO‐*d*_6_) δ (ppm): 178.2 (C═S), 145.9 (C2), 144.0, 143.0, 142.0 (C‐6, C═NNH, C8a), 138.5 (C3′), 138.4 (C1′), 134.9 (C4), 130.8 (C4a), 126.7 (C7), 123.2, 123.1 (C8, C4′), 118.5 (C3), 116.1 (C2′), 102.8 (C5), 21.2 (CH_3_). HRMS (*m/z*): [MH]^+^ calc for C_19_H_20_N_5_S, 350.1434; found, 350.1436. HPLC: purity > 99%; *t*_R_ (ACN 50:50 TFA 0.1%): 4.53 min.

**(** ***E*** **)−2‐((6‐((4‐chlorophenyl)amino)quinolin‐4‐yl)methylene)hydrazine‐1‐carbothioamide (3d):** Starting from **8d** (71.0 mg, 0.25 mmol). Intermediate aldehyde eluent *n*‐hexane:EtOAc (8:2). Orange solid. 52% yield (two steps). mp: 236°C–239°C. ^1^H NMR (400 MHz, DMSO‐*d*_6_) *δ* (ppm): 11.70 (s, 1H, NH), 8.88 (s, 1H, CH═N), 8.70 (s, 1H, NH), 8.64 (d, 1H, *J* = 4.4 Hz, H2), 8.47 (s, 1H, NH), 8.12 (s, 1H, NH), 8.04 (d, 1H, *J* = 4.4 Hz, H3), 7.94 (d, 1H, *J* = 9.2 Hz, H8), 7.80 (d, 1H, *J* = 2.4 Hz, H5), 7.51 (dd, 1H, *J* = 8.8, 2.4 Hz, H7), 7.38 (d, 2H, *J* = 8.8 Hz, H2′), 7.29 (d, 2H, *J* = 9.2 Hz, H3′). ^13^C NMR (100 MHz, DMSO‐*d*_6_) *δ* (ppm): 178.7 (C═S), 146.9 (C2), 144.9, 142.5, 141.6 (C6, C═NNH, C8a), 138.7 (C1′), 135.4 (C4), 131.6 (C4a), 129.8 (C3′), 127.0 (C4′), 124.8, 123.7 (C7, C8), 120.0 (C2′), 118.9 (C3), 103.1 (C5). HRMS (*m/z*): [MH]^+^ calc for C_17_H_15_ClN_5_S, 356.0731; found, 356.0726. HPLC: purity 99%; t_R_ (ACN 50:50 TFA 0.1%): 4.27 min.

(*E*)−2‐((6‐((2‐chloro‐5‐((4‐methylphenyl)sulfonamido)pyridin‐3‐yl)amino)quinolin‐4‐yl)methylene)hydrazine‐1‐carbothioamide (**3e**): Starting from **8g** (22.0 mg, 0.049 mmol). Intermediate aldehyde eluent *n*‐hexane:EtOAc (1:1—100 mL; 3:7—50 mL; 100% EtOAc—50 mL). Orange solid. 38% yield (two steps). mp: 261°C–263°C. ^1^H NMR (400 MHz, DMSO‐*d*_6_) *δ* (ppm): 11.67 (s, 1H, NH), 10.61 (s, 1H, NH), 8.77 (d, 1H, *J* = 4.6 Hz, H2), 8.66 (s, 1H, NH), 8.50 (s, 1H, CH═N), 8.47 (s, 1H, NH_2_), 8.14 (s, 1H, NH_2_), 8.09 (d, 1H, *J* = 4.6 Hz, H3), 7.99 (d, 1H, *J* = 9.2 Hz, H8), 7.83 (d, 1H, *J* = 2.5 Hz, H6py), 7.77 (d, 1H, *J* = 2.5 Hz, H4py), 7.60 (d, 2H, *J* = 8.0 Hz, Phenyl), 7.48 (dd, 1H, *J* = 9.2 2.5 Hz, H7), 7.40 (d, 1H, *J* = 2.5 Hz, H5), 7.33 (d, 2H, *J* = 8.0 Hz, Phenyl), 2.33 (s, 3H, CH_3_). ^13^C NMR (100 MHz, DMSO‐*d*_6_) *δ* (ppm): 178.3 (C═S), 147.8 (C2), 145.3, 143.9, 140.3 (C6, C═NNH, C8a), 138.5, 137.1 (C1Phenyl, C4Phenyl), 136.0 (C4), 135.8, 135.1, 134.9 (C3py, C5py, C6py), 132.2 (C2py), 130.9 (C4a), 130.0 (C3Phenyl), 126.7 (C2Phenyl), 126.0, 124.0 (C7, C8), 118.9 (C3), 116.4 (C4py), 109.4 (C5), 21.0 (CH_3_). HRMS (*m/z*): [MH]^+^ calc for C_23_H_21_ClN_7_O_2_S_2_, 526.0881; found, 526.0879. HPLC: purity 90%; t_R_ (ACN 50:50 TFA 0.1%): 3.86 min.

**(** ***E*** **)−2‐((6‐((6‐((4‐methylphenyl)sulfonamido)pyridin‐2‐yl)amino)quinolin‐4‐yl)methylene)hydrazine‐1‐carbothioamide (3f):** Starting from **8h** (70.0 mg, 0.17 mmol). Intermediate aldehyde eluent *n*‐hexane:EtOAc (7:3—150 mL; 1:1—50 mL; 4:6—100 mL; 2:8—50 mL; 100% EtOAc—30 mL). Orange solid. 42% yield (two steps). mp: 250°C–252°C. ^1^H NMR (400 MHz, DMSO‐*d*_6_) *δ* (ppm): 9.42 (s, 1H, NH), 8.80 (s, 1H, CH═N), 8.69 (d, 1H, *J* = 4.8 Hz, H2), 8.47 (s, 1H, H5), 8.35 (s, 1H, NH_2_), 8.22 (dd, 1H, *J* = 9.2, 2.4 Hz, H7), 8.16 (s, 1H, NH_2_), 8.06 (d, 1H, *J* = 4.8 Hz, H3), 7.91 (d, 1H, *J* = 9.2 Hz, H8), 7.71 (d, 2H, *J* = 8.3 Hz, Phenyl), 7.47 (t, 1H, *J* = 7.7 Hz, H4py), 7.16 (d, 2H, *J* = 8.3 Hz, Phenyl), 6.48 (d, 1H, *J* = 8.3 Hz, H3py), 6.46 (d, 1H, *J* = 7.7 Hz, H5py), 2.22 (s, 3H, CH_3_). ^13^C NMR (100 MHz, DMSO‐*d*_6_) *δ* (ppm): 178.7 (C═S), 154.5 (C2py), 151.3 (C6py), 147.3 (C2), 145.0, 143.1, 140.8 (C6, C═NNH, C8a), 139.7, 139.6 (C4Phenyl, C4py), 138.8 (C1Phenyl), 135.9 (C4), 130.5 (C4a), 129.7 (H3Phenyl), 127.4 (H2Phenyl), 126.5, 124.1 (C7, C8), 118.8 (C3), 107.7 (C5py), 105.2 (C3py), 103.4 (C5), 21.3 (CH_3_). HRMS (*m/z*): [MH]^+^ calc for C_23_H_22_N_7_O_2_S_2_, 492.1271; found, 492.1272. HPLC: purity 98%; t_R_ (ACN 50:50 TFA 0.1%): 4.01 min.

#### 4.1.11 General Procedure for Thiazole Derivatives (12a–e)

To a stirred solution of the appropriate thiosemicarbazone 3 (1.0 eq.) in isopropanol (2 mL) was added 2‐bromoacetophenone (1.0 eq.). The reaction was kept under reflux for 1–2 h and monitored by TLC. Upon precipitation of the product, the solid was collected by filtration through a Büchner funnel, washed with saturated NaHCO₃ solution (1 mL), and thoroughly washed with cold deionized water (10 mL). The precipitated products were sufficiently pure and did not require further purification.

**(** ***E*** **)−4‐{[2‐(4‐Phenylthiazol‐2‐yl)hydrazono]methyl}‐** ***N*** **‐(pyridin‐2‐yl)quinolin‐6‐amine (12a):** Starting from **3a** (35.0 mg, 0.11 mmol). Brownish solid. 86% yield. mp: 237°C–239°C. ^1^H NMR (400 MHz, DMSO‐*d*_6_) *δ* (ppm): 13.24 (s, 1H, NH), 9.95 (s, 1H, NH), 9.38 (d, 1H, *J* = 1.6 Hz, H5), 8.82 (d, 1H, *J* = 5.2 Hz, H2), 8.74 (s, 1H, CH═N), 8.35 (dd, 1H, *J* = 5.2, 1.6 Hz, H6py), 8.09 (d, 1H, *J* = 8.9 Hz, H8), 8.05–7.98 (m, 2H, H3, H7), 7.89 (dd, 2H, *J* = 7.1, 1.6 Hz, H2”), 7.49 (s, 1H, Hthiazole), 7.72 (ddd, 1H, *J* = 8.9, 7.1, 1.6 Hz, H4py), 7.43 (t, 2H, *J* = 7.2 Hz, H3”), 7.33 (t, 1H, *J* = 7.2 Hz, H4”), 7.06 (d, 1H, *J* = 8.4 Hz, H3py), 6.95 (dd, 1H, *J* = 7.2, 5.2 Hz, H5py). ^13^C NMR (100 MHz, DMSO‐*d*_6_) *δ* (ppm): 167.7 (Cthiazole), 155.4 (C2Py), 151.3 (CHSthiazole), 147.5 (C6py), 142.3 (C2), 142.1, 142.0, 138.4 (C6, C═NNH, C8a), 138.2, 135.0, 134.7 (C4, C1”, C4py), 129.2 (C3”), 128.3, 127.0, 126.9 (C4a, C7, C4”), 126.0 (C2”), 125.2 (C8), 117.0 (C5py), 116.4 (C3), 112.6 (C3py), 106.8 (CHNthiazole), 105.8 (C5). HRMS (*m/z*): [MH]^+^ calc for C_24_H_19_N_6_S, 423.1386; found, 423.1380. HPLC: purity 99%; t_R_ (MeOH 80:20 TFA 0.1%): 5.03 min.

**(** ***E*** **)‐** ***N*** **‐(4‐methoxyphenyl)−4‐((2‐(4‐phenylthiazol‐2‐yl)hydrazineylidene)methyl)quinolin‐6‐amine (12b):** Starting from **3b** (40.0 mg, 0.12 mmol). Brownish solid. 88% yield. mp: 240°C–243°C. ^1^H NMR (400 MHz, DMSO‐*d*_6_) *δ* (ppm): 9.01 (s, 1H, NH), 8.73 (d, 1H, *J* = 5.8 Hz, H2), 8.48 (s, 1H, CH═N), 8.05 (d, 1H, *J* = 9.3 Hz, H8), 7.96 (d, 1H, *J* = 5.8 Hz, H3), 7.92 (d, 1H, *J* = 2.5 Hz, H8), 7.85 (d, 2H, *J* = 8.0 Hz, H2′), 7.72 (dd, 1H, *J* = 9.3, 2.5 Hz, H7), 7.44–7.28 (m, 6H, H2”, H3”, H4”, CHthiazole), 6.99 (d, 2H, *J* = 8.0 Hz, H3′), 3.75 (s, 3H, CH_3_). ^13^C NMR (100 MHz, DMSO‐*d*_6_) *δ* (ppm): 167.5 (Cthiazole), 156.4, 147.5 (C2, C4′), 142.2 (CHNthiazole), 138.4 (C6), 136.1, 134.7, 133.7 (C4, C1′, C1”), 129.2 (C3”), 128.3, 127.6, 126.8 (C4a, C7, C4”), 126.0 (C2”), 123.9 (C2′), 123.8 (C8), 119.0 (C3), 115.5 (C3′), 105.8 (C5), 100.3 (CHSthiazole), 55.7 (CH_3_). HRMS (*m/z*): [MH]^+^ calc for C_26_H_22_N_5_OS, 452.1540; found, 452.1530. HPLC: purity 99%; t_R_ (MeOH 80:20 TFA 0.1%): 5.95 min.

**(** ***E*** **)‐** ***N*** **‐(3,5‐dimethylphenyl)−4‐((2‐(4‐phenylthiazol‐2‐yl)hydrazineylidene)methyl)quinolin‐6‐amine (12c):** Starting from **3c** (44.0 mg, 0.13 mmol). Brownish solid. 90% yield. mp: 235°C–237°C. ^1^H NMR (400 MHz, DMSO‐*d*_6_) *δ* (ppm): 9.04 (s, 1H, NH), 8.75 (d, 1H, *J* = 5.6 Hz, H2), 8.50 (s, 1H, CH═N), 8.14 (d, 1H, *J* = 2.2 Hz, H5), 8.05 (d, 1H, *J* = 9.2 Hz, H8), 7.95 (d, 1H, *J* = 5.6 Hz, H3), 7.86 (d, 2H, *J* = 7.6 Hz, H2”), 7.76 (dd, 1H, *J* = 9.2, 2.2 Hz, H7), 7.42–7.29 (m, 4H, H3”, H4”, CHthiazole), 6.95 (s, 2H, H1′), 6.72 (s, 1H, H4′), 2.26 (s, 6H, CH_3_). ^13^C NMR (100 MHz, DMSO‐*d*_6_) *δ* (ppm): 167.7 (Cthiazole), 151.3 (CHSthiazole), 146.0 (C2), 142.0, 141.2, 139.6 (C6, C═NNH, C8a), 139.3 (C3′), 136.0, 135.7, 134.7 (C4, C1′, C1”), 129.2 (C3”), 128.3, 127.4, 127.0 (C4a, C7, C4”), 126.0 (C2”), 125.3, 124.4 (C8, C4′), 118.9 (C3), 118.1 (C2′), 105.9 (CHNthiazole), 102.4 (C5), 21.6 (CH_3_). HRMS (*m/z*): [MH]^+^ calc for C_27_H_24_N_5_S, 450.1747; found, 450.1747. HPLC: purity 99%; t_R_ (MeOH 80:20 TFA 0.1%): 8.82 min.

**(** ***E*** **)‐** ***N*** **‐(4‐chlorophenyl)−4‐((2‐(4‐phenylthiazol‐2‐yl)hydrazineylidene)methyl)quinolin‐6‐amine (12d):** Starting from **3d** (15.0 mg, 0.042 mmol). Brownish solid. 83% yield. mp: 240°C–242°C. ^1^H NMR (400 MHz, DMSO‐*d*_6_) *δ* (ppm): 8.79 (s, 1H, NH), 8.63 (d, 1H, *J* = 4.6 Hz, H2), 8.48 (s, 1H, CH═N), 8.21 (d, 1H, *J* = 2.0 Hz, H5), 7.92 (d, 1H, *J* = 8.3 Hz, H8), 7.85 (dd, 2H, *J* = 8.4, 2.0 Hz, H2”), 7.61 (d, 1H, *J* = 4.6 Hz, H3), 7.51 (dd, 1H, *J* = 8.4, 2.0 Hz, H7), 7.40 (t, 2H, *J* = 8.3 Hz,), 7.35–7.27 (m, 6H, H2′, H3′, H4”), 7.23 (s, 1H, Hthiazole). ^13^C NMR (100 MHz, DMSO‐d_6_) *δ* (ppm): 150.6 (CHNthiazole), 146.6 (C2), 144.6 (C6), 142.2, 141.6, 137.1 (C1′ C═NNH, C8a), 135.4, 134.9 (C4, C1′), 131.0 (C4a), 129.3 (C3′), 128.6 (C3”), 127.4, 125.8 (C4′, C4”), 125.5 (C2”), 124.3 (C4′), 123.1 (C7), 119.8 (C8), 118.6 (C3), 104.8 (CHSthiazole), 103.5 (C5). HRMS (*m/z*): [MH + ] calc for C_25_H_19_ClN_5_S, 456.1044; found, 456.1034. HPLC: purity 98%; t_R_ (MeOH 80:20 TFA 0.1%): 7.48 min.

(*E*)−4‐methyl‐*N*‐(6‐((4‐((2‐(4‐phenylthiazol‐2‐yl)hydrazineylidene)methyl)quinolin‐6‐yl)amino)pyridin‐2‐yl)benzenesulfonamide (**12e**): Starting from **3f** (25.0 mg, 0.051 mmol). Brownish solid. 85% yield. mp: 248°C–250°C. ^1^H NMR (400 MHz, DMSO‐*d*_6_) *δ* (ppm): 10.87 (s, 1H, NH), 9.87 (s, 1H, NH), 8.91 (d, 1H, *J* = 5.6 Hz, H2), 8.72 (s, 1H, CH = N), 8.58 (d, 1H, *J* = 9.2, 2.3 Hz, H7), 8.52 (d, 1H, *J* = 2.3 Hz, H5), 8.14 (d, 1H, *J* = 5.6 Hz, H3), 8.10 (d, 1H, *J* = 9.2 Hz, H8), 7.89 (d, 2H, *J* = 7.2 Hz, H2”), 7.68 (d, 2H, *J* = 8.4 Hz, Phenyl), 7.56 (t, 1H, *J* = 8.0 Hz, H4Py), 7.51 (s, 1H, CHthiazole), 7.46–7.42 (m, 2H, H3”), 7.35–7.32 (m, 1H, H4”), 7.19 (d, 2H, *J* = 8.4 Hz, Phenyl), 6.60 (d, 1H, *J* = 8.0 Hz, Py), 6.54 (d, 1H, *J* = 7.2 Hz, Py), 2.23 (s, 3H, CH_3_). ^13^C NMR (100 MHz, DMSO‐*d*_6_) *δ* (ppm): 153.4 (C2py, C6py), 149.8 (C2), 143.3, 142.1 (C8a, C = NNH), 139.9, 139.8, 137.3 (C4py, C4Phenyl′, C1Phenyl′), 134.6, 134.2 (C4, C1Phenyl”), 129.4, 128.8 (C3Phenyl′, C3Phenyl”), 127.9, 127.3 (C4a, C4Phenyl”), 127.0, 126.3, 125.6, 123.1 (C7, C8, C2Phenyl′, C2Phenyl”), 116.5 (C3), 107.2, 106.1, 105.6 (C3py, C5py, CHSthiazole), 103.1 (C5), 20.9 (CH_3_). HRMS (*m/z*): [MH]^+^ calc for C_31_H_26_N_7_O_2_S_2_, 592.1584; found, 592.1584. HPLC: purity 95%; t_R_ (MeOH 80:20 TFA 0.1%): 6.01 min.

### 4.2 Biological Assays

#### 4.2.1 Cell Screening

RD‐ES cells were cultured in RPMI 1640 medium (Gibco, MD, USA) supplemented with 15% fetal bovine serum (FBS) and 1% penicillin/streptomycin, and maintained in a humidified incubator under a 5% CO₂ atmosphere. Daoy cells were cultured in DMEM‐low medium (Gibco, MD, USA) supplemented with 10% FBS and 1% penicillin/streptomycin, and maintained under the same incubation conditions. Both cell lines were seeded at their respective densities and incubation times in 96‐well plates, as described in Table 4. Compounds were prepared as stock solutions in DMSO, diluted in culture medium to a final concentration of 10 µM, with the DMSO concentration not exceeding 0.2% (v/v). Compounds were added 24 h after seeding, and the plates were further incubated under 5% CO₂ at 37°C for 48 h. Vincristine and AZD5363 were used as positive controls.

**Table 4:** Cell lines and their parameters used in this study.

Cell line	Histological type	Origin	Cultivation medium	Plating concentration (cell/mL)	Incubation time (hours)
RD‐ES	Ewing Sarcoma	Human	RPMI 1640	9×10^5^	48
Daoy	Medulloblastoma	Human	DMEM‐low	4×10^5^	48

After the incubation period, 10 µL of MTT solution (5 mg/mL in PBS) was added to each plate well. Then, once again, they were incubated for 3 h under 5% CO_2_ at 37°C. After this time, the plates were centrifuged (3000 rpm/15 min at 25°C), and the supernatant was discarded. The plate was allowed to settle overnight, and then 100 μL DMSO was added and incubated for 30 min to solubilize the formazan crystals. The optical density was measured in a spectrophotometer at 570 nm [50]. The viability values were obtained using Excel and compared with the untreated cells.

#### 4.2.2 Cell Viability Studies

Cells from the strains used in the study were cultured under optimal conditions and plated at their respective concentrations, as described in Table 4. After 24 h of settling, the compounds, prepared as stock solutions in DMSO were added at concentrations of 50, 35, 20, 10, 5, and 2.5 µM or 7.2, 3.6, 1.8, 0.9, 0.45, and 0.225 µM (final DMSO concentration not exceeding 0.5%—v/v) and the plates were incubated again under 5% CO_2_ at 37°C for 48 h. Then, the MTT assay procedure was carried out as described above. The IC_50_ values and respective mean standard deviations (SD) were calculated by nonlinear regression using the GraphPad Prism and Microsoft Excel.

#### 4.2.3 Kinase Selectivity Panel

The Thermal Shift Assay was performed as previously described [33, 51]. Purified proteins were buffered in 25 mM HEPES (pH 7.5), 500 mM NaCl, and were assayed in a 384‐well plate with a final protein concentration of 2 μM in 10 μL assay volume. Inhibitors were added to a final concentration of 10 μM, using an ECHO 550 acoustic dispenser (Labcyte). As a fluorescence probe, SYPROOrange (Molecular Probes) was added in a 1:5000 dilution. Filters for excitation and emission were set to 465 nm and 590 nm, respectively. The temperature increased from 25°C with 3°C/min to a final temperature of 95°C, while scanning, using the QuantStudio5 (Applied Biosystems). Data were analyzed through Boltzmann‐equation in the Protein Thermal Shift software (Applied Biosystems). Samples were measured in technical duplicates.

#### 4.2.4 Enzymatic Assays

The inhibition assessment and IC_50_ determination assays were conducted using ADP‐Glo assay kit with purified GAK and ATP (100 µM = ~2 x GAK KmATP). First, compounds were tested at 1 µM against GAK to evaluate their capability of inhibition. The enzyme was diluted with reaction buffer (50 mM HEPES, pH 7,5, 10 mM MgCl2, 1 mM EGTA, 0.01% Tween‐20, 2 mM DTT) to a final concentration of 62.5 nM. Then, 4 µL of this solution were distributed into a 384‐well, round‐bottom, white assay plate (Corning, Cat # 4512). Compounds were transferred (100 nL of 50 µM stocks) to the assay plate using an automated liquid handler robot (CyBio Felix, Analytic Jena), and the plate was incubated at 30°C for 30 min before the addition of 1.0 µL of 500 µM Ultra‐pure ATP solution in reaction buffer, and incubation at 30°C for 180 min. The final assay concentrations were 50 nM for GAK and 100 µM for the ATP. Next, to stop the reactions, 5 µL of ADP‐Glo Reagent (ADR, Promega, Cat # V912C) was added, followed by incubation at RT for 60 min. A total of 10 µL of the Kinase Detection Buffer (KDR, Promega, Cat # V917A) was then added, followed by a 60 min incubation at RT. The resulting luminescence was measured on a CLARIOstar (BMG Labtech) plate reader with an emission filter of 545–550 nm.

To estimate half‐maximum inhibitory concentration (IC_50_) for the tested compounds, a 14‐point serial dilution (twofold dilution) from a 10 mM stock was prepared in 100% DMSO. Reaction preparation, compound transfer, and detection steps were carried out as described above. IC_50_ values were obtained by fitting the data to a four‐parameter dose‐response equation using Prism (Graphpad Software, MA, USA).

### 4.3 In Silico Study

#### 4.3.1 Protein and Ligand Preparation

The X‐ray crystal structure of GAK complexed with Gefitinib was obtained from the RCSB Protein Data Bank (PDB ID: 5Y7Z). GOLD program 2022.1.0 (Cambridge Crystallographic Data Centre (CCDC), Cambridge, UK) was used to prepare the protein by removing all waters and ligands and adding hydrogens [52, 53]. MarvinSketch 21.9.0 program and the Webserver NovoPro were used for drawing the 2D and 3D structures of the ligands, respectively [54, 55]. The ligands were optimized using Avogadro 1.2.0 employing the Universal Force Field [56].

#### 4.3.2 Molecular Docking

*D*ocking studies were performed using GOLD. Ligands were considered flexible and docked in the binding site of the protein using the grid box centered at the crystallographic ligand. The default calculation mode was selected for all calculations without selecting early termination. Software validation was verified by the root‐mean‐square deviation (RMSD) between the experimental and predicted ligand poses of the original crystallographic ligand, in this case Gefitinib. An RMSD value < 2 Å suggests a correct docked pose prediction [57, 58, 59]. Discovery Studio Visualizer 2025 was used to analyze docking results and prepare figures of ligand binding poses [45, 60].

## Conflicts of Interest

The authors declare no conflicts of interest.

## Supporting information

Supporting Information clean.

Supporting_Publishing_InChI.
