# Supplementary material for: New Quinoline Kinase Inhibitors With Good Selectivity for NAK Kinases and Anti‐Tumor Activity Against Ewing Sarcoma
Source: Arch Pharm (Weinheim). 2026 Jan 10;359(1):e70184. doi: 10.1002/ardp.70184 (PMC12790329; doi:10.1002/ardp.70184)
Supplement: Supplementary file 1 — Supporting Information clean. [file ARDP-359-e70184-s002.docx]

**Supporting Information**

New quinoline kinase inhibitors with good selectivity for NAK kinases and anti-tumor activity against Ewing Sarcoma

Caroline de Bem Gentz^1,2^, Thais Helena Maciel Fernandes^1,2^, Marcela Silva Lopes^3,4^, Lewis Elson^5^, Andreas Krämer^5^, Lucas Rodrigo de Souza^6^, Isadora Serraglio Fortes^1,2,4^, Geórgia Silva Pinto^2^, Martha Cestari Silva Martins^1,2^, Henrique Barros de Lima^1,2^, André da Silva Santiago^6^, Lauro José Gregianin^4,7,8,9^, Katlin Brauer Massirer^6^, Mário Henrique Bengtson^6^, Rafael Roesler^3,4,7^, Stefan Knapp^5^, Stefan A. Laufer^10*^, Saulo Fernandes de Andrade^1,2,4*^

1 Pharmaceutical Sciences Graduate Program, Universidade Federal do Rio Grande do Sul (UFRGS), Porto Alegre, RS, Brazil;

2 Pharmaceutical Synthesis Group (PHARSG), School of Pharmacy, Universidade Federal do Rio Grande do Sul (UFRGS), Porto Alegre, RS, Brazil;

3 Department of Pharmacology, Institute of Basic Health Sciences, Universidade Federal do Rio Grande do Sul (UFRGS), Porto Alegre, RS, Brazil;

4 National Science and Technology Institute for Children’s Cancer Biology and Pediatric Oncology - INCT BioOncoPed, Porto Alegre, Brazil;

5 Institute of Pharmaceutical Chemistry, Goethe University, Frankfurt am Main, Germany;

6 Center for Medicinal Chemistry (CQMED), Center for Molecular Biology and Genetic Engineering (CBMEG), University of Campinas (UNICAMP), Campinas, SP, Brazil;

7 Cancer and Neurobiology Laboratory, Experimental Research Center, Clinical Hospital (CPE-HCPA), Universidade Federal do Rio Grande do Sul, Porto Alegre, RS, Brazil;

8 Department of Pediatrics, School of Medicine, Universidade Federal do Rio Grande do Sul, Porto Alegre, RS, Brazil;

9 Pediatric Oncology Service, Clinical Hospital, Universidade Federal do Rio Grande do Sul, Porto Alegre, RS, Brazil;

10 Department of Pharmaceutical and Medicinal Chemistry, Institute of Pharmaceutical Sciences, University of Tübingen, Tübingen, Germany.

*Correspondence:

Prof, Saulo Fernandes de Andrade, Pharmaceutical Sciences Graduate Program, Universidade Federal do Rio Grande do Sul, Porto Alegre, Rio Grande do Sul, Brazil. 90010-150. Pharmaceutical Synthesis Group (PHARSG), School of Pharmacy, Universidade Federal do Rio Grande do Sul (UFRGS), Porto Alegre, RS, Brazil; National Science and Technology Institute for Children’s Cancer Biology and Pediatric Oncology - INCT BioOncoPed, Porto Alegre, Brazil.

Email: saulo.fernandes@ufrgs.br

Prof, Stefan A. Laufer - Department of Pharmaceutical and Medicinal Chemistry, Institute of Pharmaceutical Sciences, University of Tübingen, Tübingen, 72074, Germany.

Email: stefan.laufer@uni-tuebingen.de

**Table S1.** DSF assay against a panel of 95 kinases with the corresponding T_m_-shifts of compounds **3a**, **3c**, **11a**, **11d**, **11e** and references.

| **Kinase** | **Compound ΔT_m_ °C** | | | | | | **Reference compound** |
| --- | --- | --- | --- | --- | --- | --- | --- |
|  | **11e** | **11a** | **3c** | **3a** | **11d** | **Reference T_m_ shift** |  |
| AAK1 | 4,1 | 4,1 | 6,1 | 5,6 | 2,1 | 15,3 | Staurosporine |
| ABL1 | 2,4 | 1,3 | 0 | 1,8 | 1,1 | 8,6 | Staurosporine |
| AKT3 | 0,3 | 0,4 | 0,2 | 0,3 | 1,1 | 7 | Staurosporine |
| AURKB | 3,2 | 2,2 | 0,7 | 2,1 | 3 | 8 | Staurosporine |
| BMP2K | 5,1 | 4,4 | 7,8 | 7,6 | 2,4 | 18,4 | Staurosporine |
| BMX | 0,4 | 0,5 | 0,1 | 1,6 | 1,3 | 6,8 | Staurosporine |
| BRAF | 0,9 | 0,8 | -0,2 | 2,7 | 0,4 | 26,6 | Dabrafenib |
| BRD4 | 0,4 | 2,4 | 0,4 | 1,2 | 3,3 | 7,0 | JQ1 |
| BRPF1 | -1,9 | 2,9 | -1,5 | 0,6 | 1,5 | 14,0 | GSK6853 |
| CAMK1D | 0,8 | 2,1 | 0 | 0,6 | 2,6 | 8,9 | Staurosporine |
| CAMK2B | 0,5 | 0,4 | 1 | 0,6 | 0,5 | 8,8 | Staurosporine |
| CAMK2D | 2,2 | 2,9 | 2,2 | 2,4 | 3,1 | 15,8 | Staurosporine |
| CAMK4 | 1,1 | 0,9 | 0,9 | 2,1 | 2,2 | 8,2 | Staurosporine |
| CAMKK2 | 1,9 | 0,9 | 0,4 | 2,2 | 1 | 23,5 | Staurosporine |
| CASK | 0,4 | -0,2 | 0,3 | -0,2 | 0,2 | 5,2 | Staurosporine |
| CDK2 | 1 | 0,2 | 0,7 | 0,3 | 0,9 | 15,2 | Staurosporine |
| CDKL1 | 0,4 | 0,3 | 0,2 | 0,2 | 1,4 | 6,6 | CEP-32496 |
| CHEK2 | 4,6 | 2,2 | 3,7 | 3,5 | 1,8 | 17,1 | Staurosporine |
| CLK1 | 2,2 | 2,6 | 5,3 | 4,8 | 3,3 | 15,7 | Staurosporine |
| CLK3 | 1,3 | 1,5 | 1,2 | 2,6 | 1,1 | 15,0 | CLK-T3 |
| CSNK1D | 0,8 | 0,2 | 0,1 | 0,4 | -0,2 | 9,0 | PF-670462 |
| CSNK1E | 1,4 | 1,4 | 0,7 | 1,4 | 0,3 | 8,0 | PF-670462 |
| CSNK2A2 | 0,7 | -0,6 | -0,1 | 2 | 2,7 | 15,7 | Similtasertib |
| DAPK1 | 2,8 | 1,3 | 2,3 | 1,7 | 1,1 | 9,0 | Staurosporine |
| DAPK3 | 5,4 | 3,5 | 6,5 | 5,9 | 6,6 | 16,2 | Staurosporine |
| DCAMKL1 | 2,7 | 1,9 | 3,9 | 3,1 | 4,8 | 11,4 | Staurosporine |
| DMPK1 | 1 | 0,8 | 0,6 | 0,5 | 0,5 | 9,3 | Staurosporine |
| DYRK1A | -0,6 | 0,2 | -0,6 | 0,2 | 1,4 | 9,9 | Staurosporine |
| DYRK2 | 1,1 | 1,2 | 1,9 | 3,7 | 0,7 | 7,3 | Staurosporine |
| EPHA2 | 0,4 | 0,8 | 0,4 | 0,8 | 0,7 | 7,1 | Staurosporine |
| EPHA4 | 0,5 | 1,1 | 0,2 | 0,3 | 0,7 | 5,3 | Staurosporine |
| EPHA5 | 1,3 | 2,3 | 0,7 | 1,9 | 2,1 | 6,9 | Staurosporine |
| EPHA7 | 1,9 | 2,6 | 0,6 | 1,8 | 3,6 | 10,5 | Staurosporine |
| EPHB1 | 0,3 | 0,5 | -0,1 | 0,1 | 0,9 | 6,4 | Staurosporine |
| EPHB3 | 0,6 | 1,5 | 0,4 | 1,1 | 3,6 | 6,1 | Staurosporine |
| FES | 0,9 | 2 | 1,6 | 2 | 1,5 | 7,5 | Staurosporine |
| FGFR1 | 0,1 | 1,2 | 0,2 | 0,4 | 3 | 5,7 | Staurosporine |
| FGFR2 | 0,4 | 1,3 | 0,8 | 0,6 | 3 | 8,3 | Staurosporine |
| GAK | 5,1 | 5,5 | 6,6 | 4,8 | 5,8 | 8,8 | Staurosporine |
| GPRK5 | 0,3 | 0,7 | 0,4 | 0,9 | 0,7 | 5,9 | Staurosporine |
| GSG2 | 1,3 | 1,3 | 1,7 | 3,9 | 4,7 | 8,9 | Staurosporine |
| GSK3B | 3,7 | 1,3 | 1,2 | 2,5 | 0,8 | 9,0 | Staurosporine |
| HIPK2 | 0,7 | 0,7 | 0,3 | 2,1 | 1 | 11,6 | GW779439X |
| MAP2K1 | 0,3 | 0,2 | 0,2 | 0,6 | 0,7 | 3,2 | Staurosporine |
| MAP2K4 | 1,5 | 1,6 | 1,8 | 3,6 | 1,5 | 10,5 | Staurosporine |
| MAP2K6 | 1,5 | 0,6 | 0,8 | 1,1 | 0,9 | 11,3 | Staurosporine |
| MAP2K7 | -0,4 | -0,3 | 2,5 | 3,3 | 3,7 | 7,0 | Staurosporine |
| MAP3K5 | 0,6 | 0,6 | 0,3 | 1,7 | 1,5 | 15,5 | Staurosporine |
| MAPK1 | 4 | 4,6 | 1,8 | 4,1 | 2,6 | 7,5 | GDC-0994 |
| MAPK10 | 1,5 | -0,2 | 0,2 | -0,4 | -0,5 | 8,8 | CEP-32496 |
| MAPK13 | 1,5 | 4,8 | 1,1 | 2,2 | 2,6 | 18,8 | Doramapimod |
| MAPK14 | 4,9 | 0,8 | 1,1 | 1,5 | 0,4 | 19,8 | Doramapimod |
| MAPK15 | 0,9 | 0,6 | 0,6 | -0,1 | 1,5 | 14,0 | Staurosporine |
| MAPK8 | 2,4 | 2,8 | 2,7 | 3,4 | 1,7 | 7,7 | Staurosporine |
| MAPK9 | 2,4 | 2,3 | -0,9 | 1 | 1,2 | 8,6 | SBI-0069279 |
| MAPKAPK2 | 0,4 | 0,3 | 0,5 | 0,4 | 1,5 | 4,0 | Staurosporine |
| MARK3 | -0,3 | 0 | -1 | -0,2 | 2,2 | 18,0 | Staurosporine |
| MARK4 | 1,5 | 1,9 | 0,5 | 1,9 | 2,7 | 14,3 | Staurosporine |
| MELK | 1,3 | 0,5 | 1,1 | 2 | 0,5 | 13,2 | Staurosporine |
| MSSK1 | 0,2 | 0,4 | 0 | 0 | 0,5 | 5,5 | Staurosporine |
| MST4 | 0,6 | 0,8 | -0,1 | 0,8 | 2,4 | 9,8 | RN168 |
| NEK2 | 0,3 | 0,5 | 0,1 | -0,3 | 0,4 | 6,0 | Staurosporine |
| OSR1 | 0,7 | 1,1 | 0,7 | 1,3 | 2,8 | 5,7 | Staurosporine |
| PAK1 | -0,1 | 2,5 | -0,2 | 1,3 | 2,1 | 7,3 | Staurosporine |
| PAK4 | 4,1 | 2,6 | 1,7 | 3 | 2,6 | 12,2 | Staurosporine |
| PCTK1 | 3,3 | 0,7 | 0,4 | 0,5 | 0,7 | 9,0 | Staurosporine |
| PIM1 | 0,9 | 1,5 | 0,8 | 1,7 | 1,7 | 11,9 | Staurosporine |
| PIM3 | 2,3 | 2,6 | 3 | 4,7 | 3,4 | 19,0 | Staurosporine |
| PKMYT1 | 0 | 1,3 | -0,1 | 0,5 | 1,2 | 4,5 | Dasatinib |
| PLK4 | 5,3 | 2,6 | 2 | 2,5 | 1,7 | 18,0 | Staurosporine |
| RPS6KA1 | 0,8 | 1,2 | 0,6 | 1,7 | 1,3 | 3,2 | Staurosporine |
| RPS6KA5 | 2,6 | 3,5 | 1,7 | 4,3 | 2,7 | 15,6 | Staurosporine |
| SLK | 3,5 | 3,8 | 1,5 | 3,4 | 2,7 | 16,9 | Staurosporine |
| SRC | 2 | 0,4 | 0,7 | 1,1 | 0,1 | 5,0 | Staurosporine |
| SRPK1 | 0,3 | 0,1 | 0 | -0,1 | 0,6 | 7,0 | Staurosporine |
| STK10 | 5,3 | 5 | 1 | 3,8 | 3,9 | 23,5 | Staurosporine |
| STK17A | 3,4 | 6,9 | 1,4 | 2,2 | 1,5 | 7,7 | Staurosporine |
| STK17B | 4,1 | 3,9 | 3 | 4,8 | 6,3 | 10,8 | Staurosporine |
| STK3 | 0,3 | 0,4 | 0,3 | 0,3 | 1,2 | 13,4 | Staurosporine |
| STK39 | 0,9 | 0,9 | 0,6 | 1,8 | 1,6 | 11,4 | Staurosporine |
| STK4 | 1 | 2,2 | 0,3 | 1,7 | 1,1 | 15,5 | Staurosporine |
| STK6 | 5,4 | 2,6 | 1,1 | 4 | 4,7 | 16,5 | Staurosporine |
| TAF1 | -0,5 | 0,3 | -0,4 | -0,4 | 1,2 | 7,4 | Bromosporine |
| TIF1 | 0,2 | 0,6 | -0,2 | 0,4 | 1,6 | 6,0 | compound 34 |
| TLK1 | 0,9 | 1 | 0,5 | 0,5 | 2,6 | 8,9 | Staurosporine |
| TTK | 3,4 | 4,2 | 2,6 | 3,1 | 3 | 9,0 | Staurosporine |
| ULK1 | 0,4 | 0,5 | 0,2 | 3,2 | 1,2 | 12,0 | Staurosporine |
| ULK3 | 6,7 | 3 | 2,1 | 3,1 | 1,3 | 17,3 | Staurosporine |
| VRK1 | 0,4 | 1 | 0,3 | 0,6 | 0,7 | 4,5 | MR1 |
| 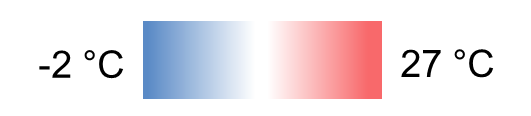 | | | | | | | |


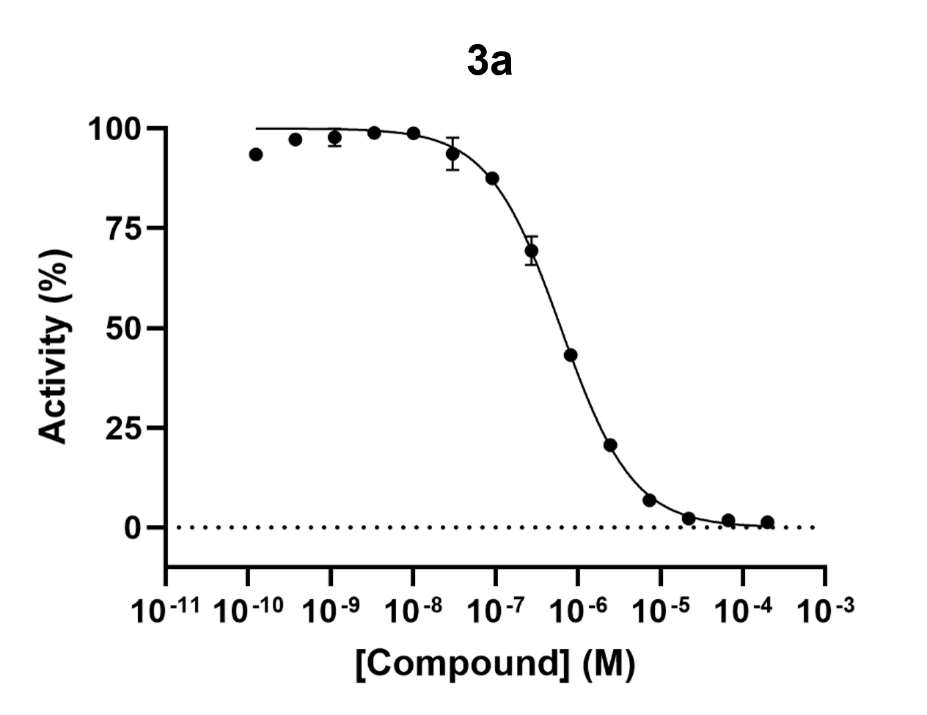


**Figure S1**. Dose-response curves for GAK activity in presence of compound **3a**.


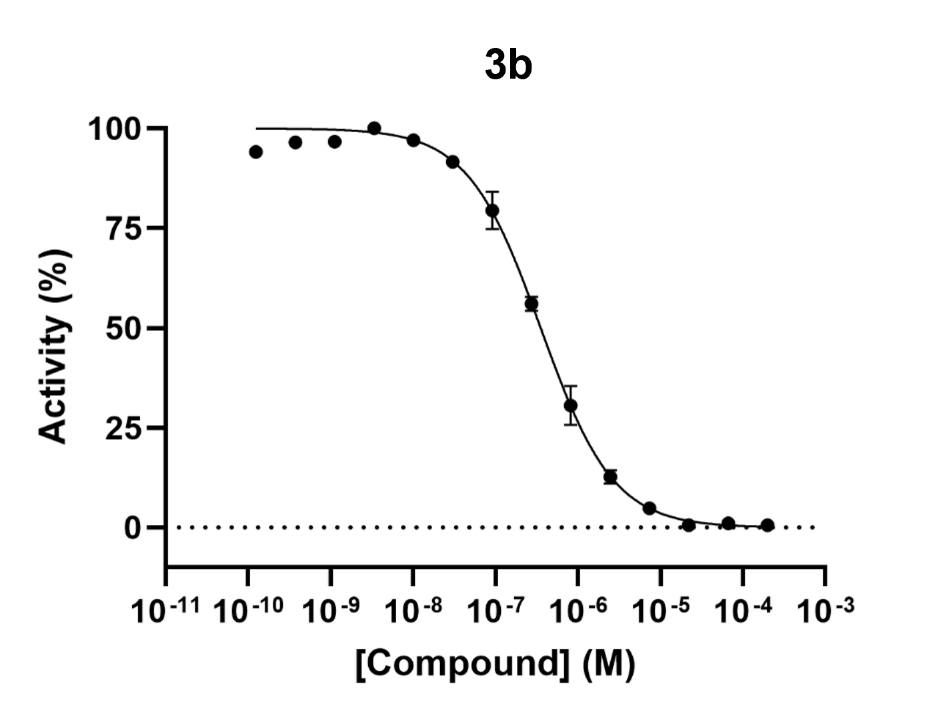


**Figure S2**. Dose-response curves for GAK activity in presence of compound **3b**.


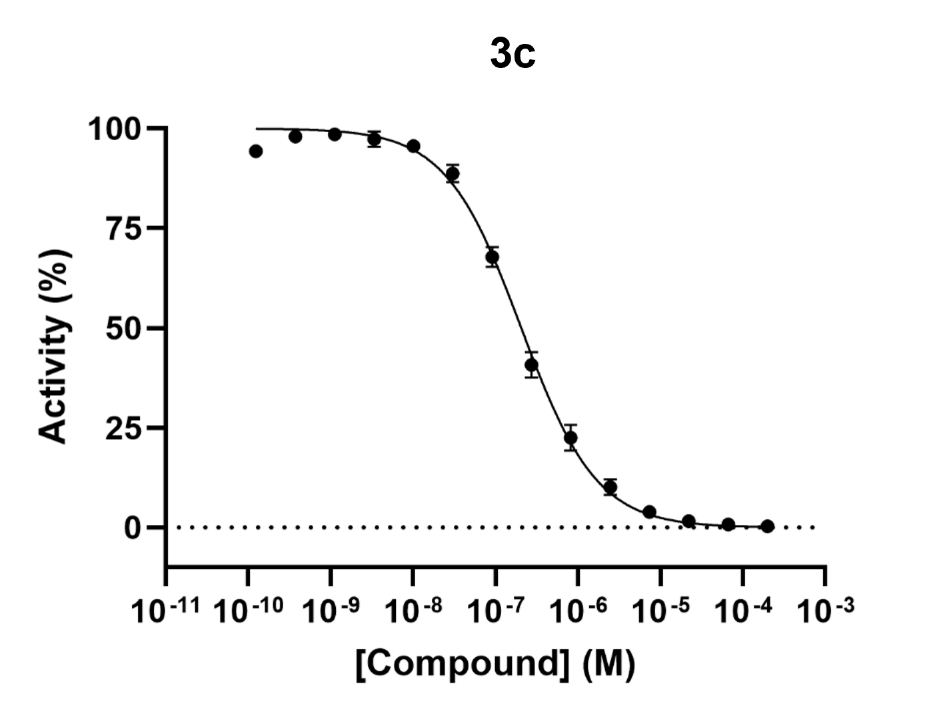


**Figure S3**. Dose-response curves for GAK activity in presence of compound **3c**.


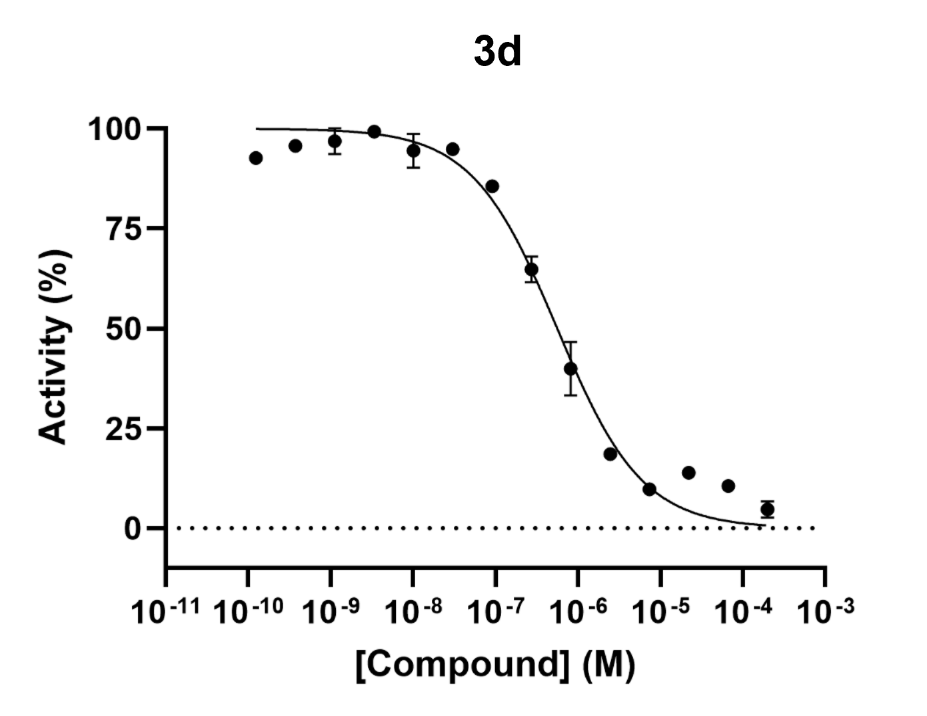


**Figure S4**. Dose-response curves for GAK activity in presence of compound **3d**.


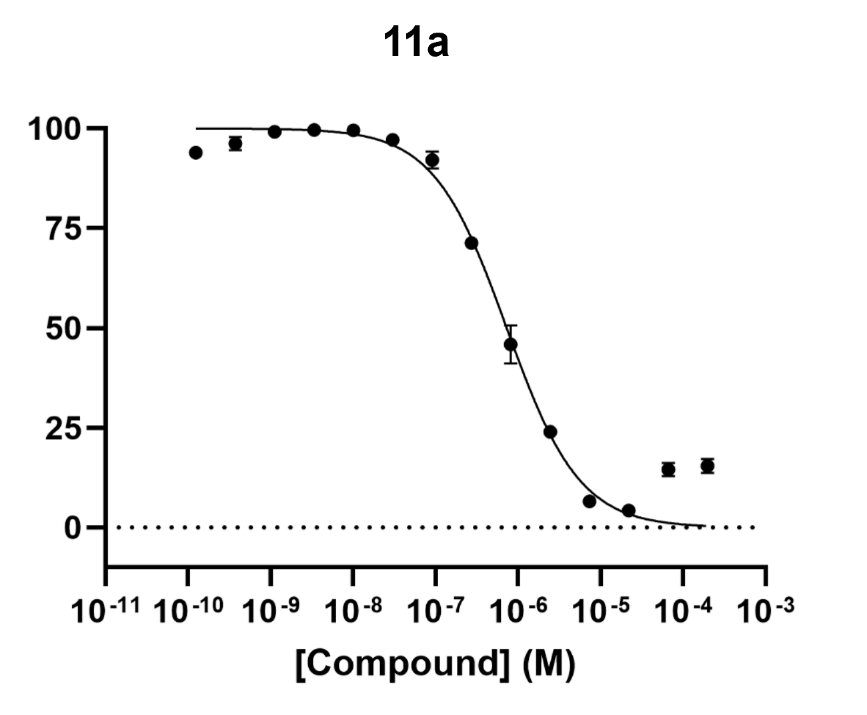


**Figure S5**. Dose-response curves for GAK activity in presence of compound **11a**.


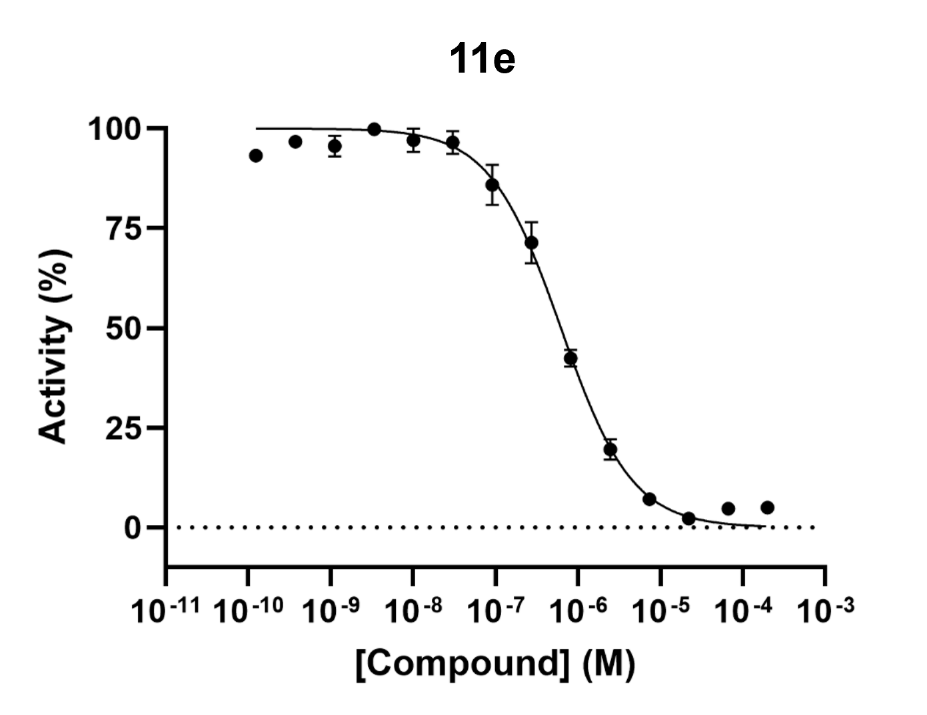


**Figure S6**. Dose-response curves for GAK activity in presence of compound **11e**.

**
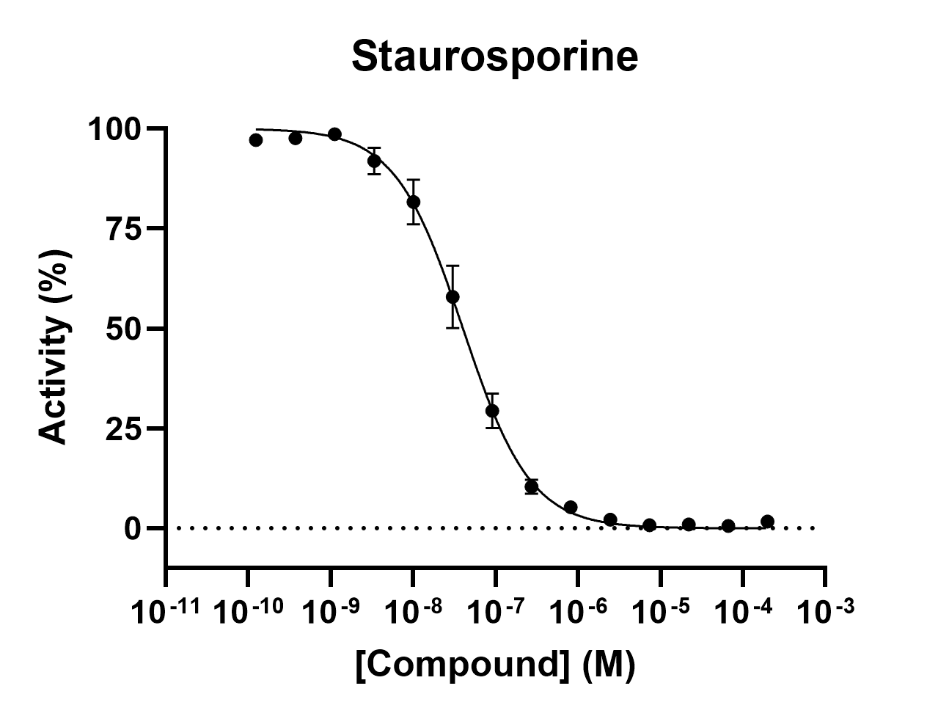
**

**Figure S7**. Dose-response curves for GAK activity in presence of **Staurosporine**.


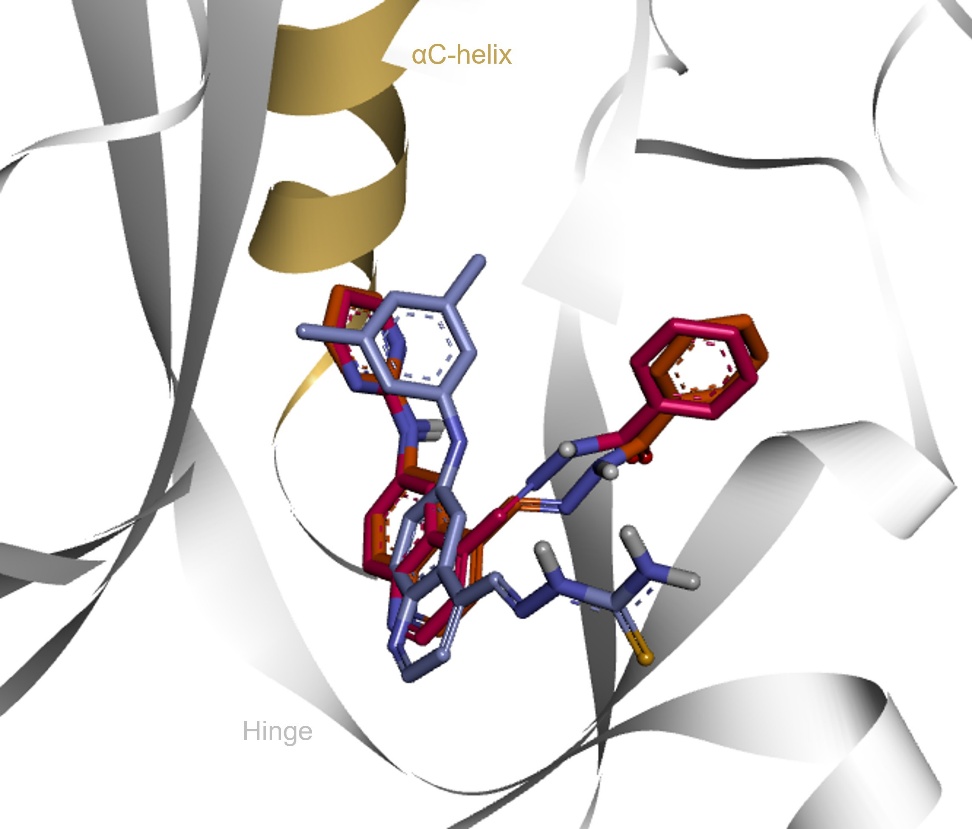


**Figure S8**. Docked poses of **3c** (blue), **11a** (orange), and **11e** (pink) in the active site of GAK (PDB: 5Y7Z). Comparison of **3c** (thiosemicarbazone) with **11a** and **11e** (hydrazone). This figure was prepared using BIOVIA Discovery Studio Visualizer 2025^[1]^.

**NMR data**


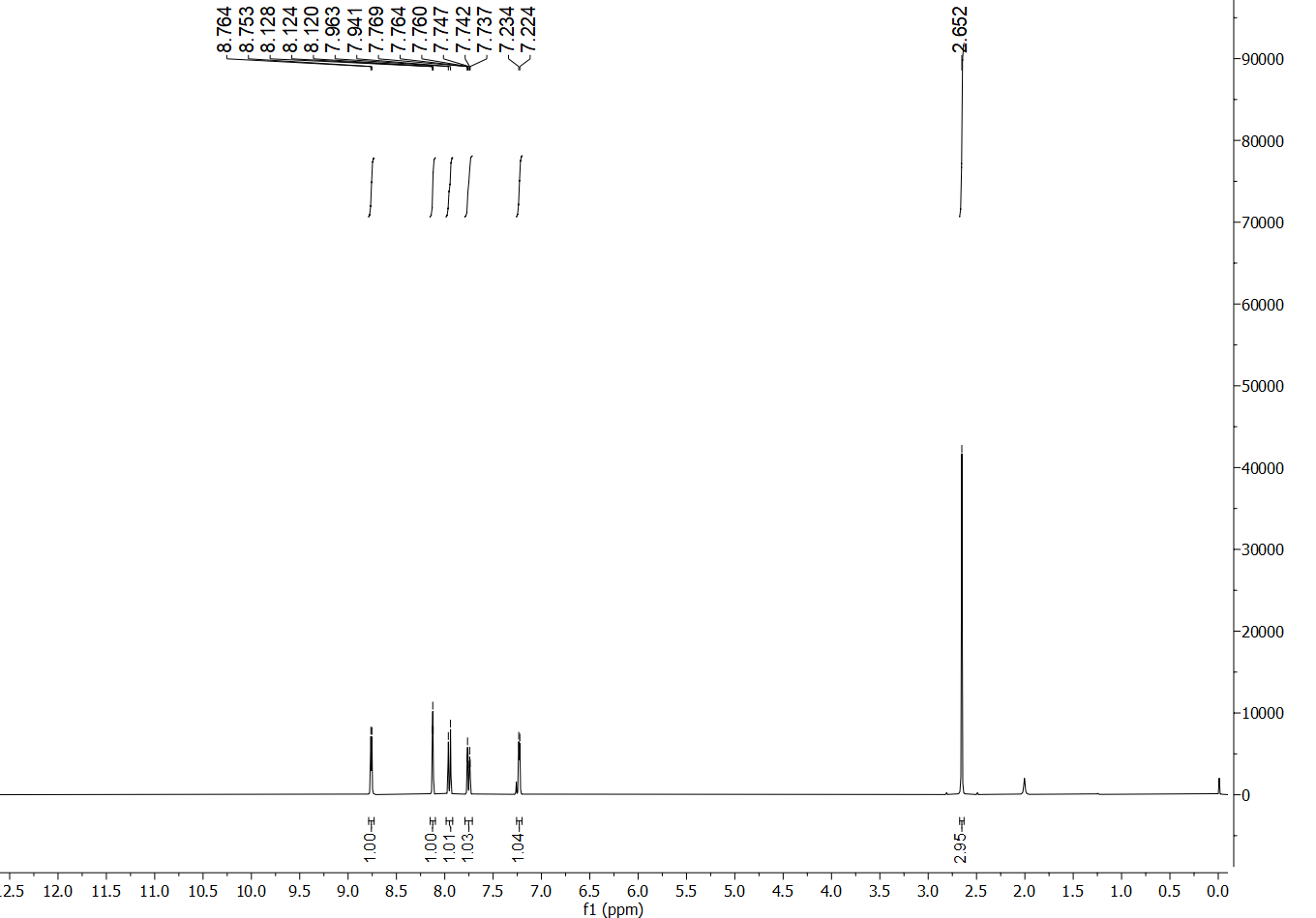


**Figure S9.** ^1^H NMR of 6-bromo-4-methylquinoline (**6**) (CDCl_3_, 400 MHz).


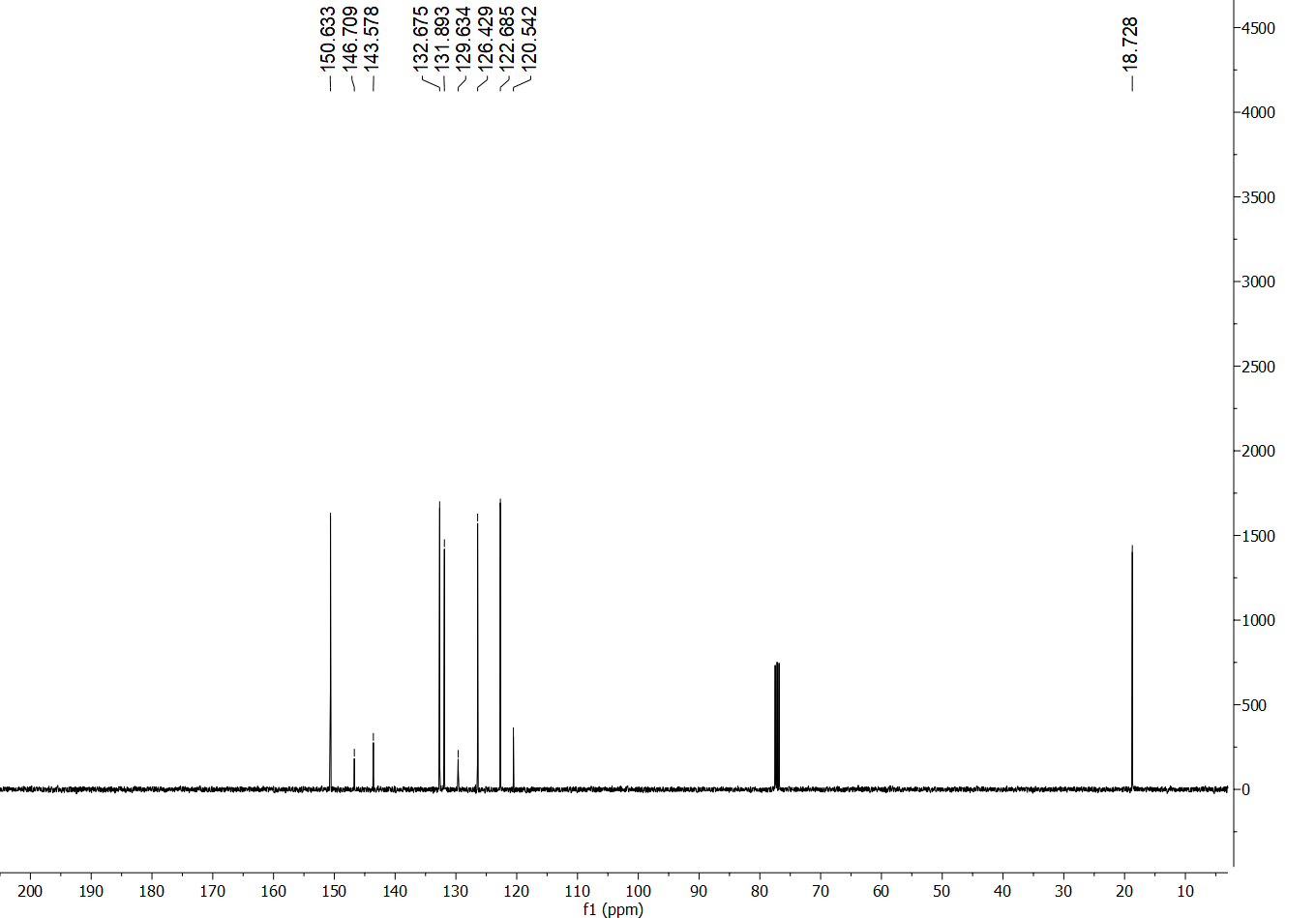


**Figure S10.** ^13^C NMR of 6-bromo-4-methylquinoline (**6**) (CDCl_3_, 100 MHz).


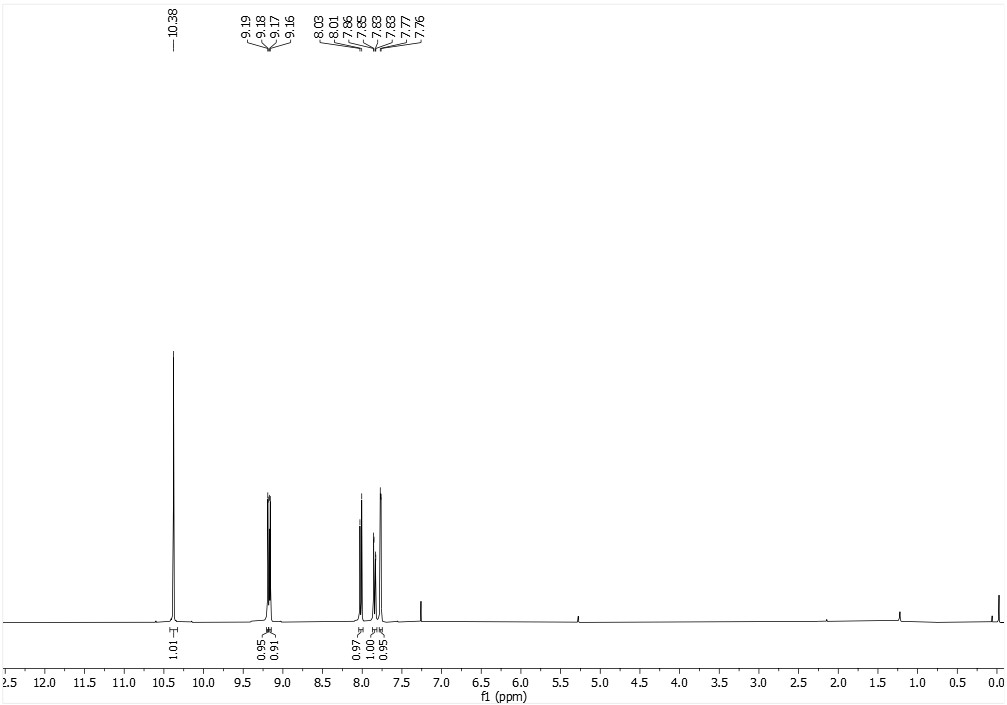


**Figure S11.** ^1^H NMR of 6-bromoquinoline-4-carbaldehyde (**7**) (CDCl_3_, 400 MHz).


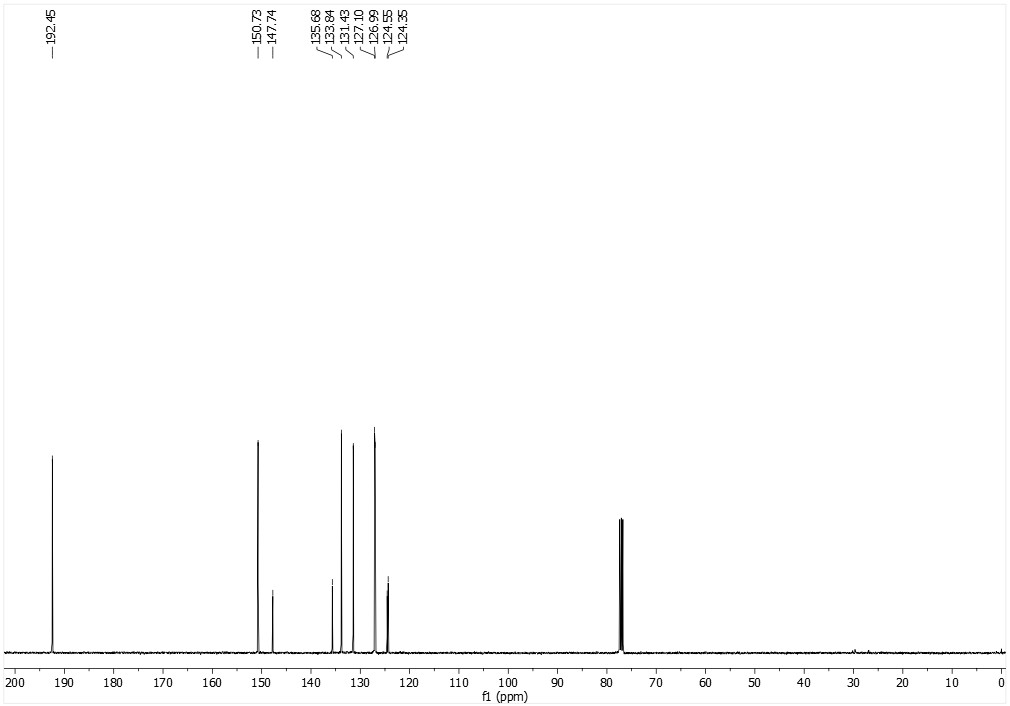


**Figure S12.** ^13^C NMR of 6-bromoquinoline-4-carbaldehyde (**7**) (CDCl_3_, 100 MHz).


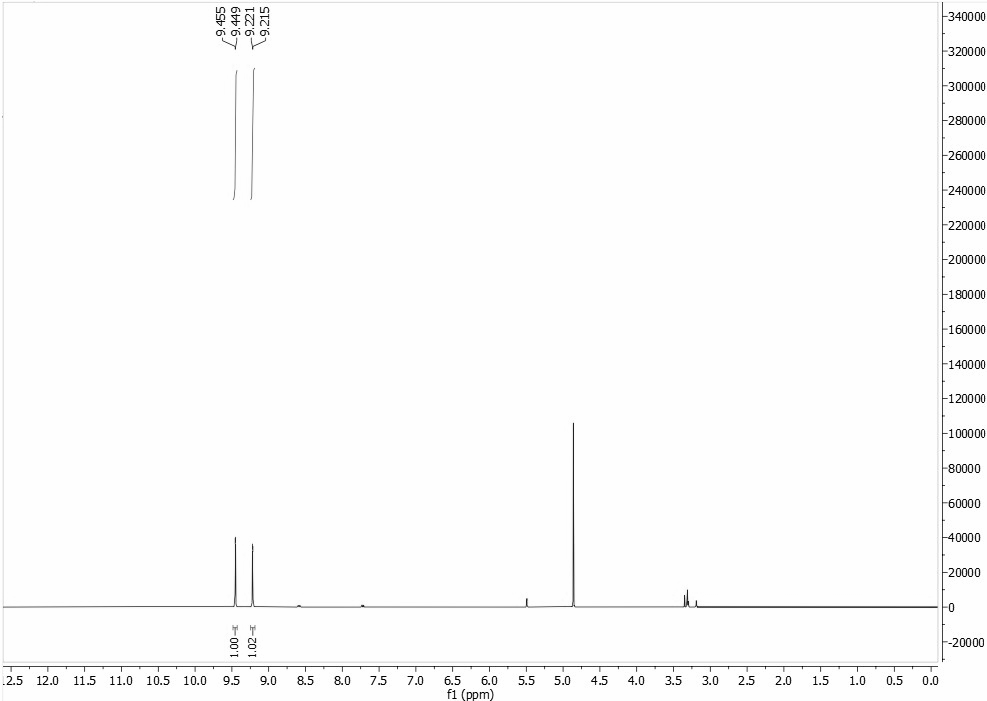


**Figure S13.** ^1^H NMR of 2-chloro-3,5-dinitropyridine (**14**) (MeOD, 400 MHz).


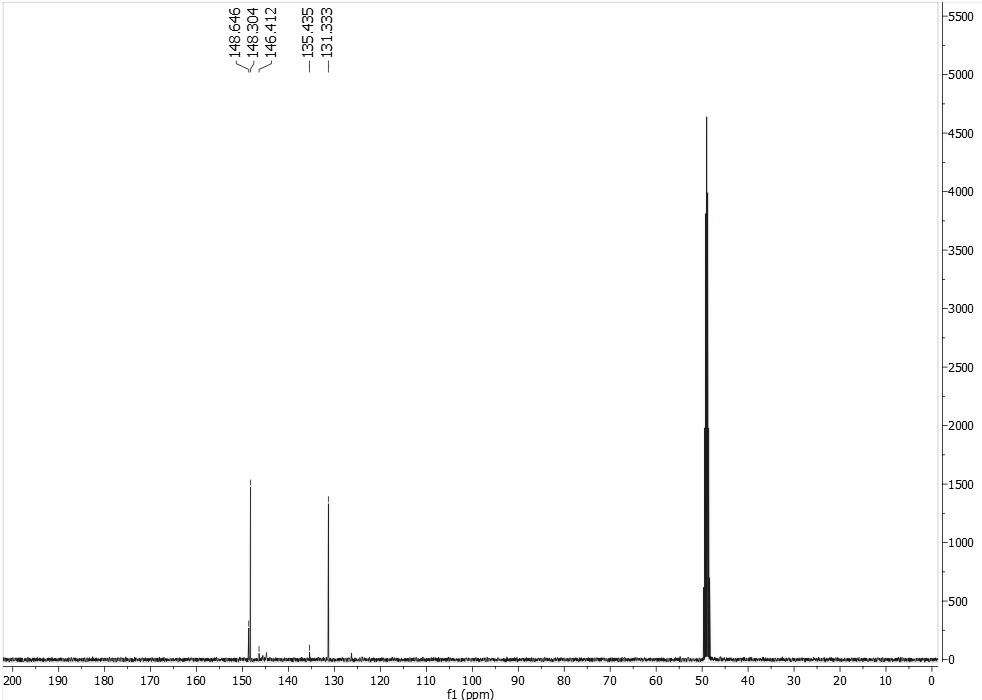


**Figure S14.** ^13^C NMR of 2-chloro-3,5-dinitropyridine (**14**) (MeOD, 100 MHz).


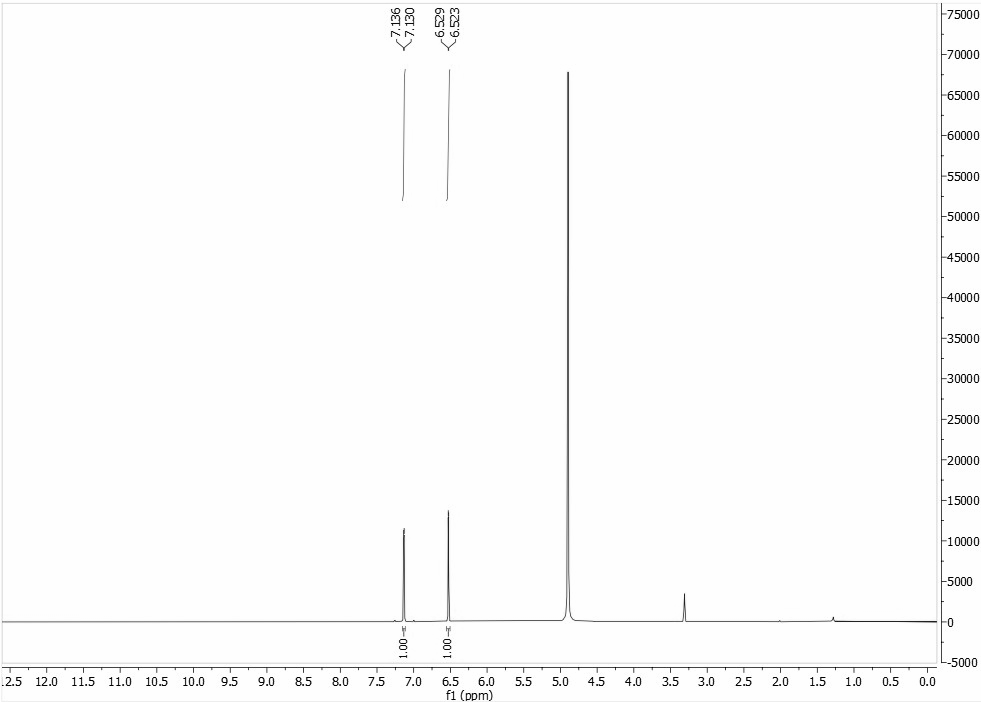


**Figure S15.** ^1^H NMR of 2-chloropyridine-3,5-diamine (**15**) (MeOD, 400 MHz).


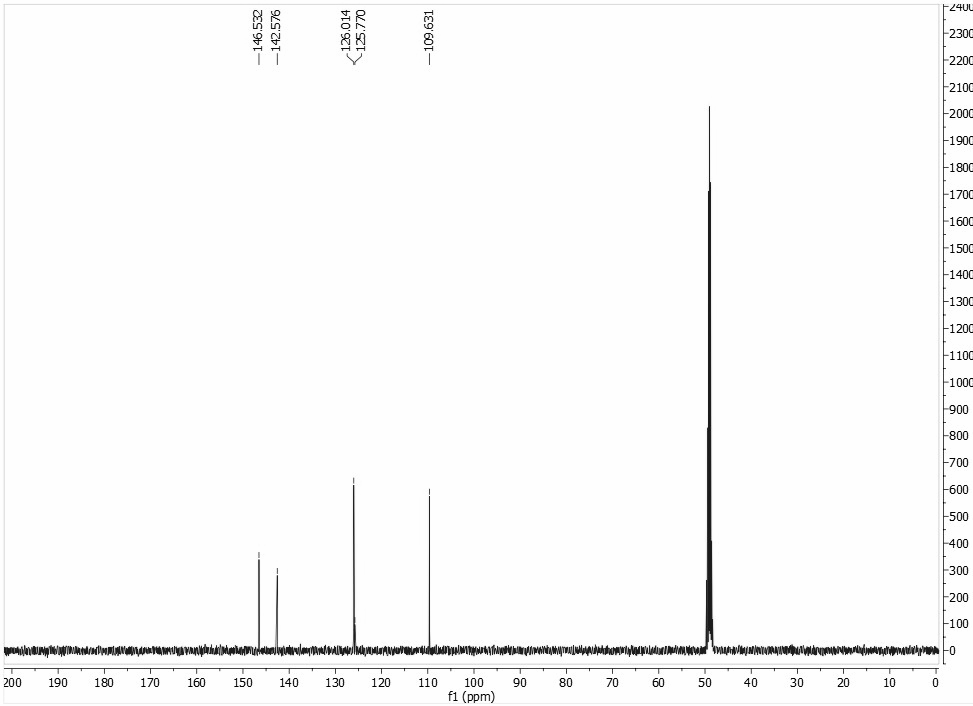


**Figure S16.** ^13^C NMR of 2-chloropyridine-3,5-diamine (**15**) (MeOD, 100 MHz).


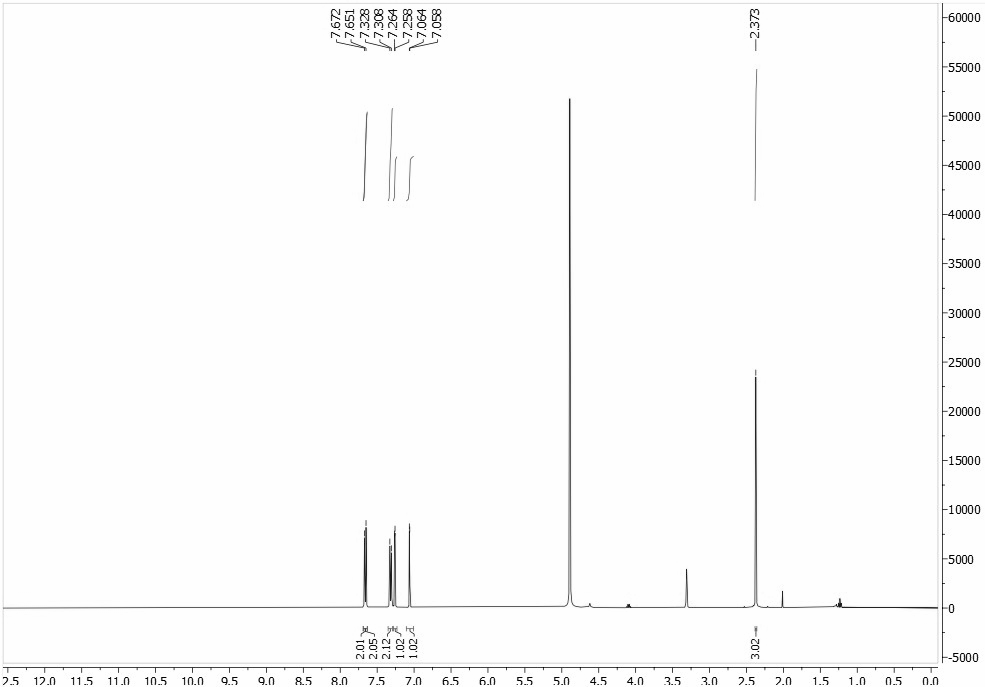


**Figure S17.** ^1^H NMR of *N*-(5-amino-6-chloropyridin-3-yl)-4-methylbenzenesulfonamide (**16**) (MeOD, 400 MHz).


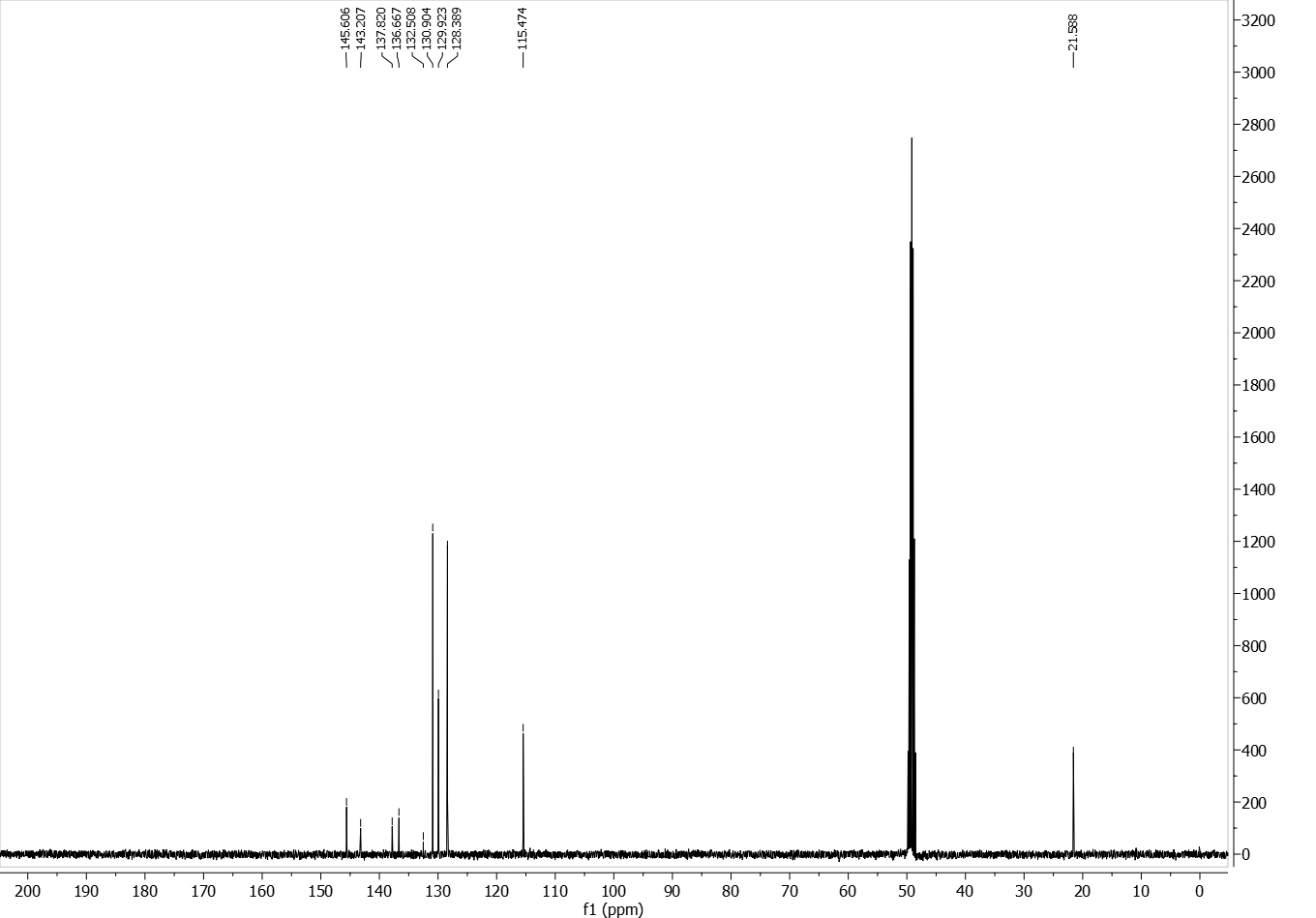


**Figure S18.** ^13^C NMR of *N*-(5-amino-6-chloropyridin-3-yl)-4-methylbenzenesulfonamide (**16**) (MeOD, 100 MHz).

**
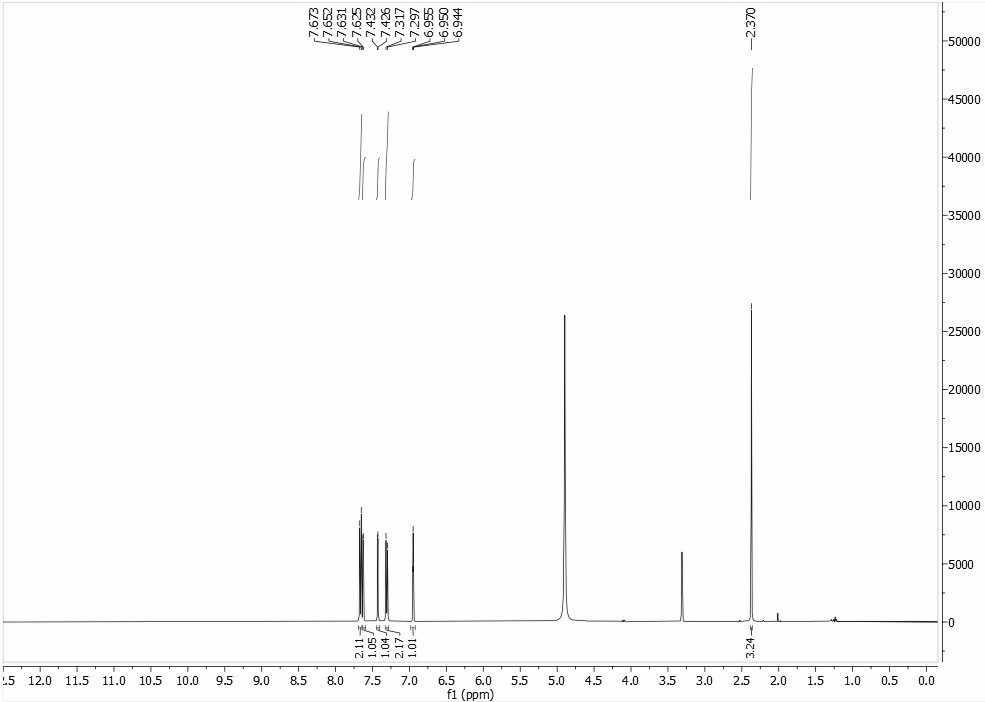
**

**Figure S19.** ^1^H NMR of *N*-(6-aminopyridin-2-yl)-4-methylbenzenesulfonamide (**18**) (MeOD, 400 MHz).


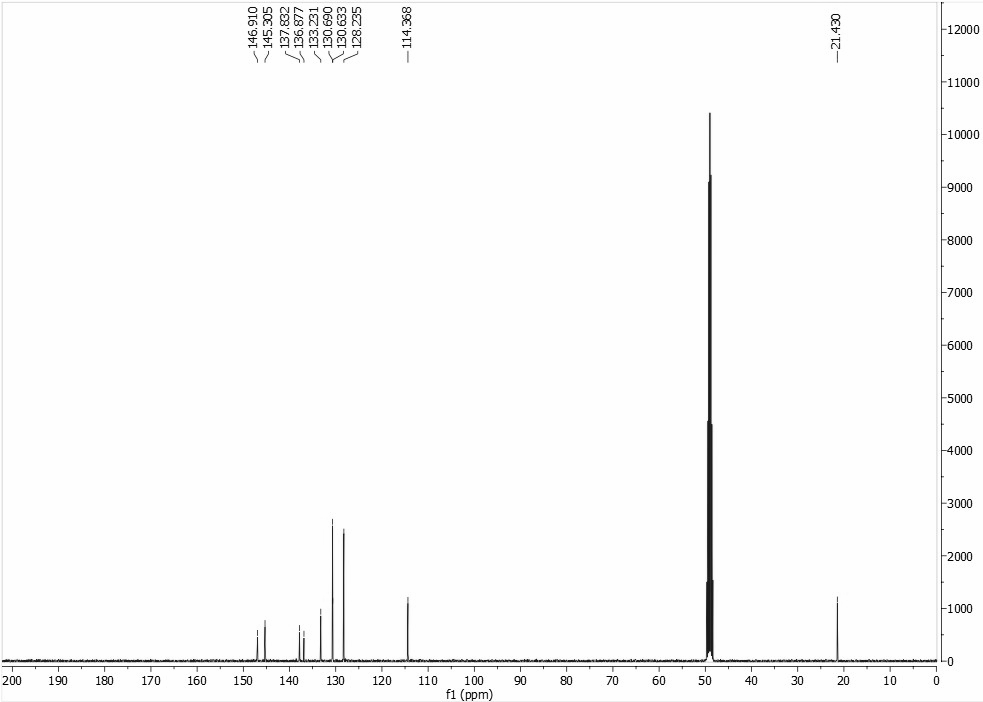


**Figure S20.** ^13^C NMR of *N*-(6-aminopyridin-2-yl)-4-methylbenzenesulfonamide (**18**) (MeOD, 100 MHz).


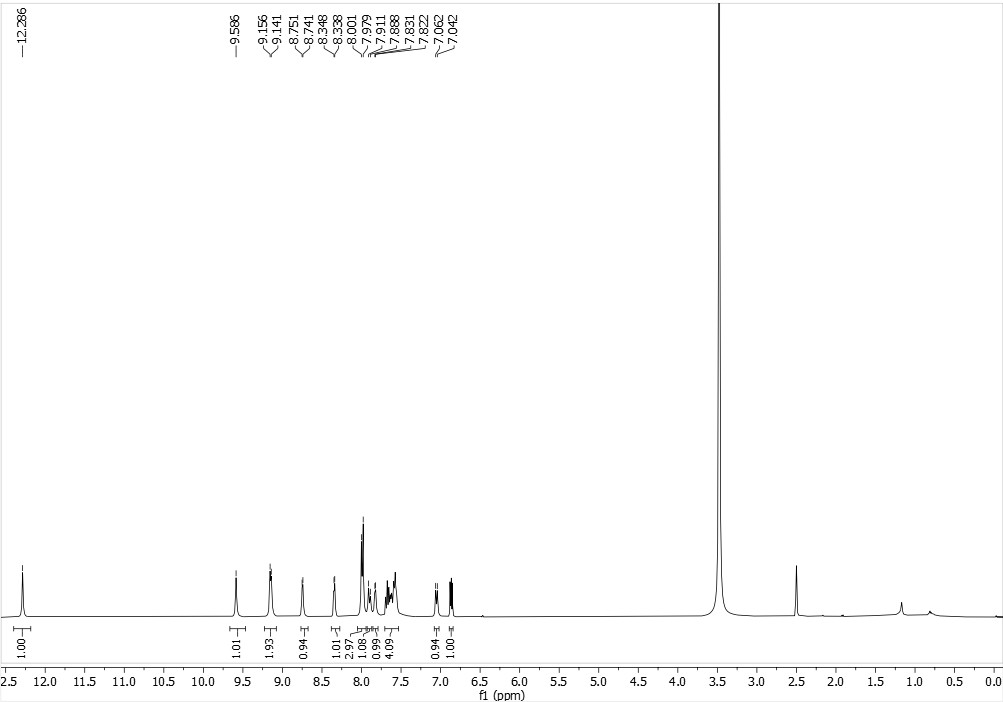


**Figure S21.** ^1^H NMR of *(E)*-*N*'-((6-(pyridin-2-ylamino)quinolin-4-yl)methylene)benzohydrazide (**11a**) (DMSO-*d*_6_, 400 MHz)_._

_
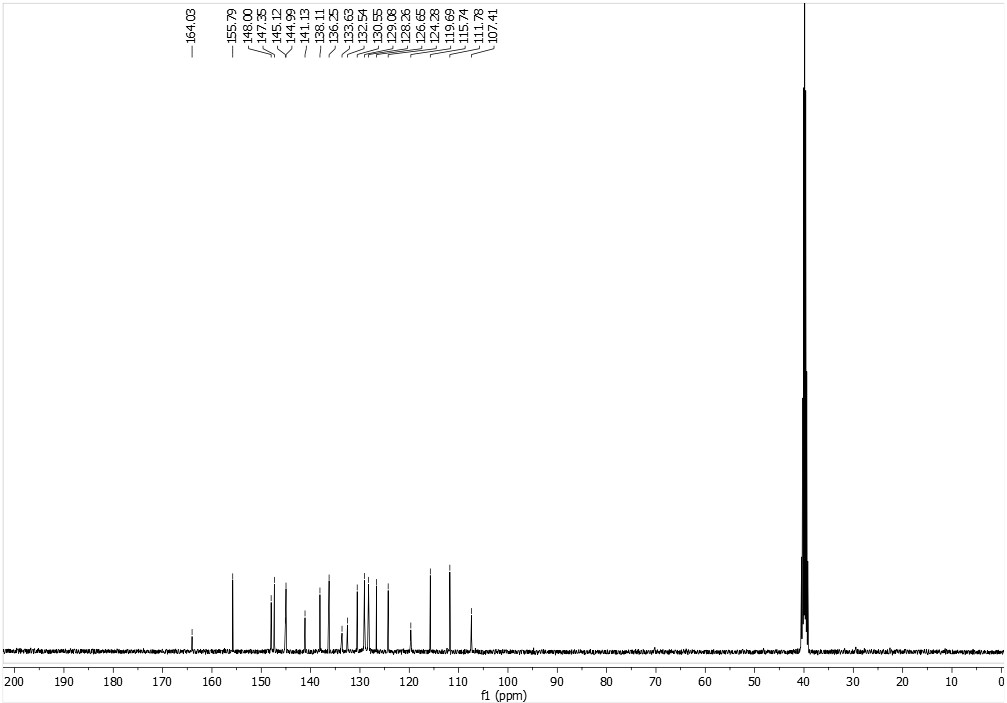
_

**Figure S22.** ^13^C NMR of *(E)*-*N*'-((6-(pyridin-2-ylamino)quinolin-4-yl)methylene)benzohydrazide (**11a**) (DMSO-*d*_6_, 100 MHz)_._


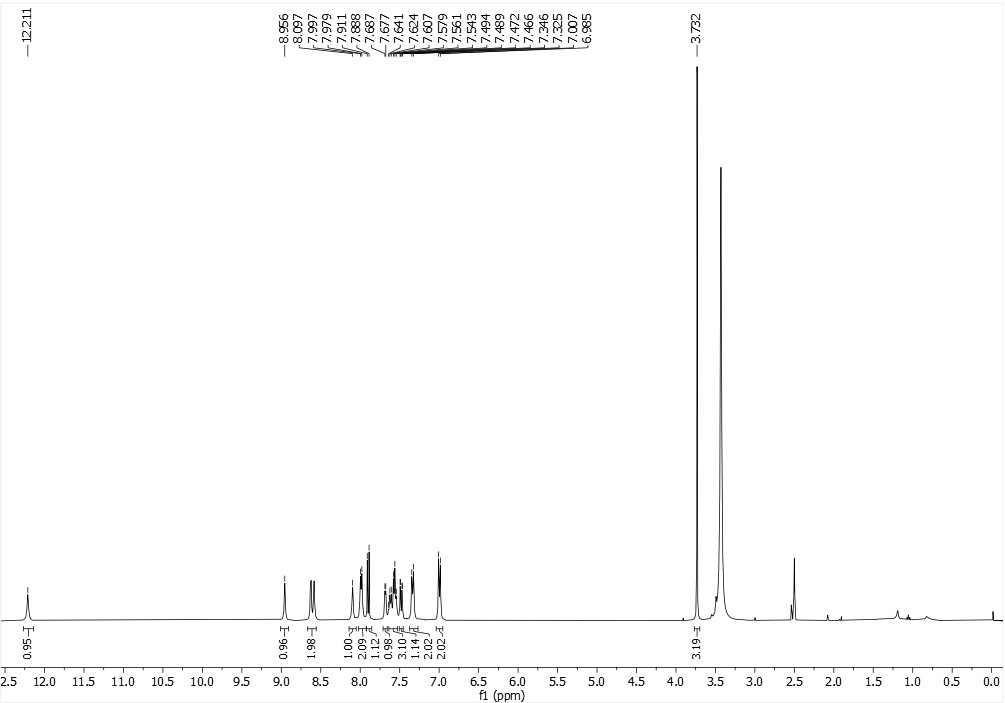


**Figure S23.** ^1^H NMR of *(E)*-*N*'-((6-(4-methoxyphenylamino)quinolin-4-yl)methylene)benzohydrazide (**11b**) (DMSO-*d*_6,_ 400 MHz)_._


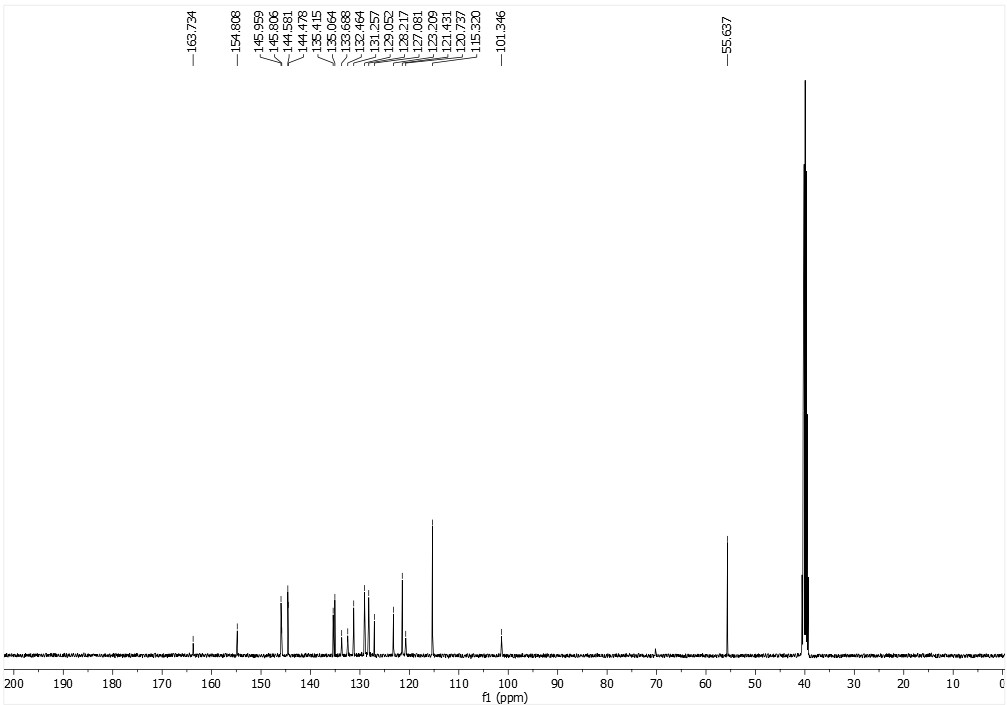


**Figure S24.** ^13^C NMR of *(E)*-*N*'-((6-(4-methoxyphenylamino)quinolin-4-yl)methylene)benzohydrazide (**11b**) (DMSO-*d*_6_, 400 MHz).

_
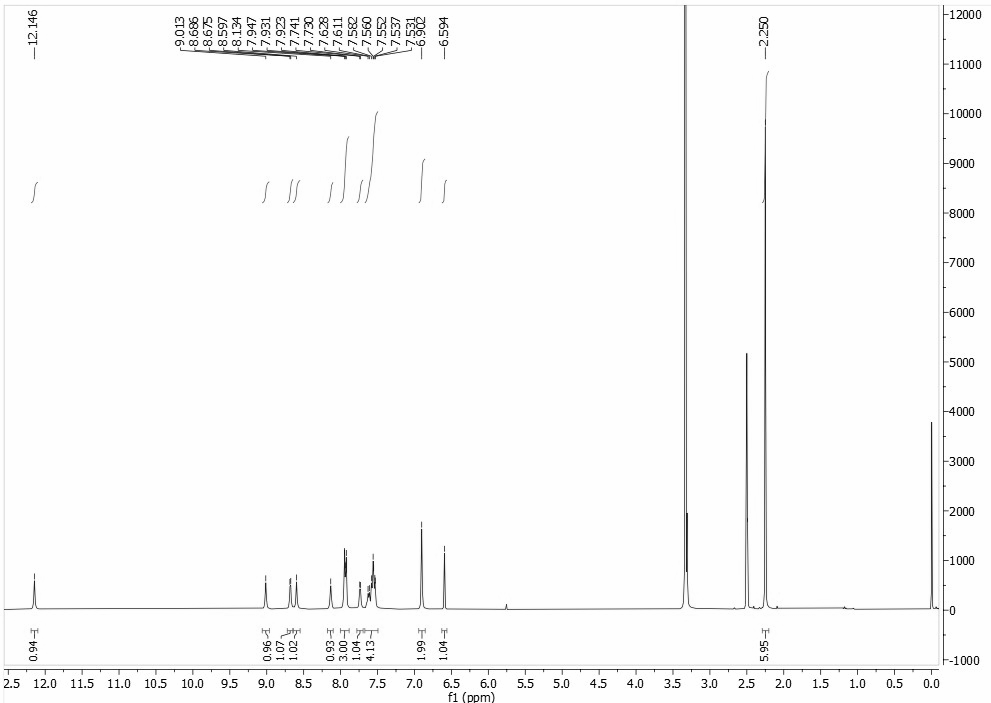
_

**Figure S25.** ^1^H NMR of *(E)*-*N*'-((6-((3,5-dimethylphenyl)amino)quinolin-4-yl)methylene)benzohydrazide (**11c**) (DMSO-*d*_6_, 400 MHz)_._


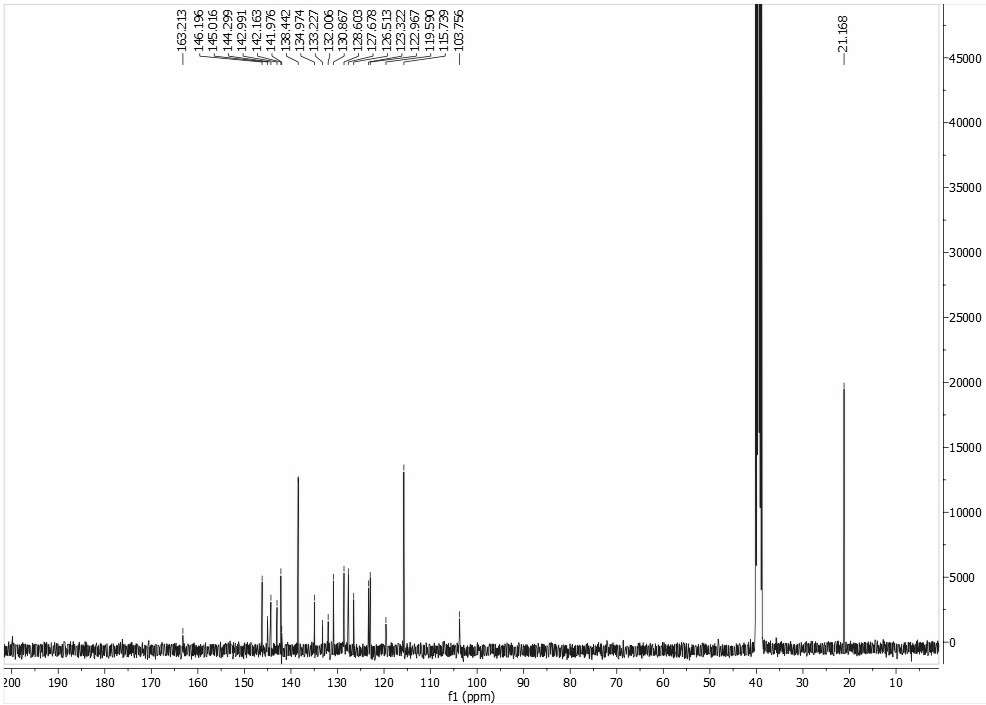


**Figure S26.** ^13^C NMR of *(E)*-*N*'-((6-((3,5-dimethylphenyl)amino)quinolin-4-yl)methylene)benzohydrazide (**11c**) (DMSO-*d*_6_, 100 MHz)_._

_
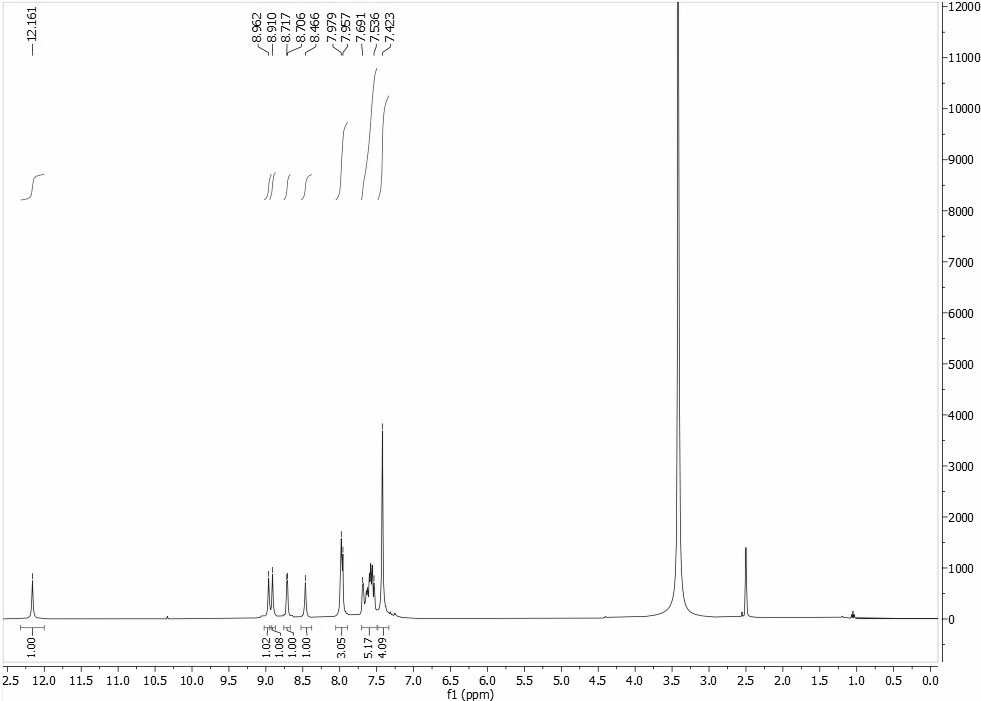
_

**Figure S27.** ^1^H NMR of *(E)*-*N*'-((6-((4-chlorophenyl)amino)quinolin-4 yl)methylene)benzohydrazide (**11d**) (DMSO-*d*_6_, 400 MHz)_._

_
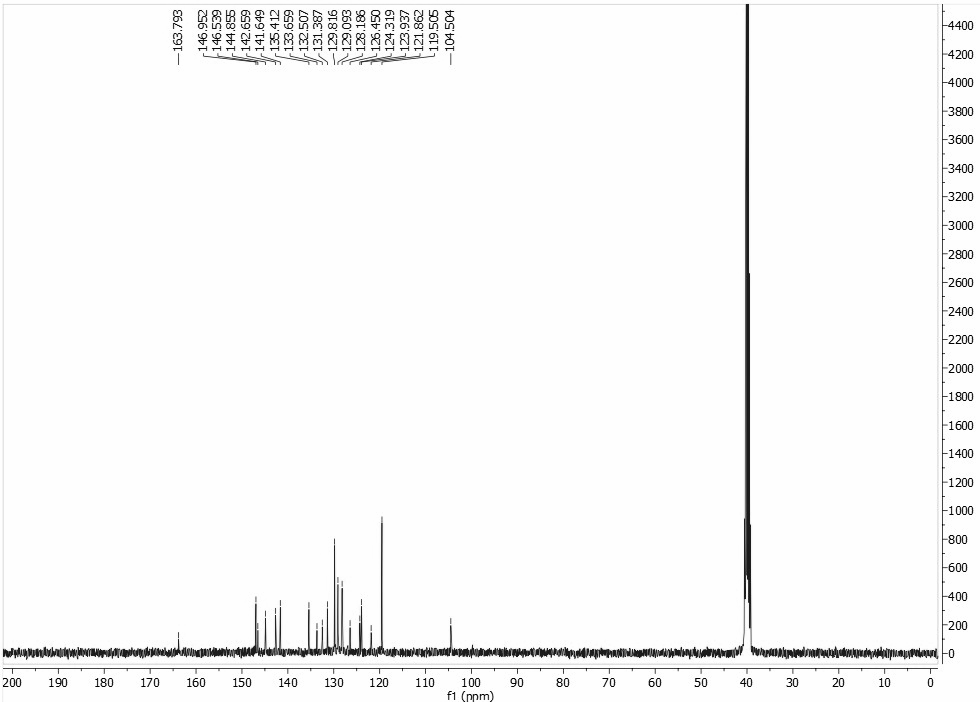
_

**Figure S28.** ^13^C NMR of *(E)*-*N*'-((6-((4-chlorophenyl)amino)quinolin-4 yl)methylene)benzohydrazide (**11d**) (DMSO-*d*_6_, 100 MHz)_._


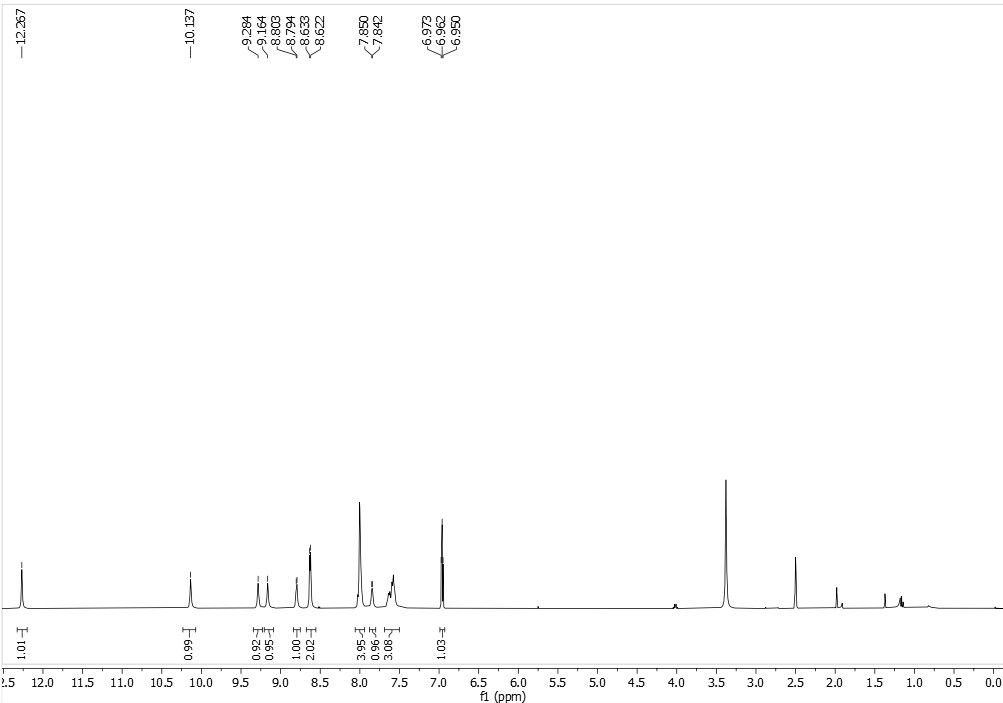


**Figure S29.** ^1^H NMR of *(E)*-*N*'-((6-(pyrimidin-2-ylamino)quinolin-4-yl)methylene)benzohydrazide (**11e**) (DMSO-*d*_6_, 400 MHz).


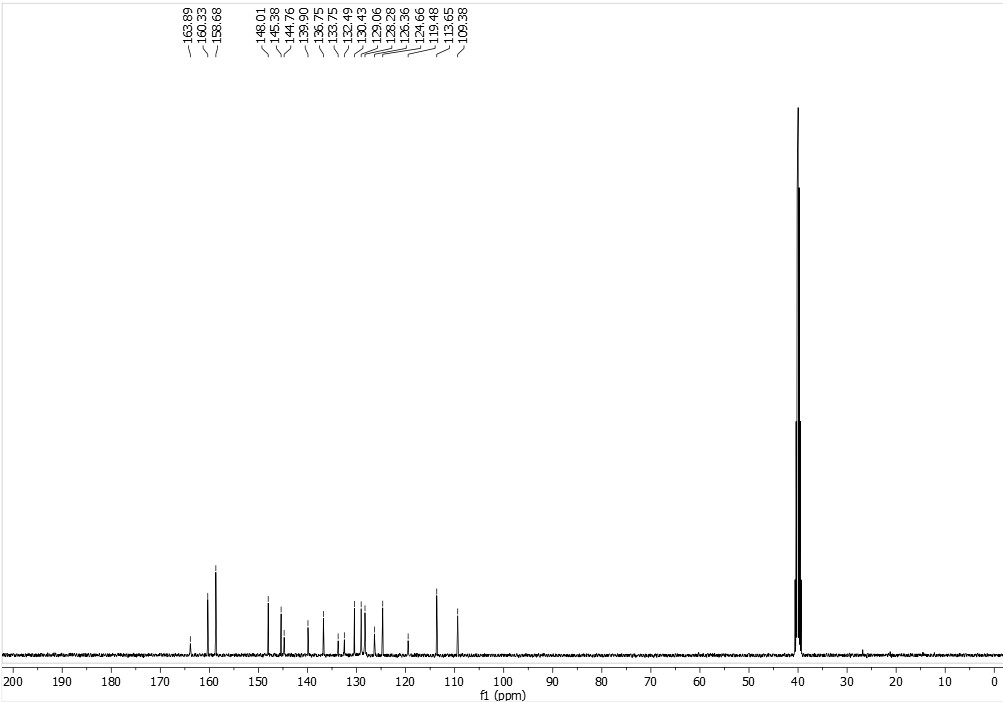


**Figure S30.** ^13^C NMR of *(E)*-*N*'-((6-(pyrimidin-2-ylamino)quinolin-4-yl)methylene)benzohydrazide (**11e**) (DMSO-*d*_6_, 100 MHz)_._

**
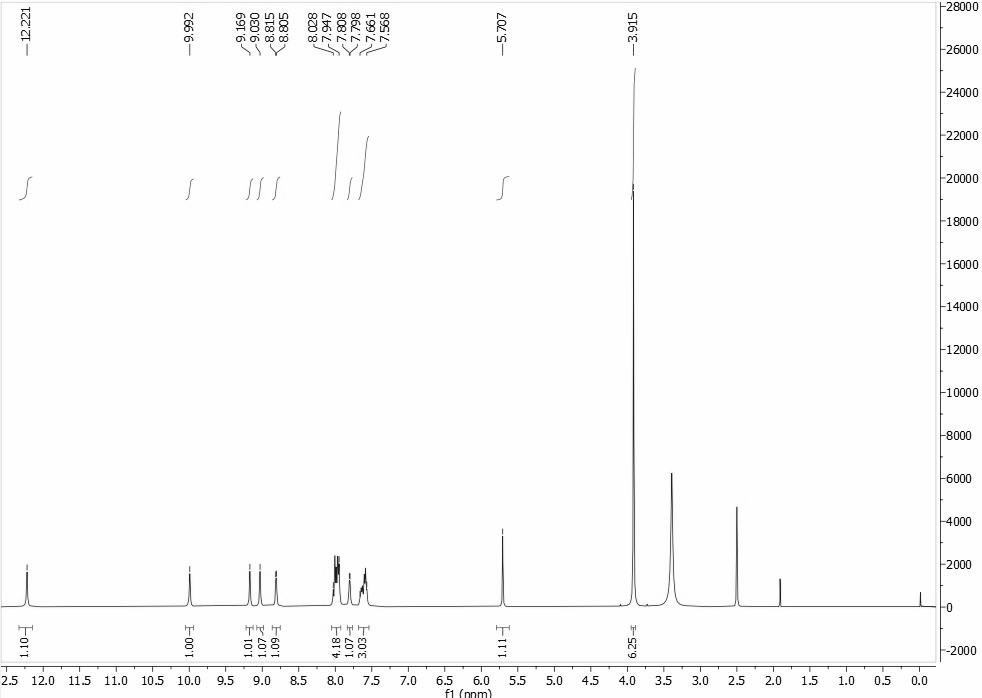
**

**Figure S31.** ^1^H NMR of *(E)*-*N*'-((6-((4,6-dimethoxypyrimidin-2-yl)amino)quinolin-4-yl)methylene)benzohydrazide (**11f**) (DMSO-*d*_6_, 400 MHz)_._

_
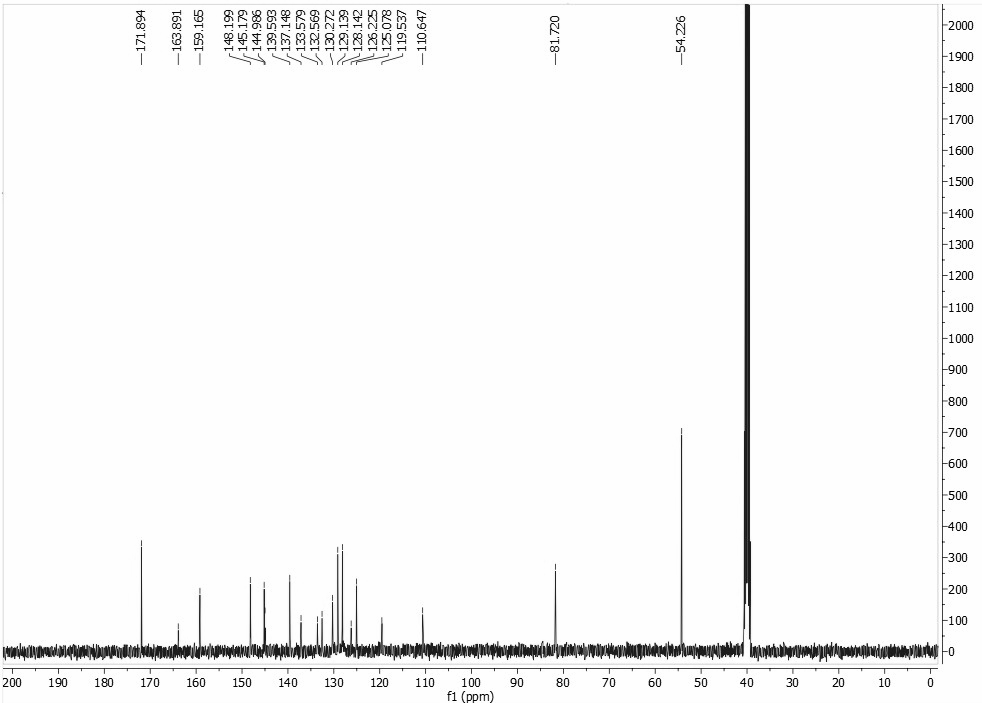
_

**Figure S32.** ^13^C NMR of *(E)*-*N*'-((6-((4,6-dimethoxypyrimidin-2-yl)amino)quinolin-4-yl)methylene)benzohydrazide (**11f**) (DMSO-*d*_6_, 100 MHz)_._

_
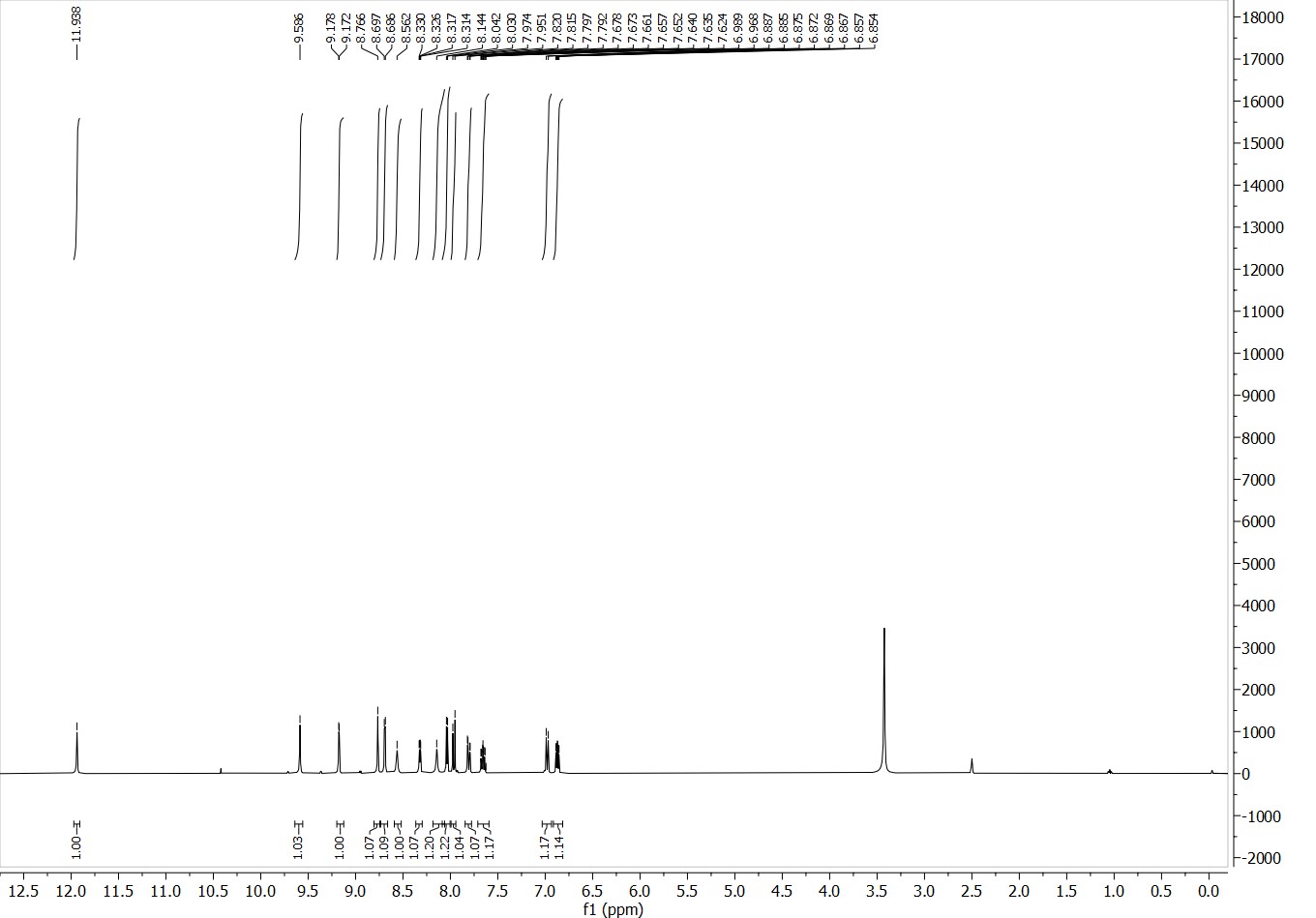
_

**Figure S33.** ^1^H NMR of (*E*)-2-((6-(pyridin-2-ylamino)quinolin-4-yl)methylene)hydrazine-1-carbothioamide (**3a**) (DMSO-*d*_6_, 400 MHz)_._


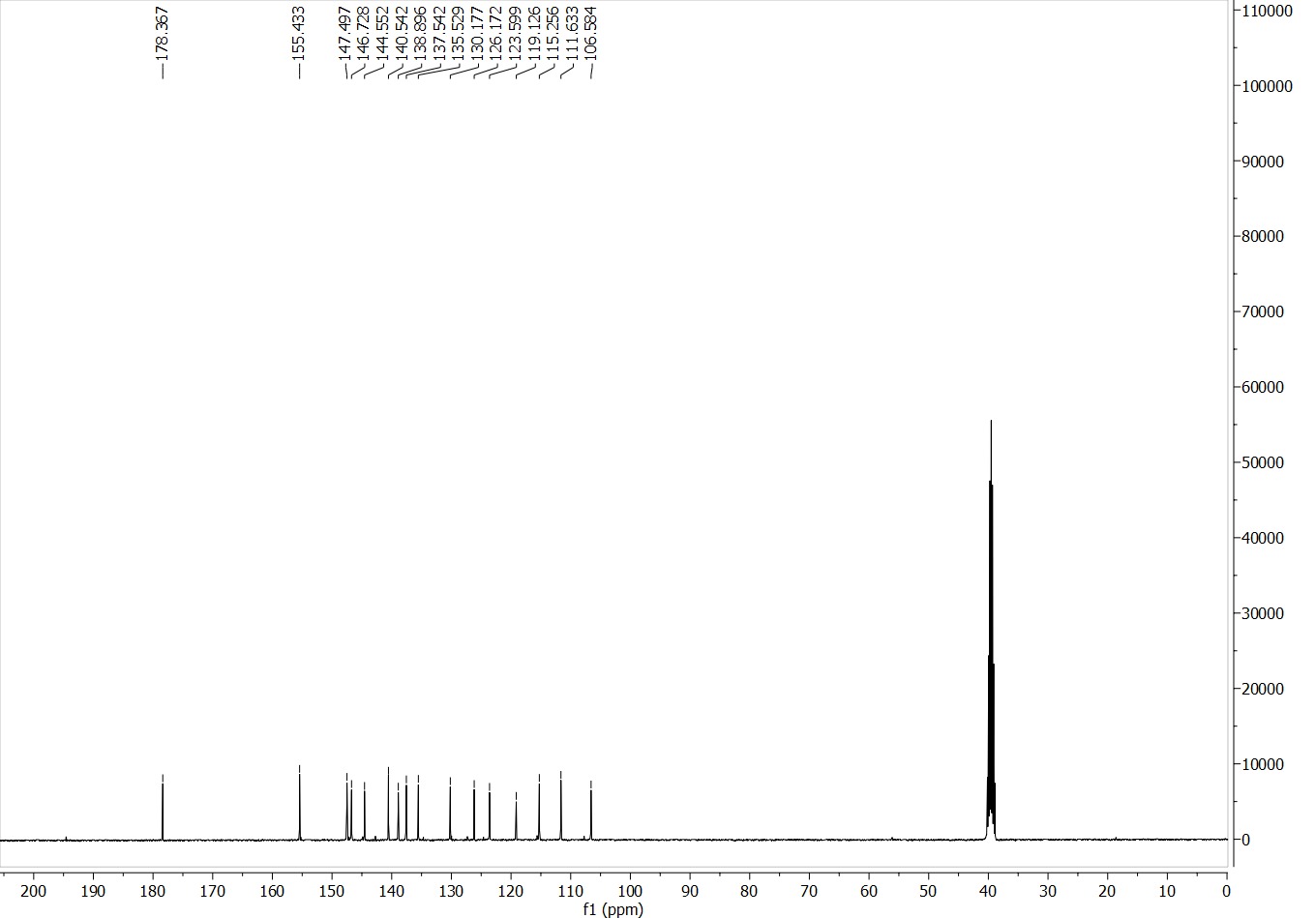


**Figure S34.** ^13^C NMR of (*E*)-2-((6-(pyridin-2-ylamino)quinolin-4-yl)methylene)hydrazine-1-carbothioamide (**3a**) (DMSO-*d*_6_, 400 MHz)_._


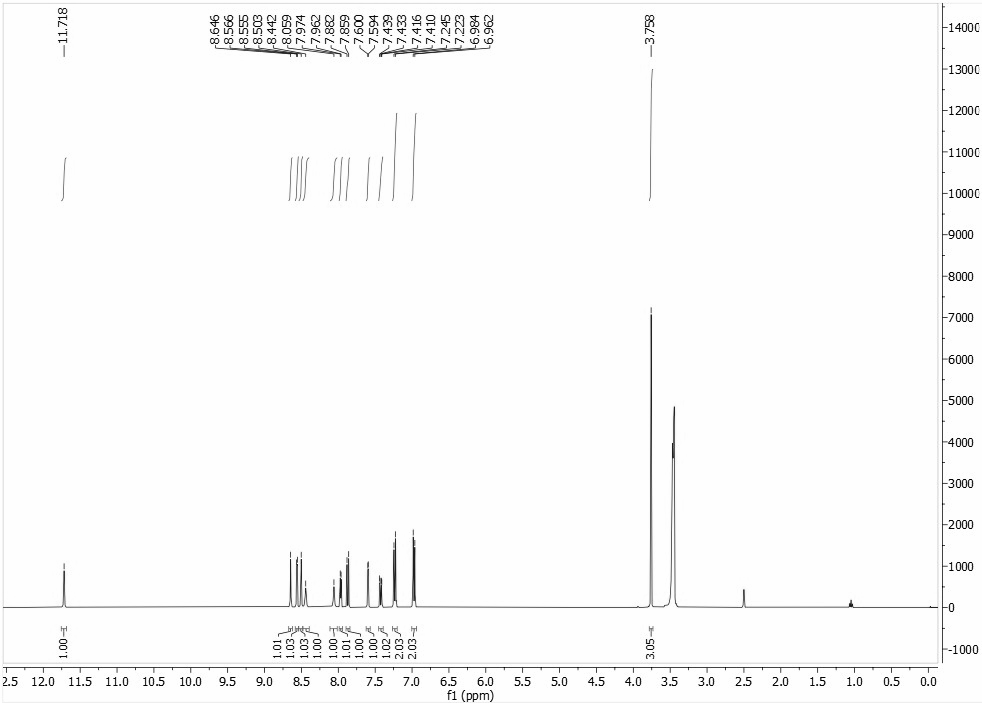


**Figure S35.** ^1^H NMR of *(E)*-2-((6-((4-methoxyphenyl)amino)quinolin-4-yl)methylene)hydrazinecarbothioamide (**3b**) in (DMSO-*d*_6_, 400 MHz)_._

_
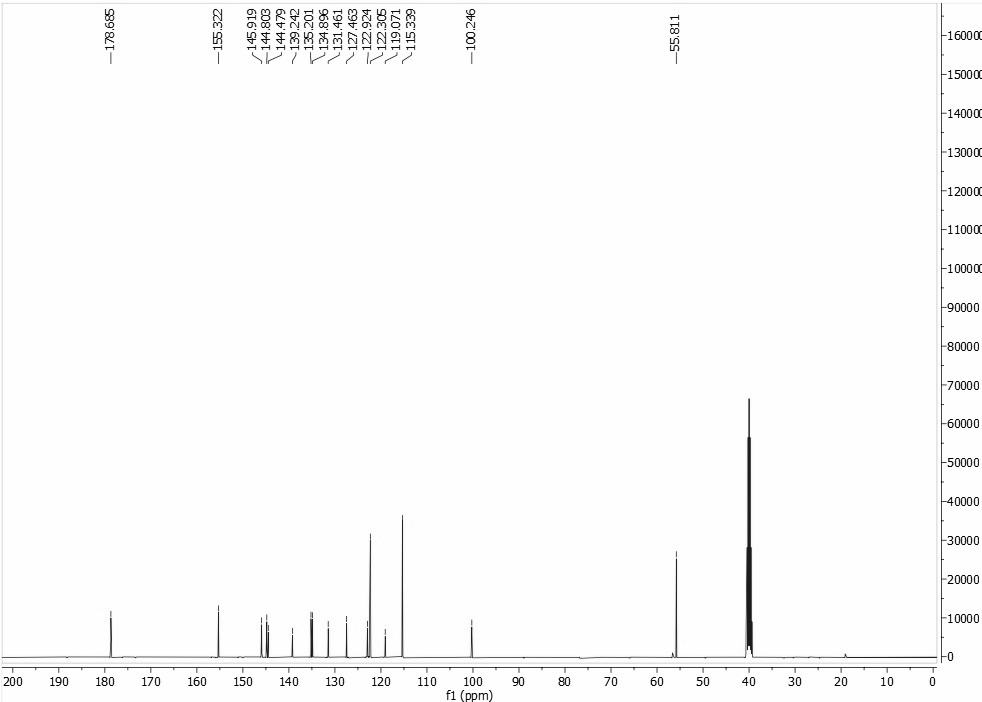
_

**Figure S36.** ^13^C NMR of (*E*)-2-((6-((4-methoxyphenyl)amino)quinolin-4-yl)methylene)hydrazinecarbothioamide (**3b**) (DMSO-*d*_6_, 100 MHz)_._

_
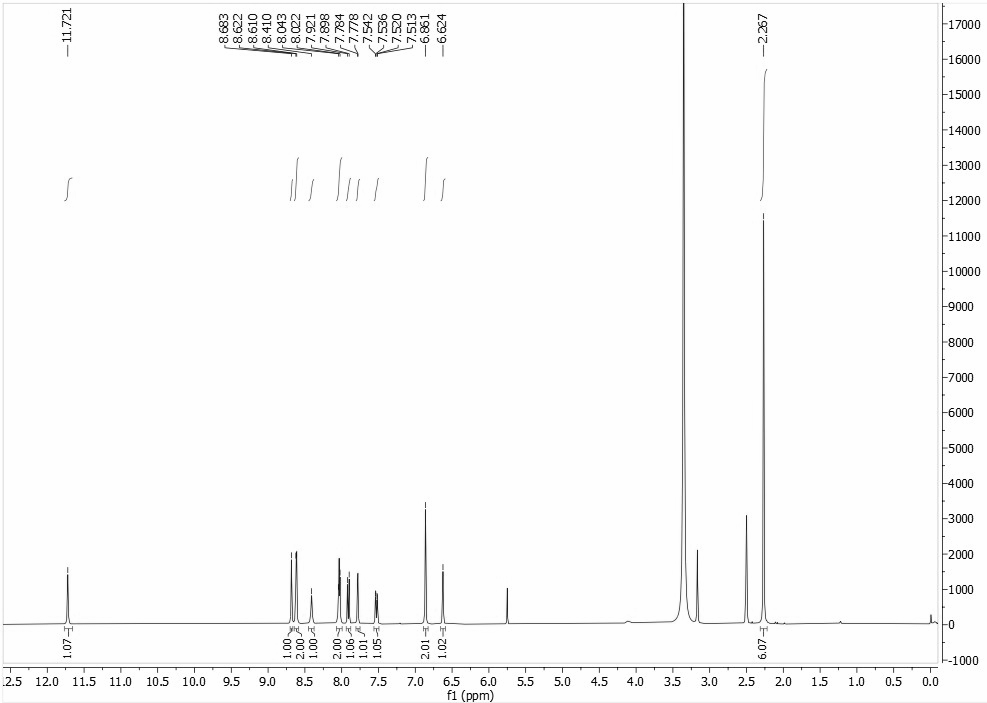
_

**Figure S37.** ^1^H NMR of *(E)*-2-((6-((3,5-dimethylphenyl)amino)quinolin-4-yl)methylene)hydrazine-1-carbothioamide (**3c**) (DMSO-*d*_6_, 400 MHz)_._

_
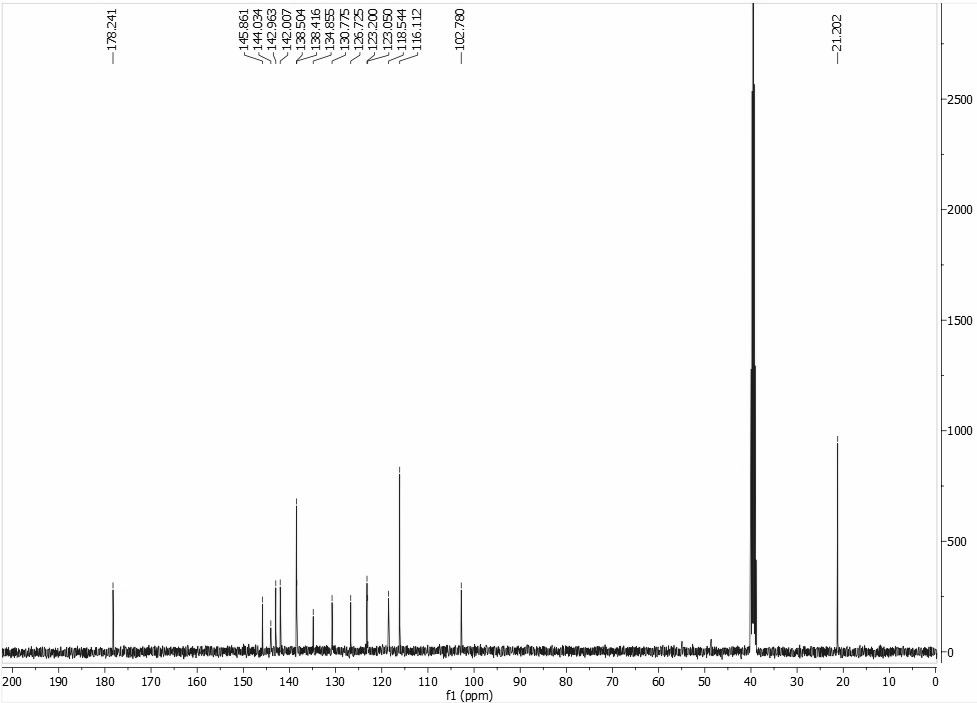
_

**Figure S38.** ^13^C NMR of *(E)*-2-((6-((3,5-dimethylphenyl)amino)quinolin-4-yl)methylene)hydrazine-1-carbothioamide (**3c**) (DMSO-*d*_6_, 400 MHz)_._

_
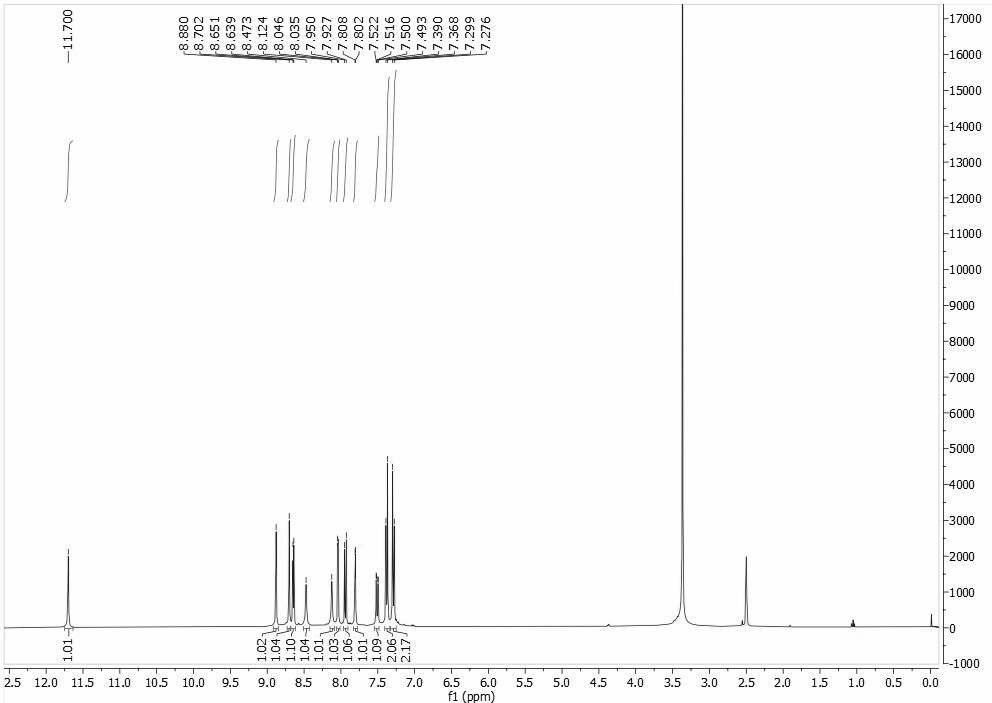
_

**Figure S39.** ^1^H NMR of *(E)*-2-((6-((4-chlorophenyl)amino)quinolin-4-yl)methylene)hydrazinecarbothioamide (**3d**) (DMSO-*d*_6_, 400 MHz)_._

_
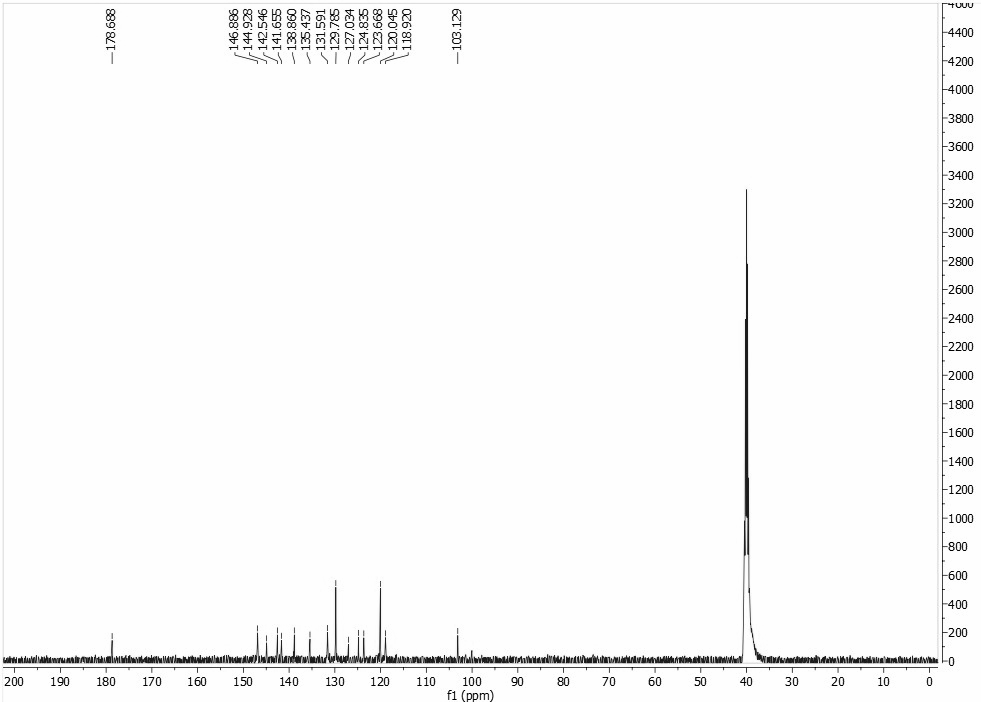
_

**Figure S40.** ^13^C NMR of *(E)*-2-((6-((4-chlorophenyl)amino)quinolin-4-yl)methylene)hydrazinecarbothioamide (**3d**) (DMSO-*d*_6_, 100 MHz)_._


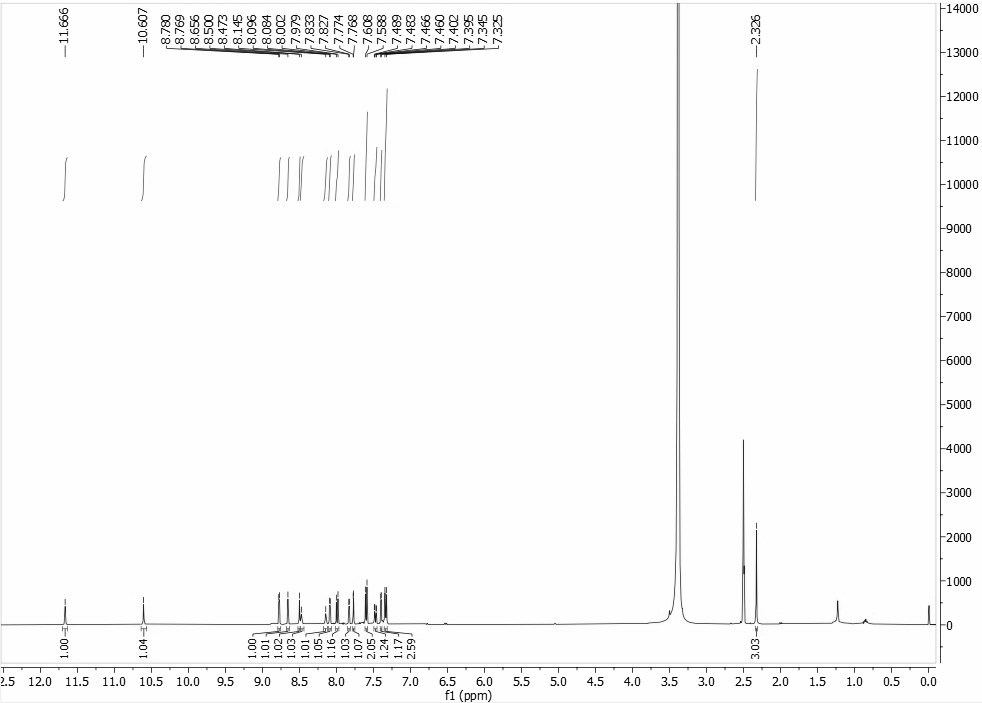


**Figure S41**. ^1^H NMR of *(E)*-2-((6-((2-chloro-5-((4-methylphenyl)sulfonamido)pyridin-3-yl)amino)quinolin-4-yl)methylene)hydrazine-1-carbothioamide (**3e**) (DMSO-*d*_6_, 400 MHz)_._


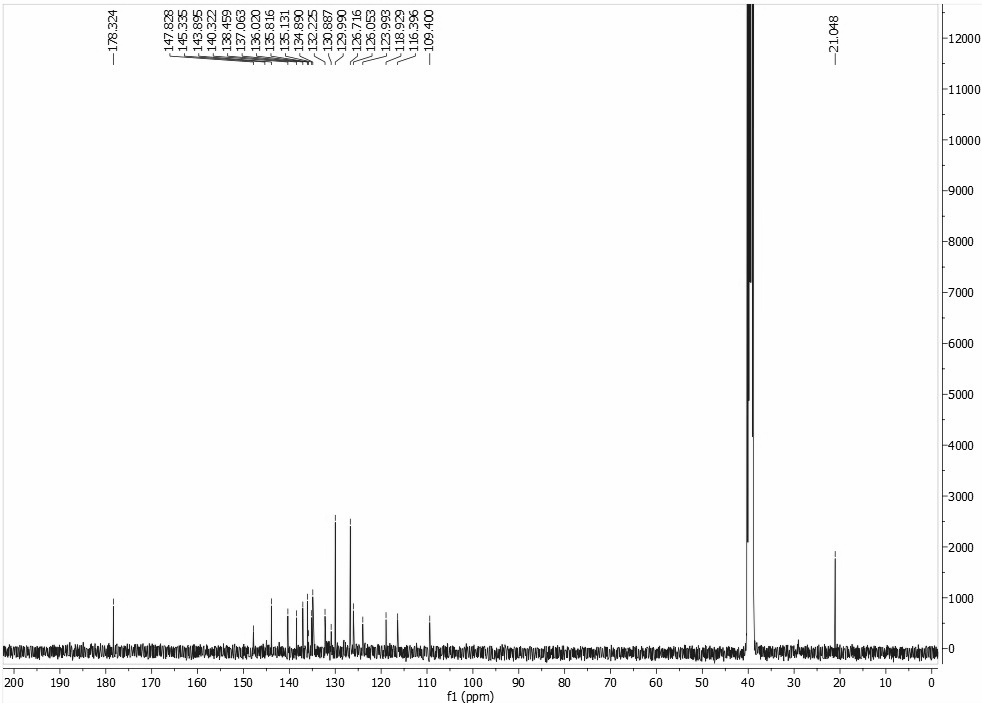


**Figure S42.** ^13^C NMR of *(E)*-2-((6-((2-chloro-5-((4-methylphenyl)sulfonamido)pyridin-3-yl)amino)quinolin-4-yl)methylene)hydrazine-1-carbothioamide (**3e**) (DMSO-*d*_6_, 100 MHz)_._

_
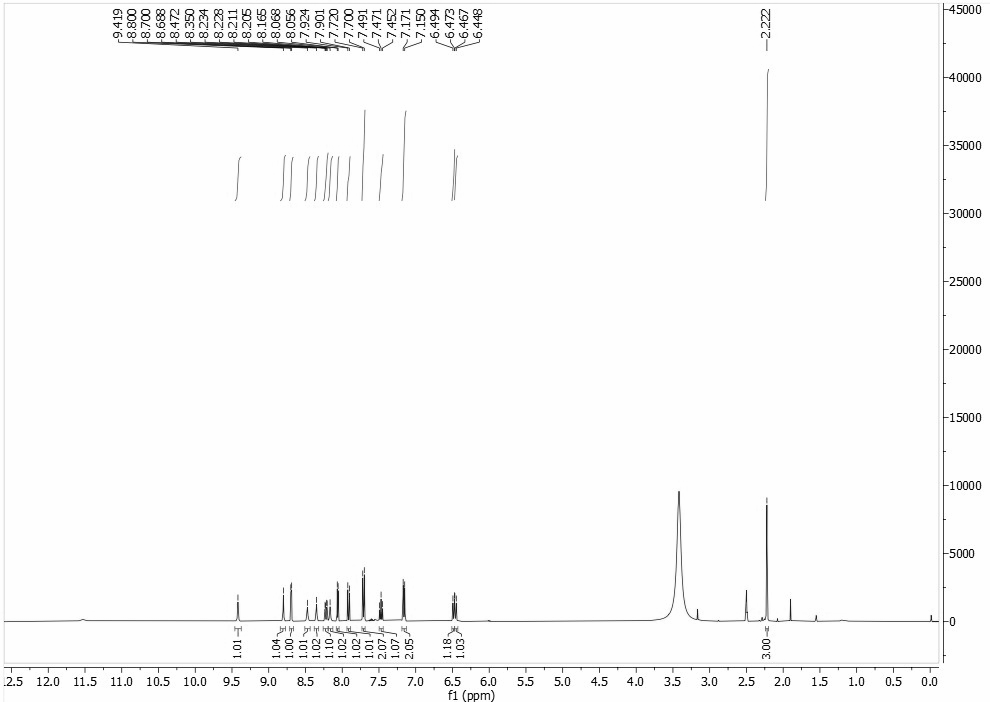
_

**Figure S43.** ^1^H NMR of *(E)*-2-((6-((6-((4-methylphenyl)sulfonamido)pyridin-2-yl)amino)quinolin-4-yl)methylene)hydrazine-1-carbothioamide (**3f**) (DMSO-*d*_6_, 400 MHz).


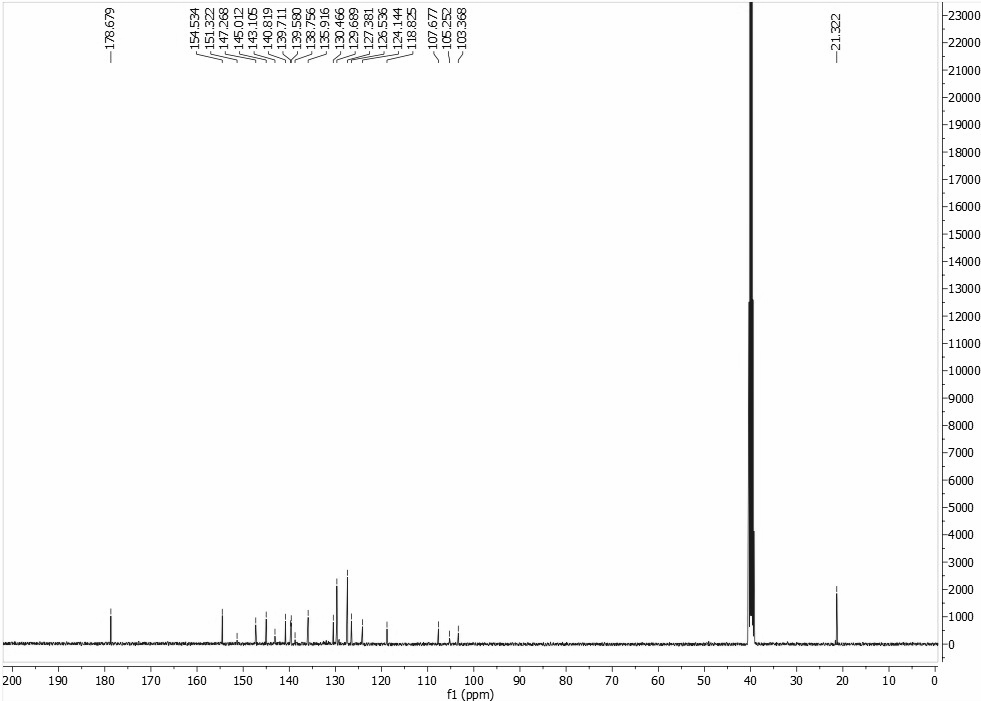


**Figure S44**. ^13^C NMR of *(E)*-2-((6-((6-((4-methylphenyl)sulfonamido)pyridin-2-yl)amino)quinolin-4-yl)methylene)hydrazine-1-carbothioamide (**3f**) (DMSO-*d*_6_, 100 MHz)_._

**
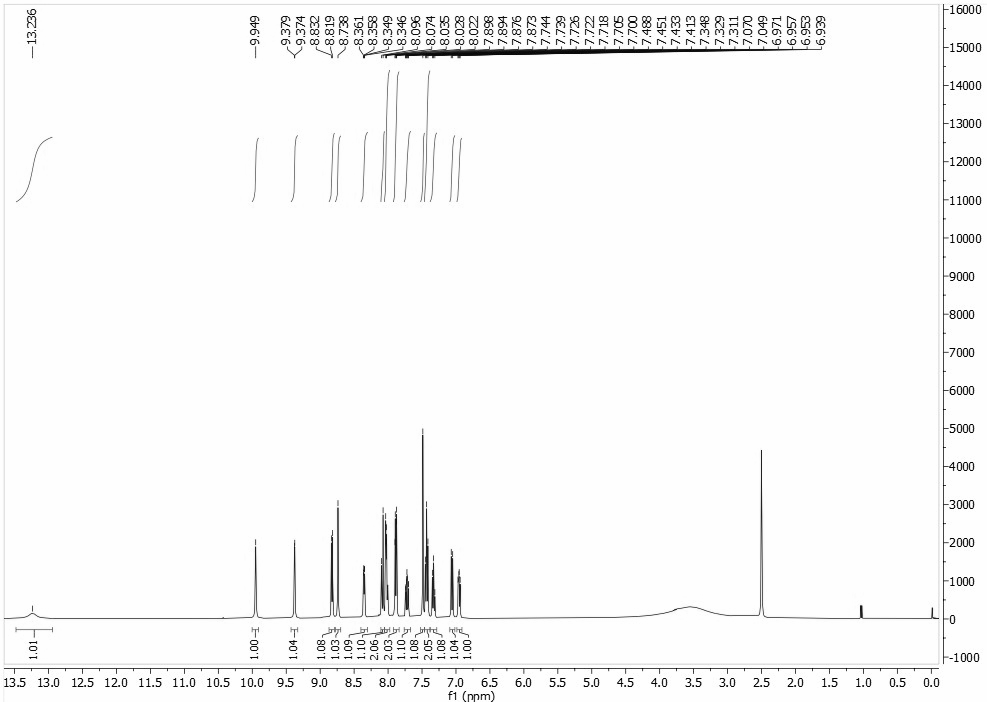
**

**Figure S45.** ^1^H NMR of (*E*)-4-((2-(4-phenylthiazol-2-yl)hydrazineylidene)methyl)-*N*-(pyridin-2-yl)quinolin-6-amine (**12a**) (DMSO-*d*_6_, 400 MHz)_._

**
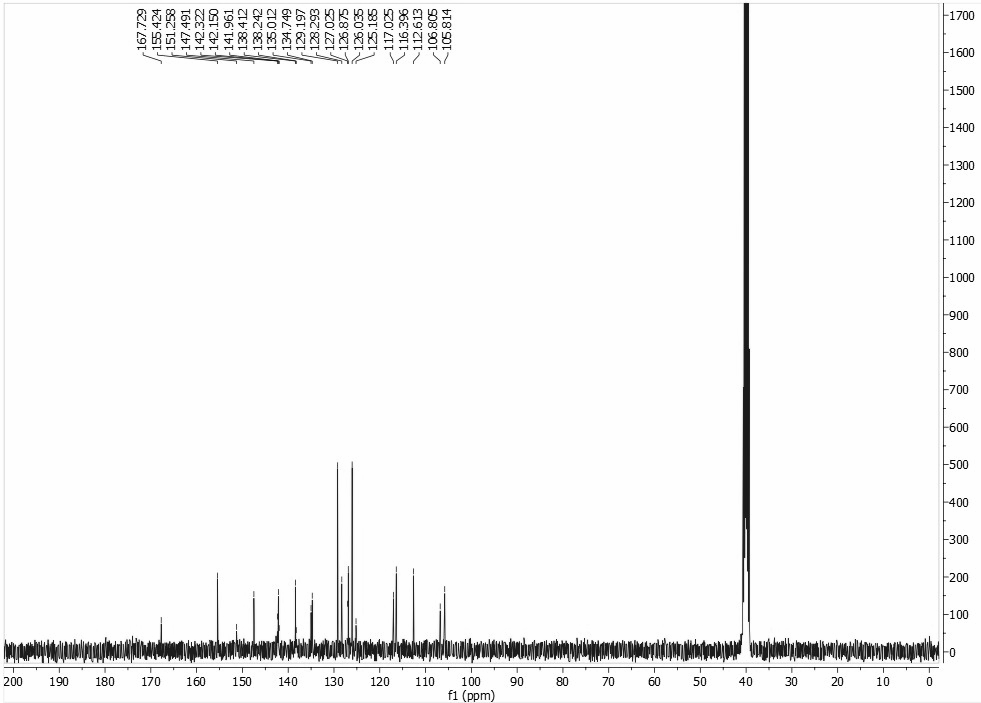
**

**Figure S46.** ^13^C NMR of (*E*)-4-((2-(4-phenylthiazol-2-yl)hydrazineylidene)methyl)-*N*-(pyridin-2-yl)quinolin-6-amine (**12a**) (DMSO-*d*_6_, 100 MHz)_._

**
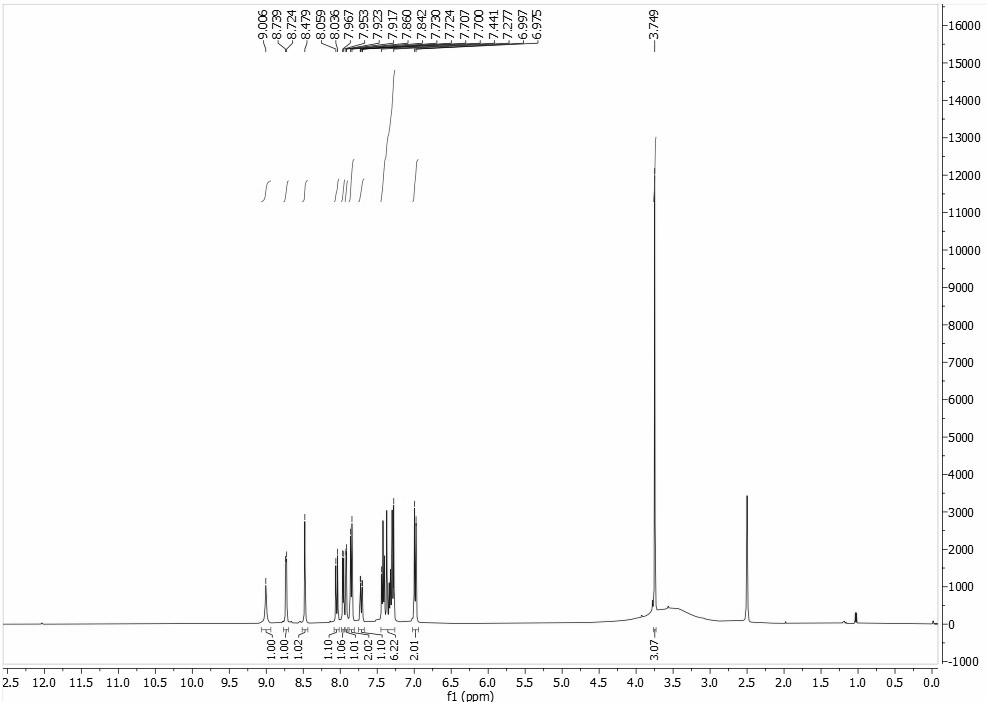
**

**Figure S47.** ^1^H NMR of *(E)*-*N*-(4-methoxyphenyl)-4-((2-(4-phenylthiazol-2-yl)hydrazineylidene)methyl)quinolin-6-amine (**12b**) (DMSO-*d*_6_, 400 MHz)_._

**
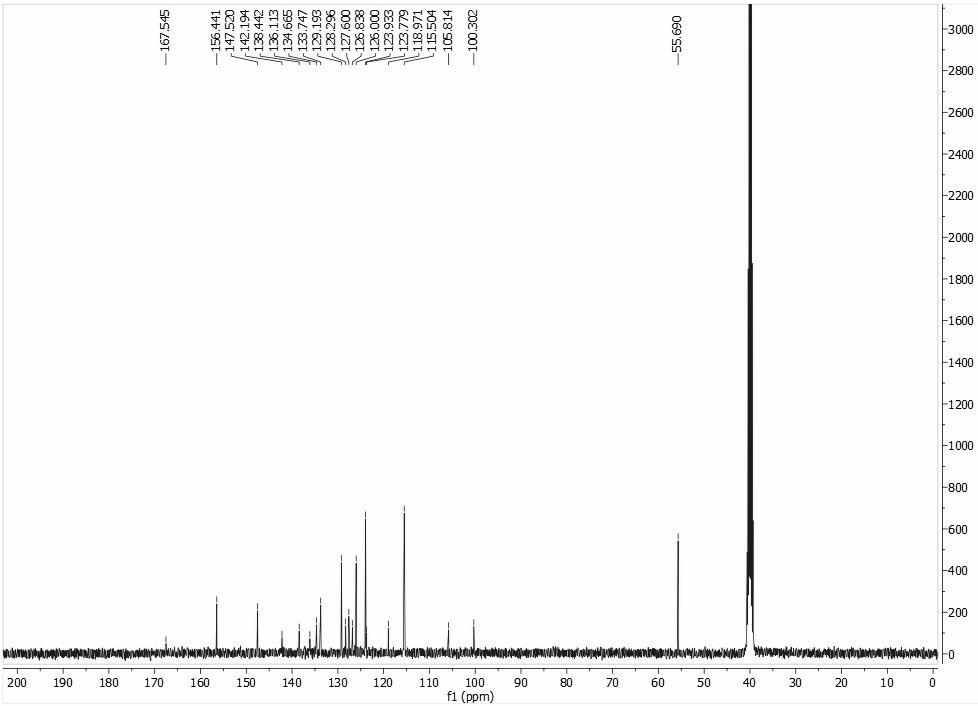
**

**Figure S48.** ^13^C NMR of *(E)*-*N*-(4-methoxyphenyl)-4-((2-(4-phenylthiazol-2-yl)hydrazineylidene)methyl)quinolin-6-amine (**12b**) (DMSO-*d*_6_, 100 MHz)_._

**
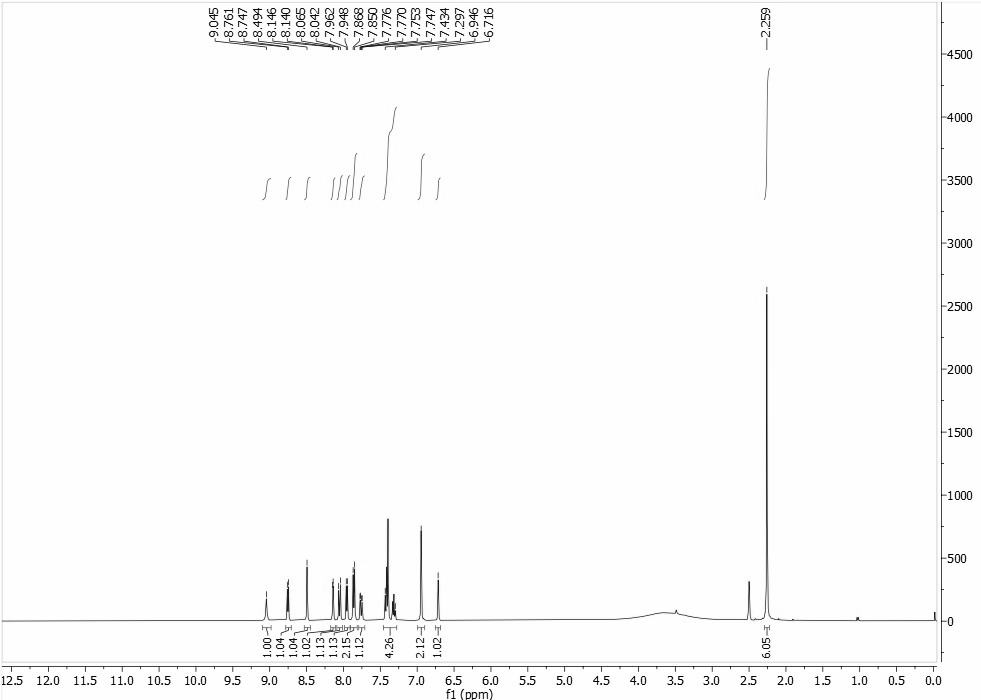
**

**Figure S49.** ^1^H NMR of *(E)*-*N*-(3,5-dimethylphenyl)-4-((2-(4-phenylthiazol-2-yl)hydrazineylidene)methyl)quinolin-6-amine (**12c**) (DMSO-*d*_6_, 400 MHz)_._

**
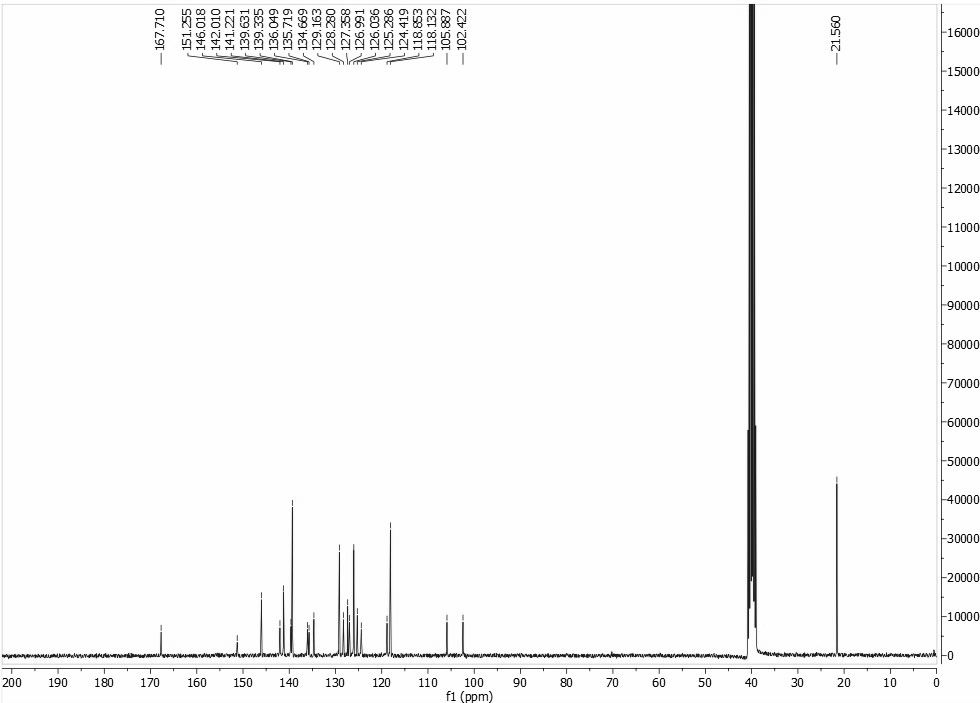
**

**Figure S50.** ^13^C NMR of *(E)*-*N*-(3,5-dimethylphenyl)-4-((2-(4-phenylthiazol-2-yl)hydrazineylidene)methyl)quinolin-6-amine (**12c**) (DMSO-*d*_6_, 100 MHz).


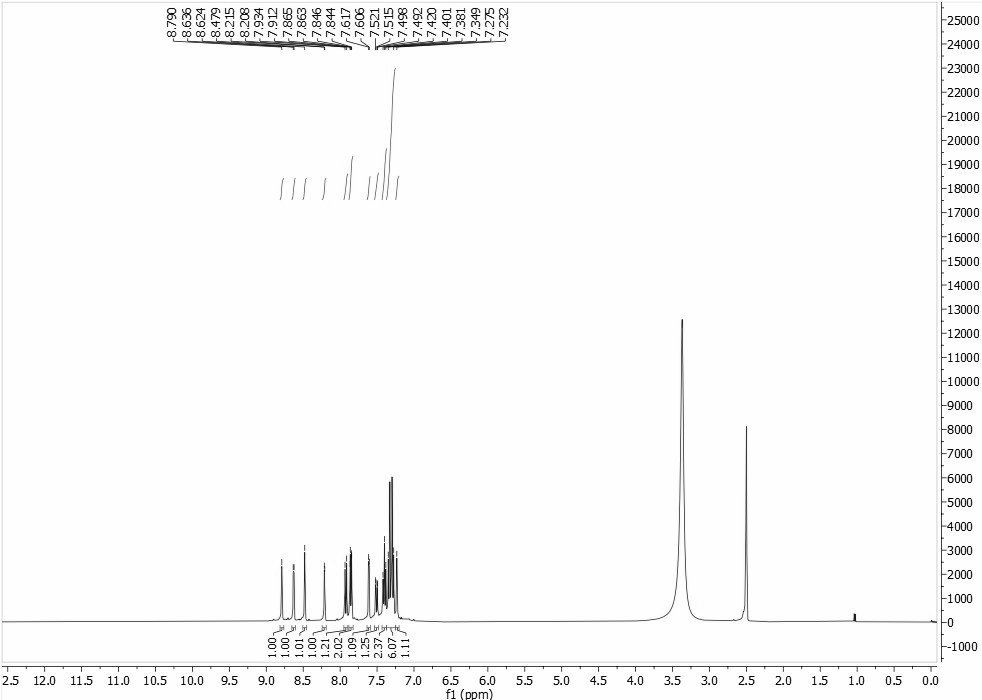


**Figure S51.** ^1^H NMR of *(E)*-*N*-(4-chlorophenyl)-4-((2-(4-phenylthiazol-2-yl)hydrazineylidene)methyl)quinolin-6-amine (**12d**) (DMSO-*d*_6_, 400 MHz)_._

**
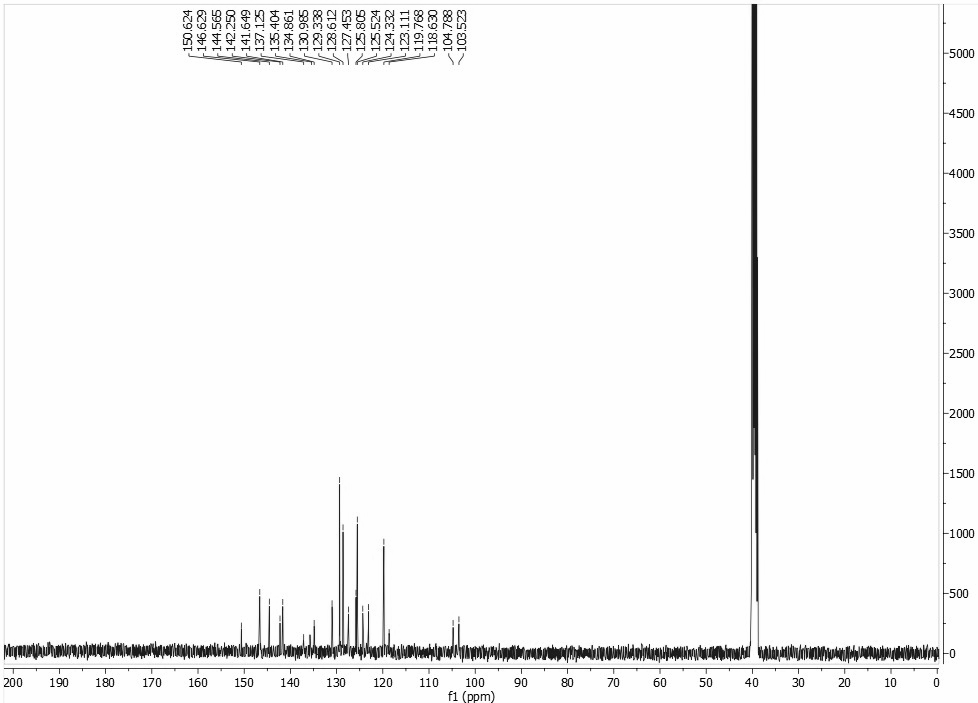
**

**Figure S52.** ^13^C NMR of *(E)*-*N*-(4-chlorophenyl)-4-((2-(4-phenylthiazol-2-yl)hydrazineylidene)methyl)quinolin-6-amine (**12d**) (DMSO-*d*_6_, 100 MHz)_._

**
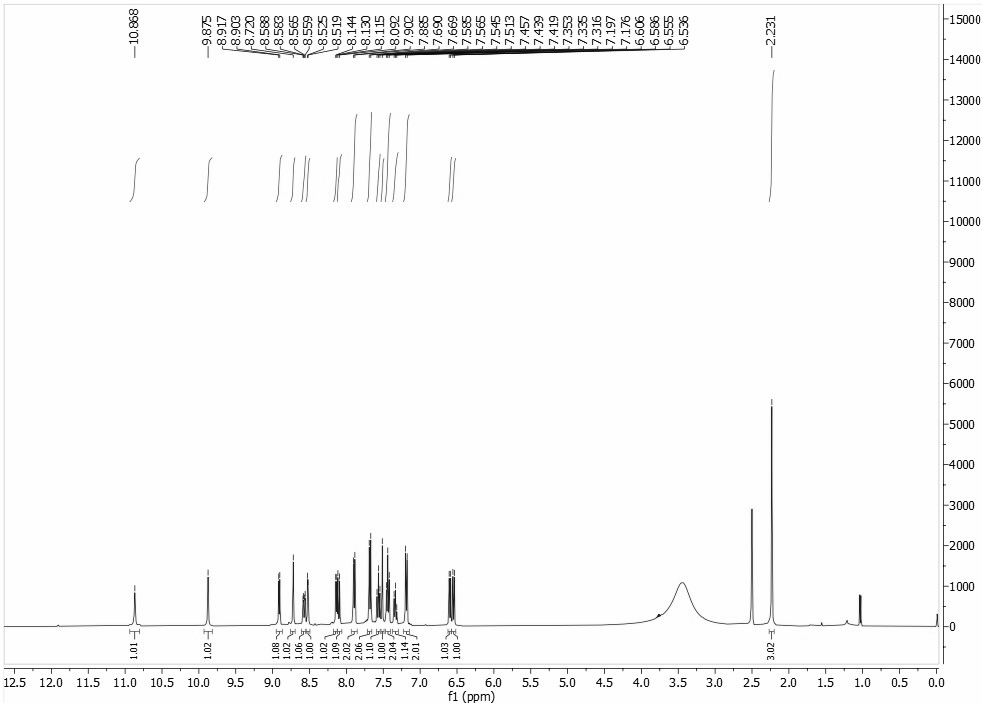
**

**Figure S53.** ^1^H NMR of *(E)*-4-methyl-*N*-(6-((4-((2-(4-phenylthiazol-2-yl)hydrazineylidene)methyl)quinolin-6-yl)amino)pyridin-2-yl)benzenesulfonamide (**12e**) (DMSO-*d*_6_, 400 MHz)_._

**
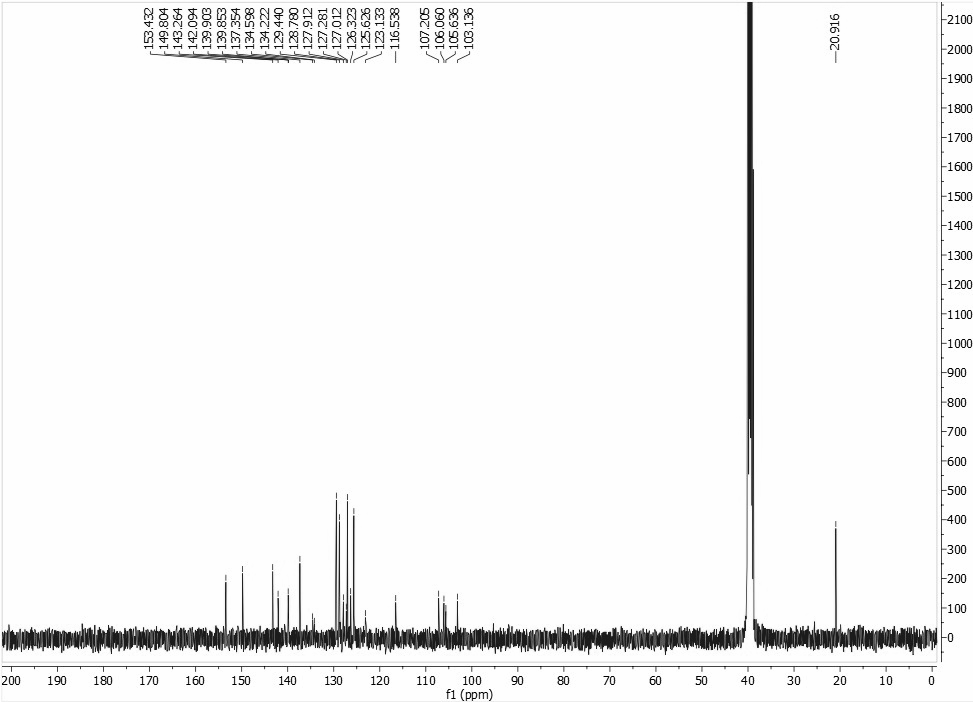
**

**Figure S54.** ^13^C NMR of *(E)*-4-methyl-*N*-(6-((4-((2-(4-phenylthiazol-2-yl)hydrazineylidene)methyl)quinolin-6-yl)amino)pyridin-2-yl)benzenesulfonamide (**12e**) (DMSO-*d*_6_, 100 MHz)_._

**HRMS data**

**Figure S55.** HRMS spectrum of *(E)*-*N*'-((6-(pyridin-2-ylamino)quinolin-4-yl)methylene)benzohydrazide (**11a**).

**Figure S56.** HRMS spectrum of *(E)*-*N*'-((6-(4-methoxyphenylamino)quinolin-4yl)methylene)benzohydrazide (**11b**).

**Figure S57.** HRMS spectrum of *(E)*-*N*'-((6-((3,5-dimethylphenyl)amino)quinolin-4-yl)methylene)benzohydrazide (**11c**).

**Figure S58.** HRMS spectrum of *(E)*-*N*'-((6-((4-chlorophenyl)amino)quinolin-4 yl)methylene)benzohydrazide (**11d**).

**Figure S59.** HRMS spectrum of *(E)*-*N*'-((6-(pyrimidin-2-ylamino)quinolin-4-yl)methylene)benzohydrazide (**11e**).

**Figure S60.** HRMS spectrum of *(E)*-*N*'-((6-((4,6-dimethoxypyrimidin-2-yl)amino)quinolin-4-yl)methylene)benzohydrazide (**11f**).

**Figure S61.** HRMS spectrum of (*E*)-2-((6-(pyridin-2-ylamino)quinolin-4-yl)methylene)hydrazine-1-carbothioamide (**3a**).

**Figure S62.** HRMS spectrum of *(E)*-2-((6-((4-methoxyphenyl)amino)quinolin-4-yl)methylene)hydrazinecarbothioamide (**3b**).

**Figure S63.** HRMS spectrum of *(E)*-2-((6-((3,5-dimethylphenyl)amino)quinolin-4-yl)methylene)hydrazine-1-carbothioamide (**3c**).

**Figure S64.** HRMS spectrum of *(E)*-2-((6-((4-chlorophenyl)amino)quinolin-4-yl)methylene)hydrazinecarbothioamide (**3d**).

**Figure S65.** HRMS spectrum of (*E*)-2-((6-((2-chloro-5-((4-methylphenyl)sulfonamido)pyridin-3-yl)amino)quinolin-4-yl)methylene)hydrazine-1-carbothioamide (**3e**).

**Figure S66.** HRMS spectrum of *(E)*-2-((6-((6-((4-methylphenyl)sulfonamido)pyridin-2-yl)amino)quinolin-4-yl)methylene)hydrazine-1-carbothioamide (**3f**).

**Figure S67.** HRMS spectrum of *(E)*-4-((2-(4-phenylthiazol-2-yl)hydrazineylidene)methyl)-*N*-(pyridin-2-yl)quinolin-6-amine (**12a**).

**Figure S68.** HRMS spectrum of *(E)*-*N*-(4-methoxyphenyl)-4-((2-(4-phenylthiazol-2-yl)hydrazineylidene)methyl)quinolin-6-amine (**12b**).

**Figure S69.** HRMS spectrum of *(E)*-*N*-(3,5-dimethylphenyl)-4-((2-(4-phenylthiazol-2-yl)hydrazineylidene)methyl)quinolin-6-amine (**12c**).

**Figure S70.** HRMS spectrum of *(E)*-*N*-(4-chlorophenyl)-4-((2-(4-phenylthiazol-2-yl)hydrazineylidene)methyl)quinolin-6-amine (**12d**).

**Figure S71.** HRMS spectrum of *(E)*-4-methyl-*N*-(6-((4-((2-(4-phenylthiazol-2-yl)hydrazineylidene)methyl)quinolin-6-yl)amino)pyridin-2-yl)benzenesulfonamide (**12e**).

**Figure S72.** HRMS spectrum of *N*-(5-amino-6-chloropyridin-3-yl)-4-methylbenzenesulfonamide (**16**).

**Figure S73.** HRMS spectrum of *N*-(6-aminopyridin-2-yl)-4-methylbenzenesulfonamide (**18**).

**HPLC data**


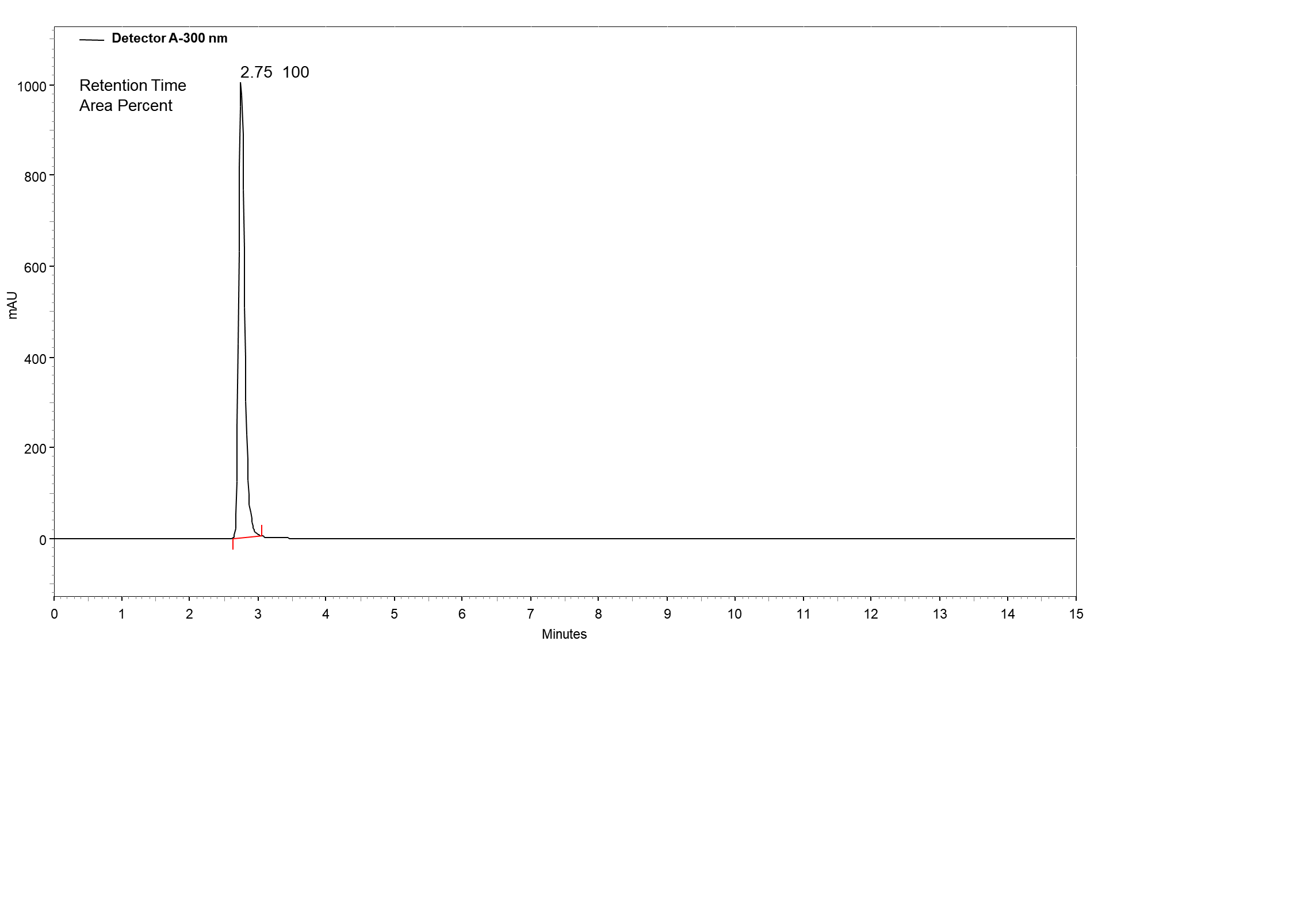


**Figure S74.** HPLC chromatogram of *(E)*-N'-((6-(pyridin-2-ylamino)quinolin-4-yl)methylene)benzohydrazide (**11a**). Mobile phase: ACN 50:50 TFA 0.1%.


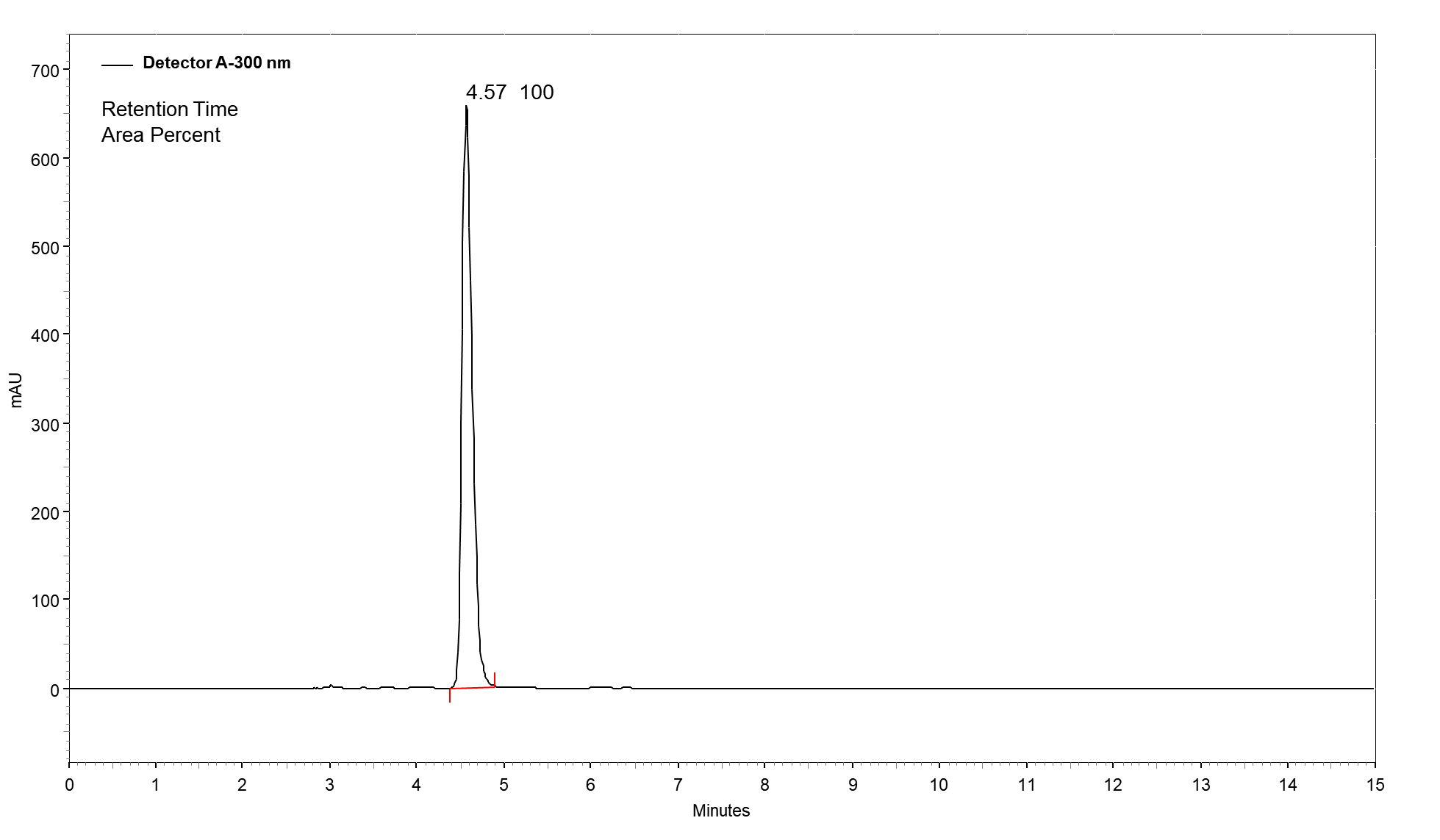


**Figure S75.** HPLC chromatogram of (*E*)-*N*'-((6-((4-methoxyphenyl)amino)quinolin-4-yl)methylene)benzohydrazide (**11b**). Mobile phase: ACN 50:50 TFA 0.1%.


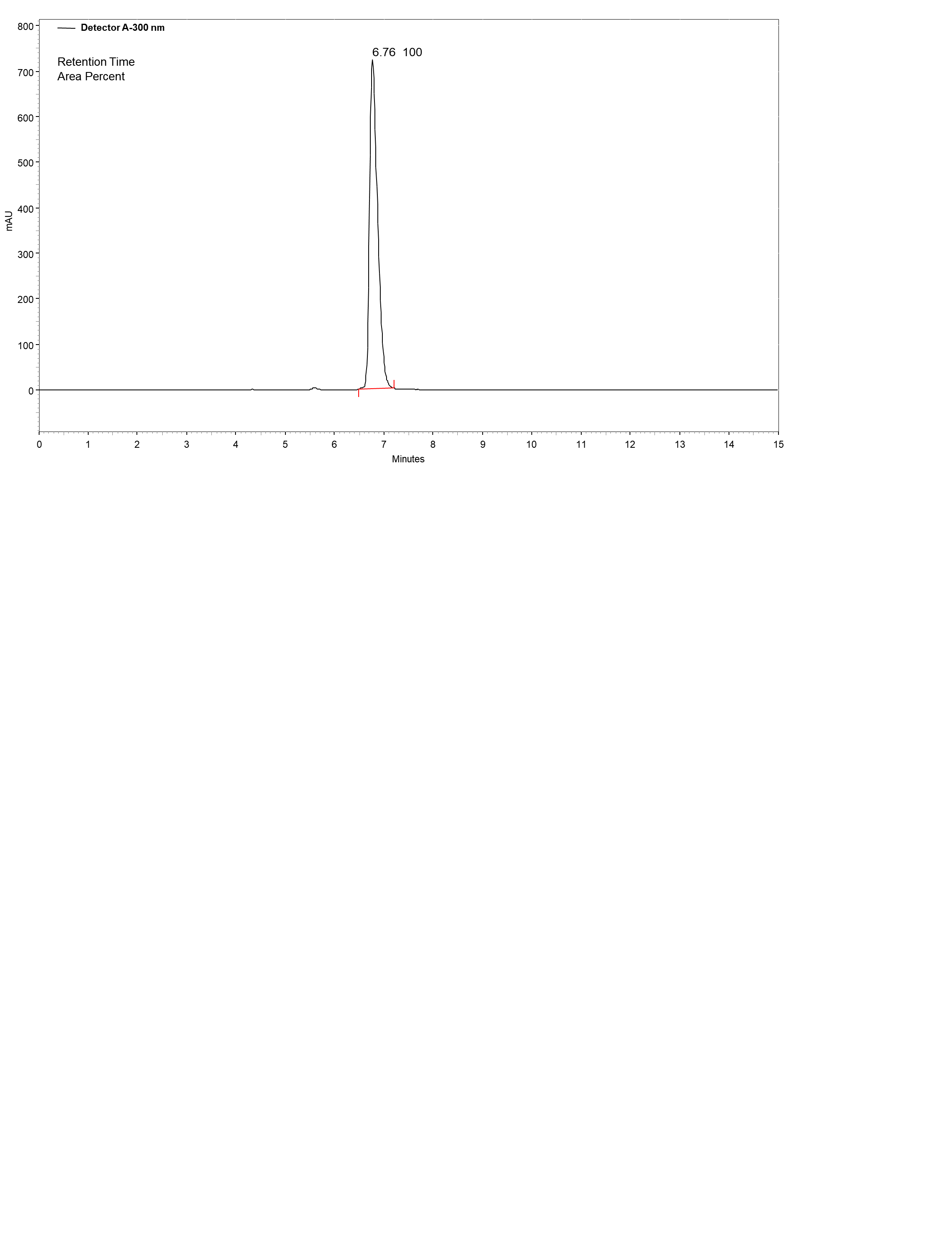


**Figure S76.** HPLC chromatogram of (*E*)-*N*'-((6-((3,5-dimethylphenyl)amino)quinolin-4-yl)methylene)benzohydrazide (**11c**). Mobile phase: ACN 50:50 TFA 0.1%.


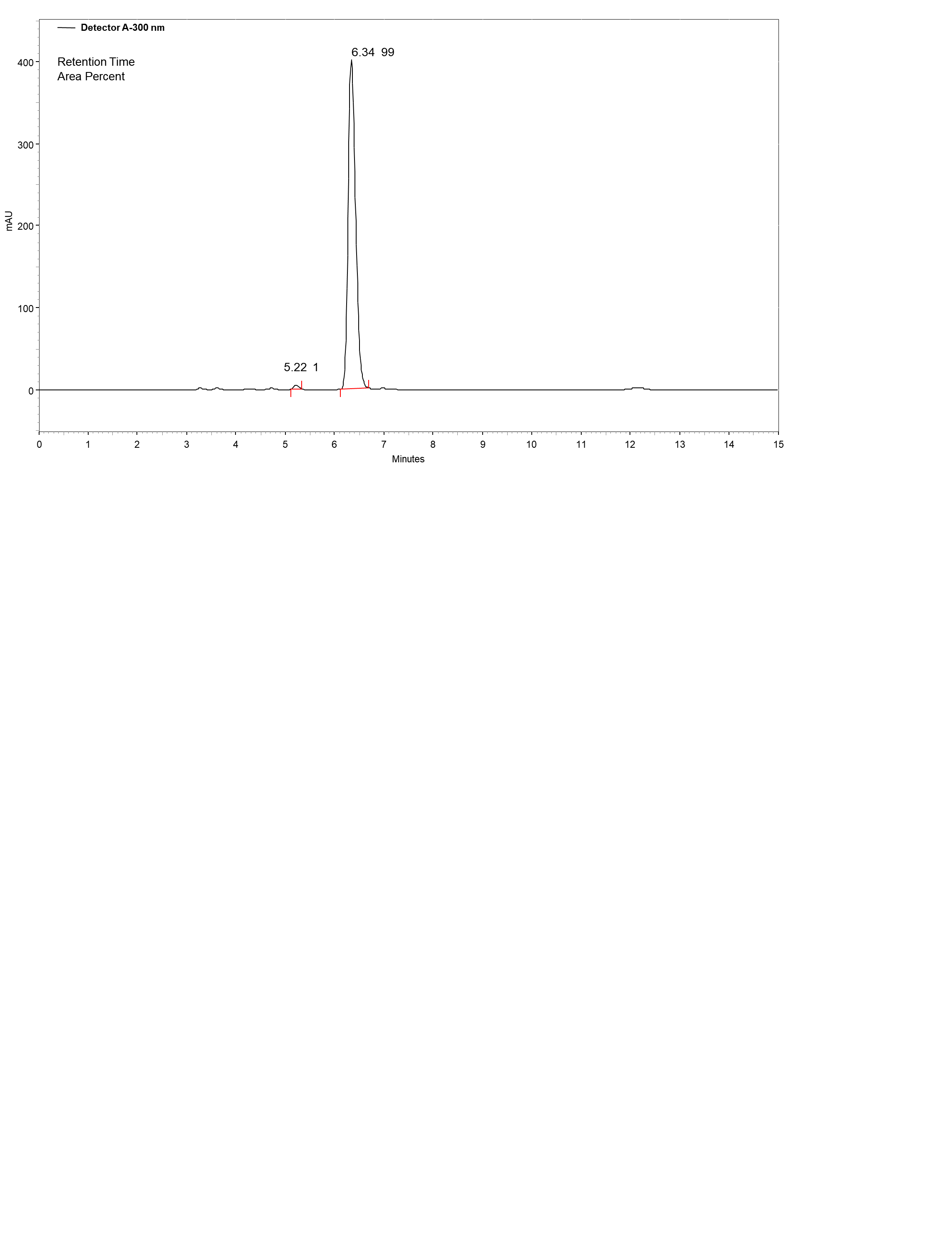


**Figure S77.** HPLC chromatogram of (*E*)-*N*'-((6-((4-chlorophenyl)amino)quinolin-4-yl)methylene)benzohydrazide (**11d**). Mobile phase: ACN 50:50 TFA 0.1%.


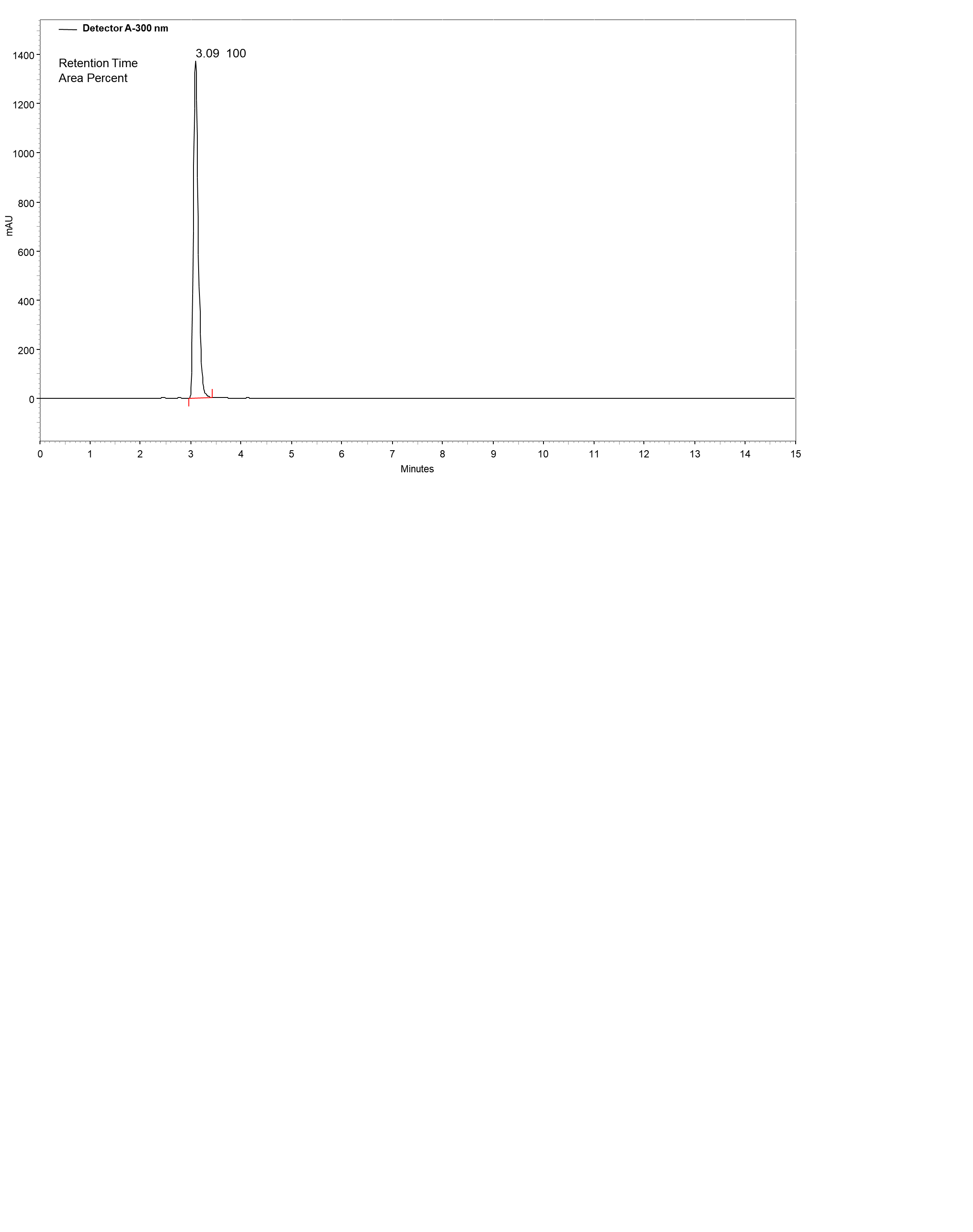


**Figure S78.** HPLC chromatogram of *(E)*-*N*'-((6-(pyrimidin-2-ylamino)quinolin-4-yl)methylene)benzohydrazide (**11e**). Mobile phase: ACN 50:50 TFA 0.1%.


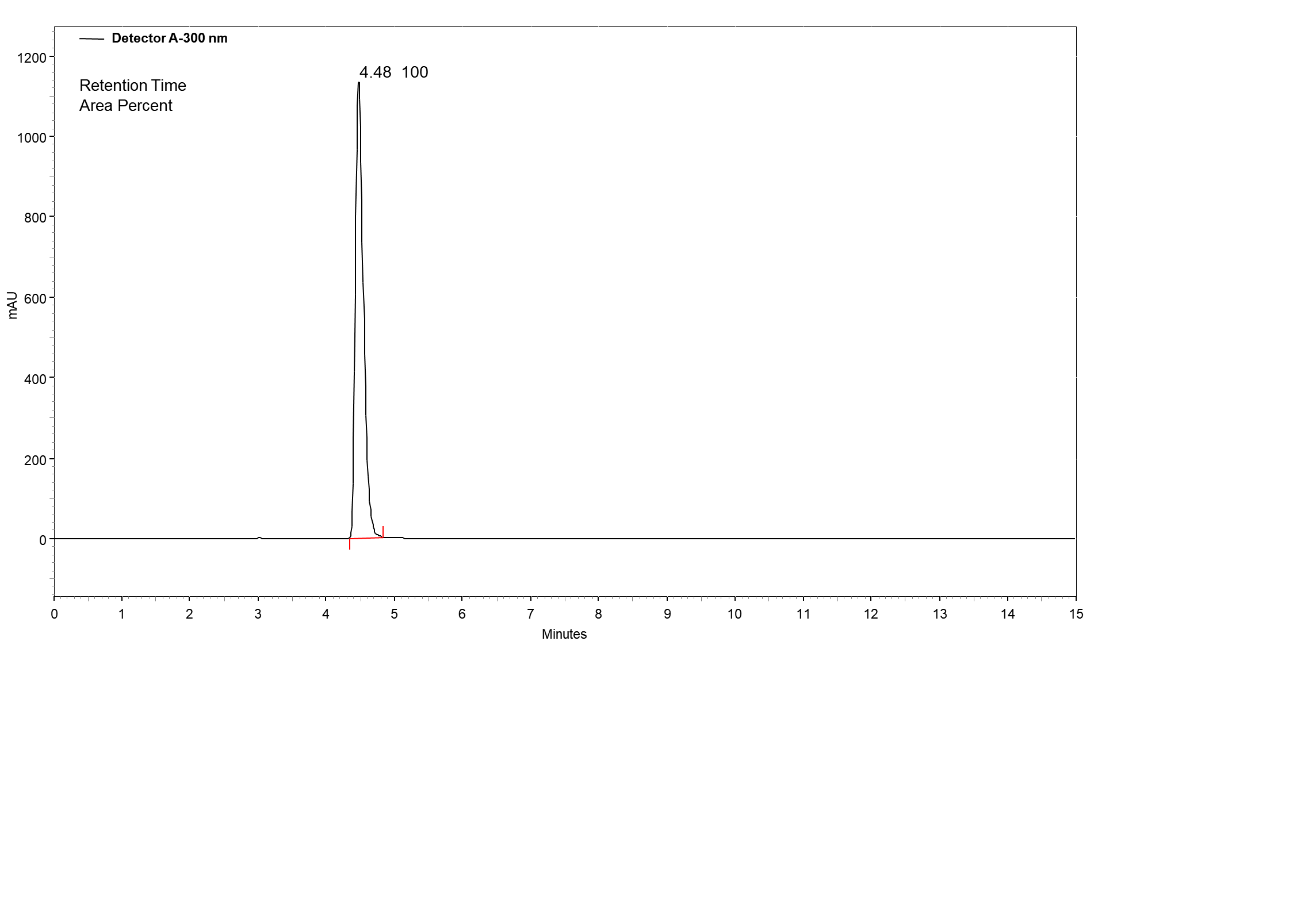


**Figure S79.** HPLC chromatogram of (*E*)-*N*'-((6-((4,6-dimethoxypyrimidin-2-yl)amino)quinolin-4-yl)methylene)benzohydrazide (**11f**). Mobile phase: ACN 50:50 TFA 0.1%.


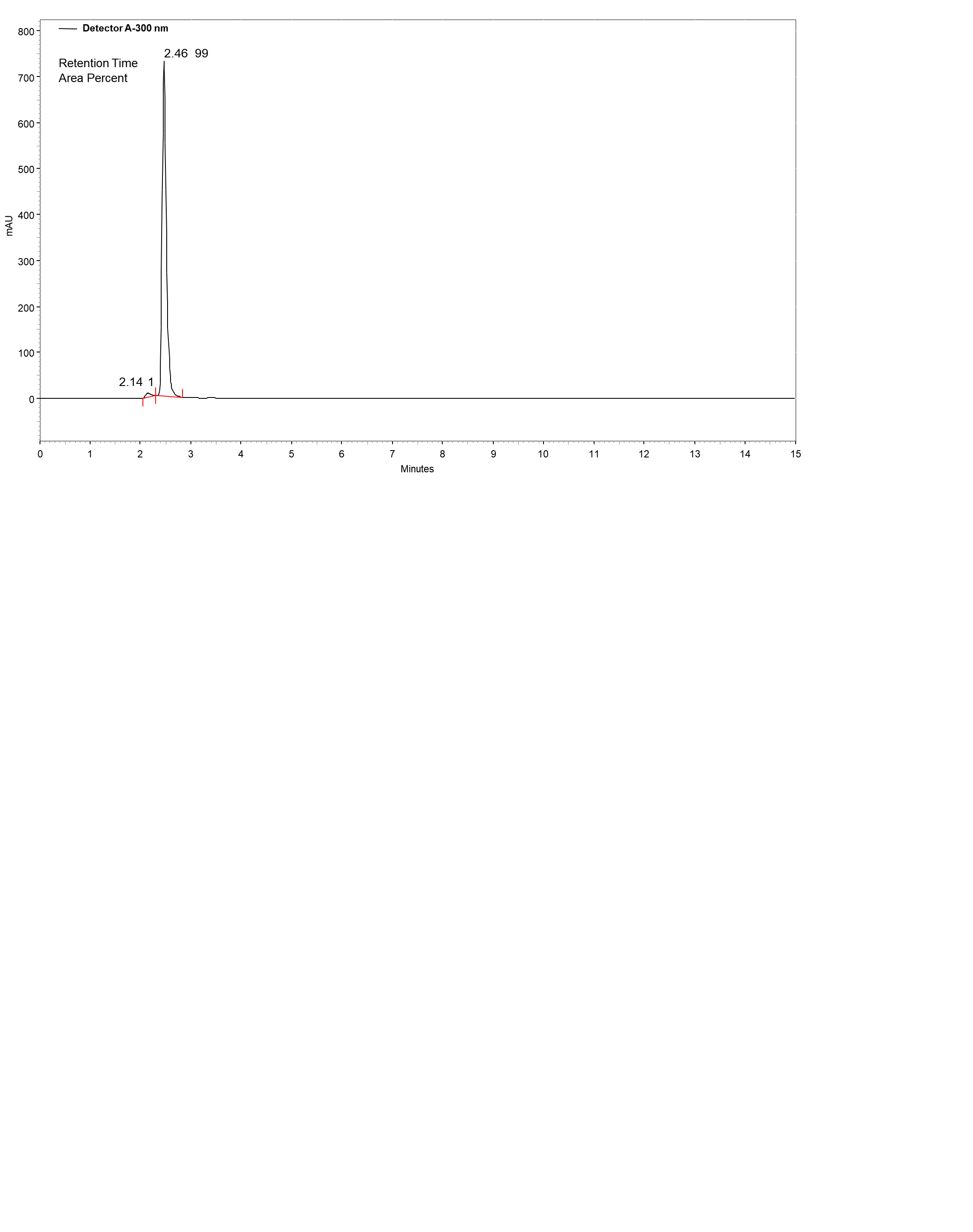


**Figure S80.** HPLC chromatogram of *(E)*-2-((6-(pyridin-2-ylamino)quinolin-4-yl)methylene)hydrazine-1-carbothioamide (**3a**). Mobile phase: ACN 50:50 TFA 0.1%.


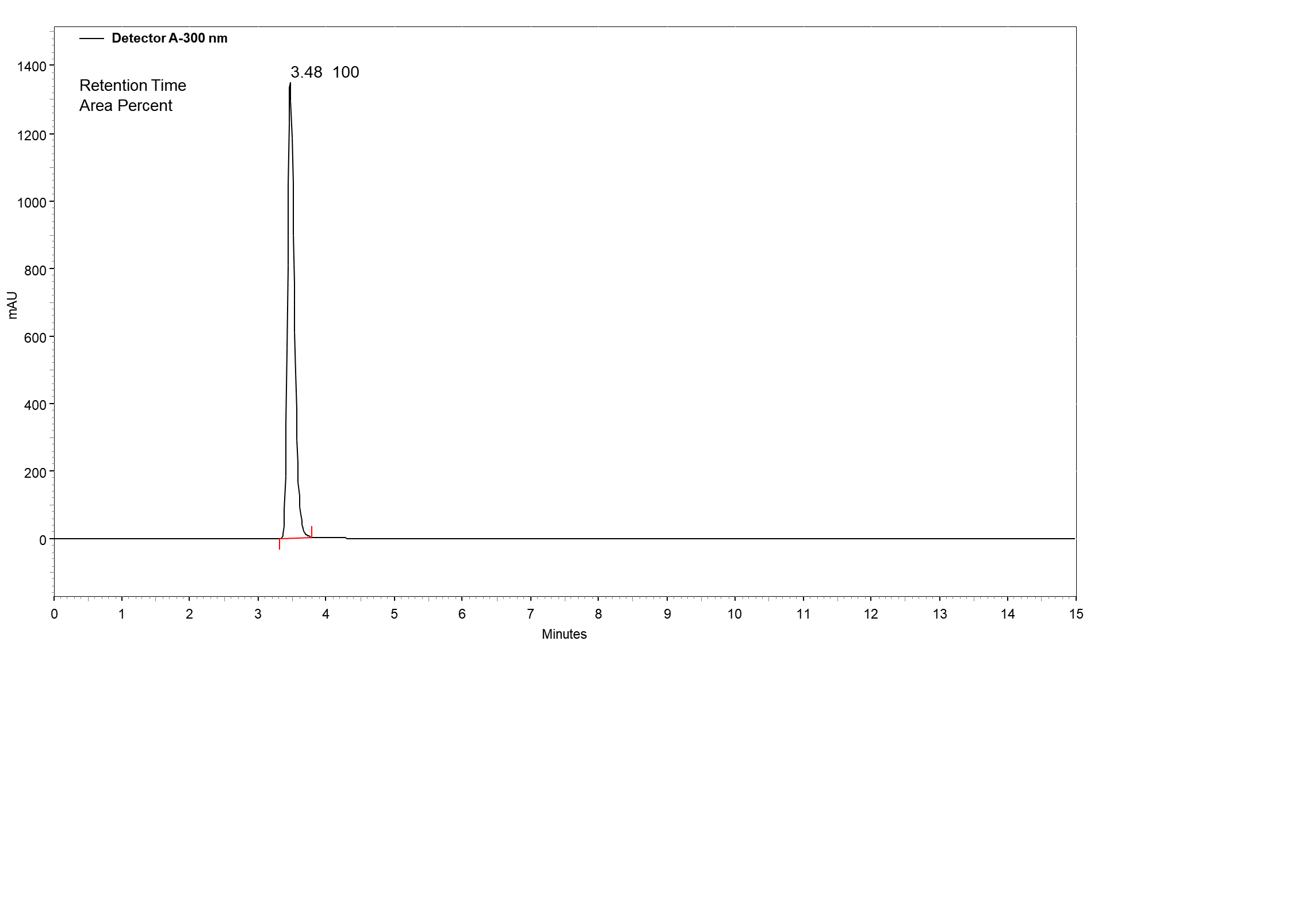


**Figure S81.** HPLC chromatogram of *(E)*-2-((6-((4-methoxyphenyl)amino)quinolin-4-yl)methylene)hydrazinecarbothioamide (**3b**). Mobile phase: ACN 50:50 TFA 0.1%.


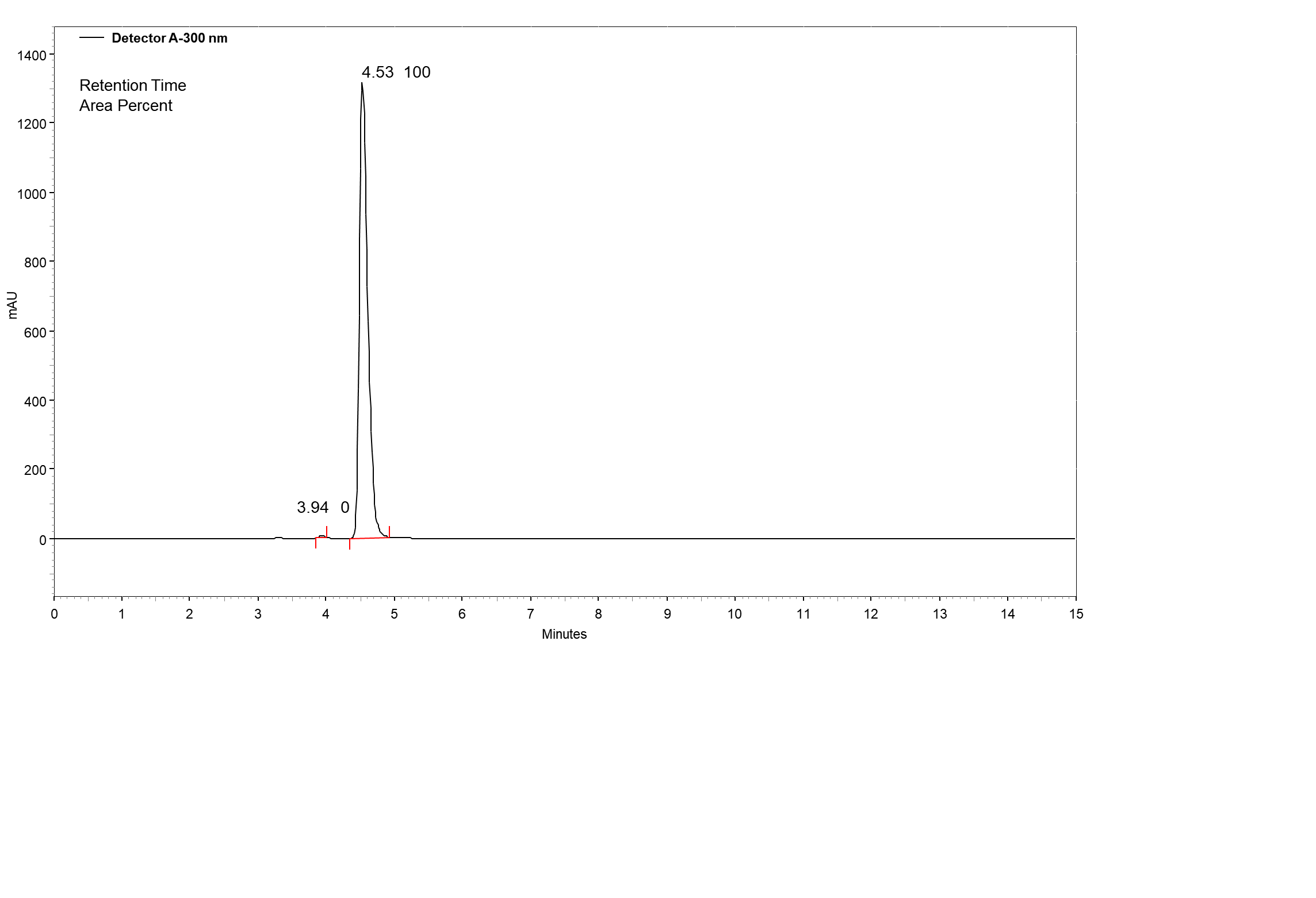


**Figure S82.** HPLC chromatogram of *(E)*-2-((6-((3,5-dimethylphenyl)amino)quinolin-4-yl)methylene)hydrazine-1-carbothioamide (**3c**). Mobile phase: ACN 50:50 TFA 0.1%.


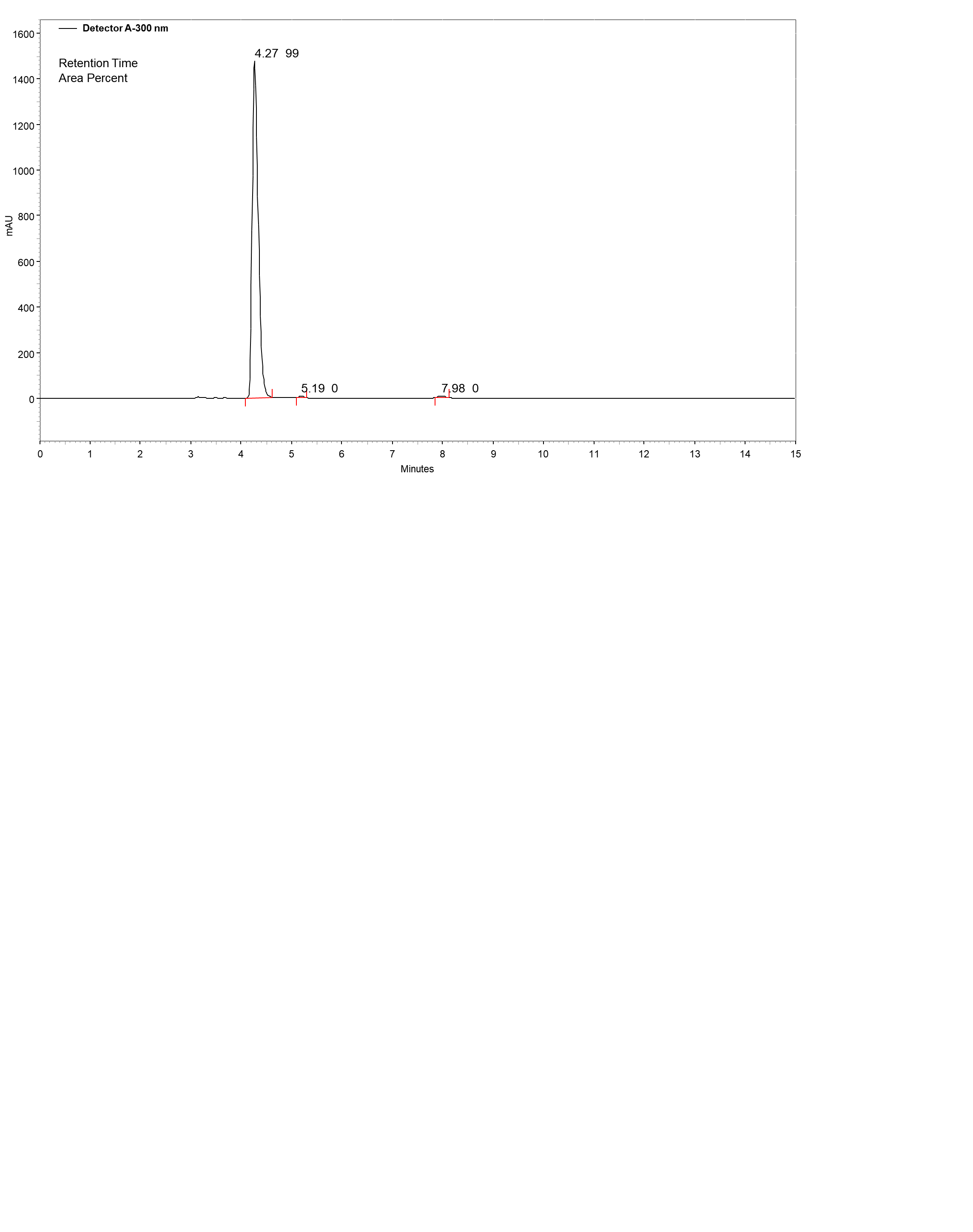


**Figure S83.** HPLC chromatogram of *(E)*-2-((6-((4-chlorophenyl)amino)quinolin-4-yl)methylene)hydrazine-1-carbothioamide (**3d**). Mobile phase: ACN 50:50 TFA 0.1%.


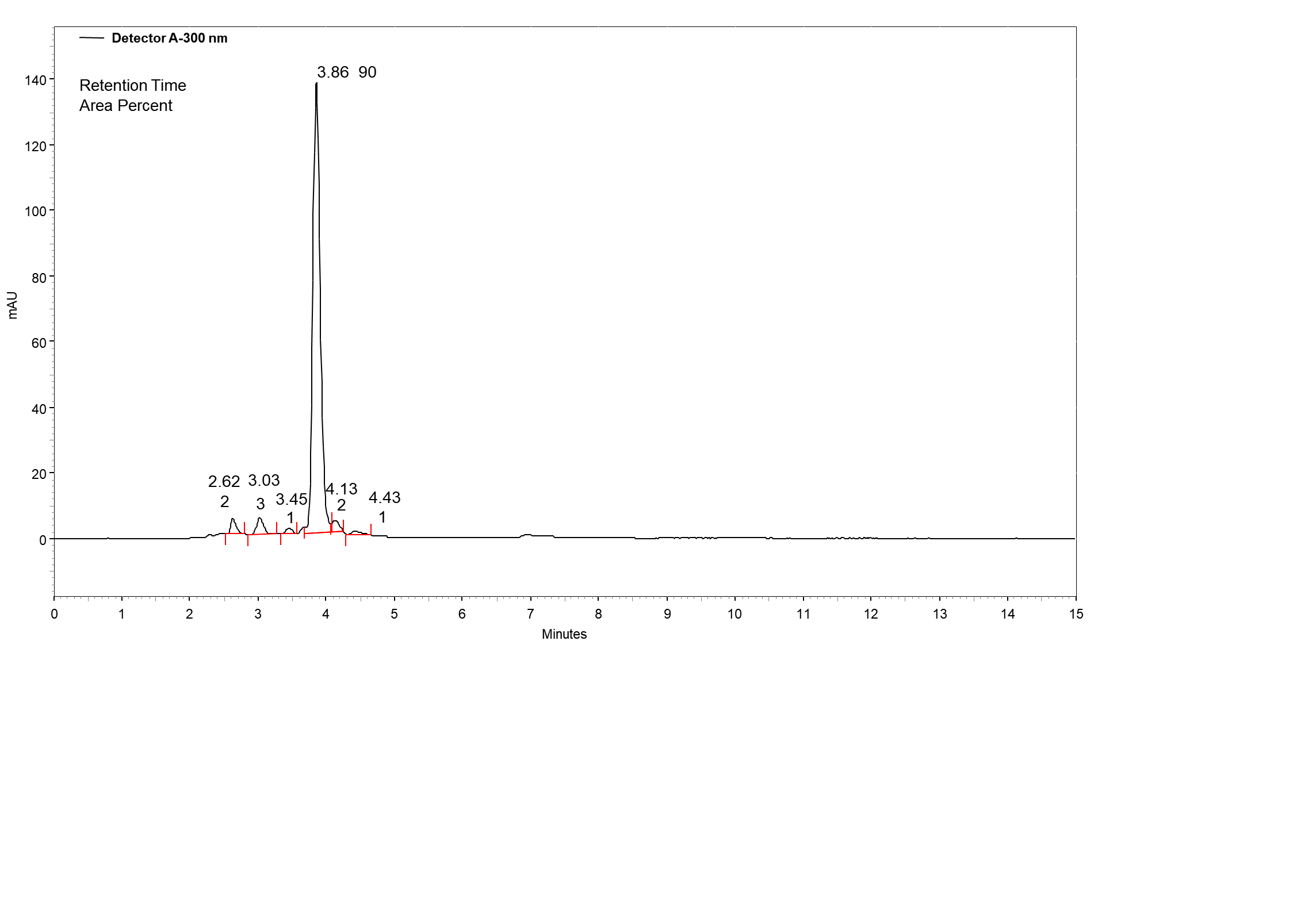


**Figure S84.** HPLC chromatogram of (*E*)-2-((6-((2-chloro-5-((4-methylphenyl)sulfonamido)pyridin-3-yl)amino)quinolin-4-yl)methylene)hydrazine-1-carbothioamide (**3e**). Mobile phase: ACN 50:50 TFA 0.1%.


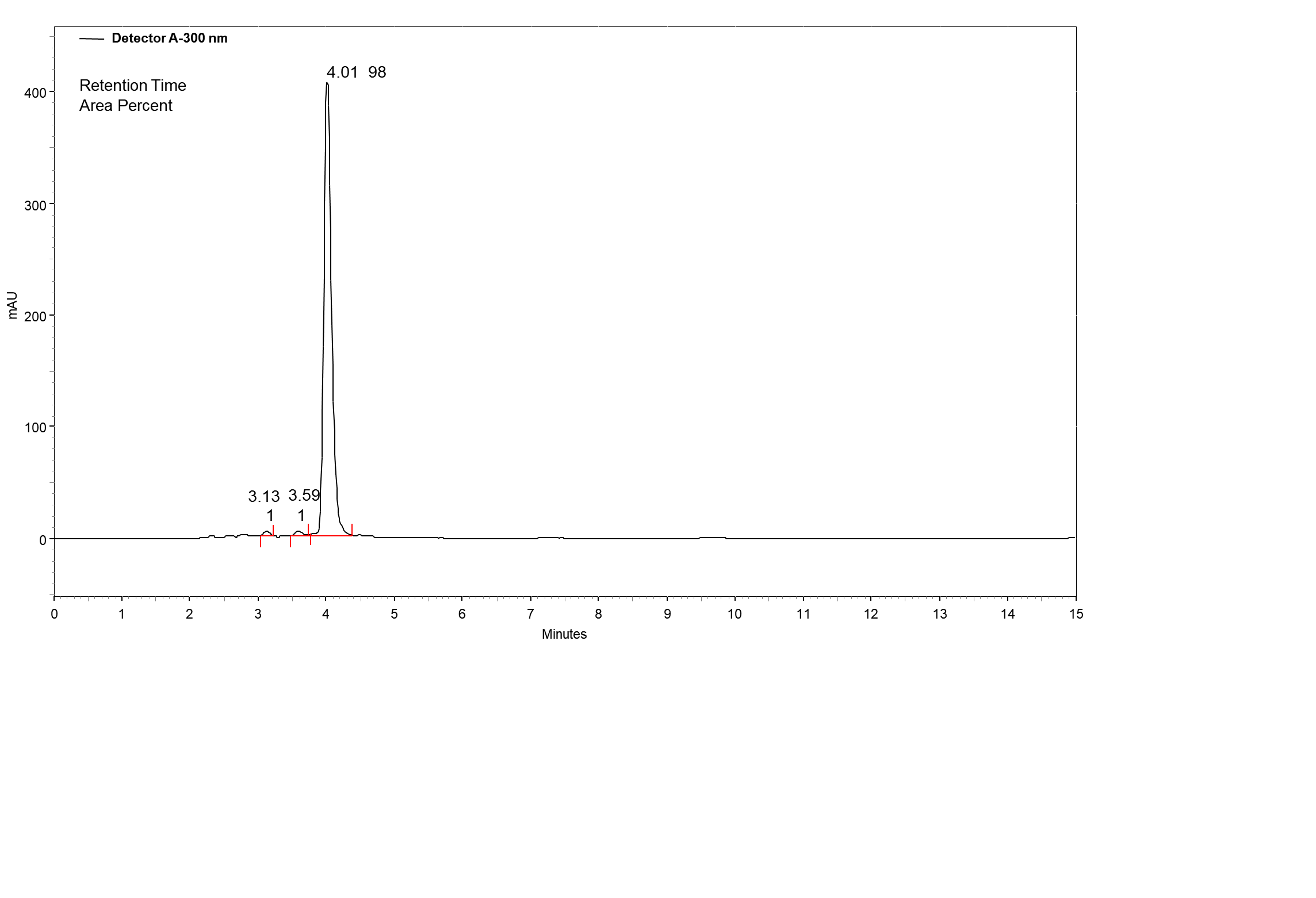


**Figure S85.** HPLC chromatogram of (*E*)-2-((6-((6-((4-methylphenyl)sulfonamido)pyridin-2-yl)amino)quinolin-4-yl)methylene)hydrazine-1-carbothioamide (**3f**). Mobile phase: ACN 50:50 TFA 0.1%.


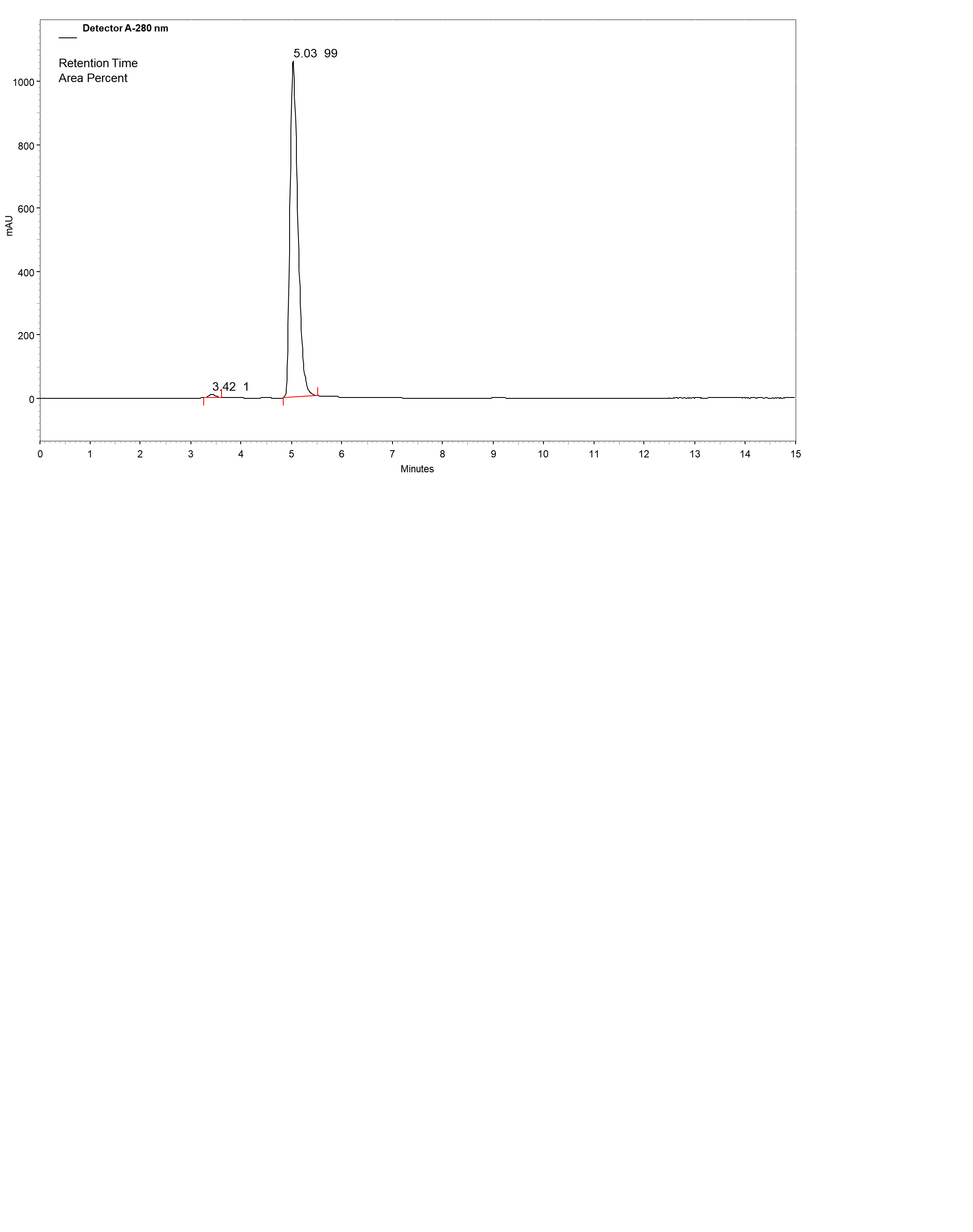


**Figure S86.** HPLC chromatogram of (*E*)-4-{[2-(4-Phenylthiazol-2-yl)hydrazono]methyl}-N-(pyridin-2-yl)quinolin-6-amine (**12a**). Mobile phase: MeOH 80:20 TFA 0.1%.


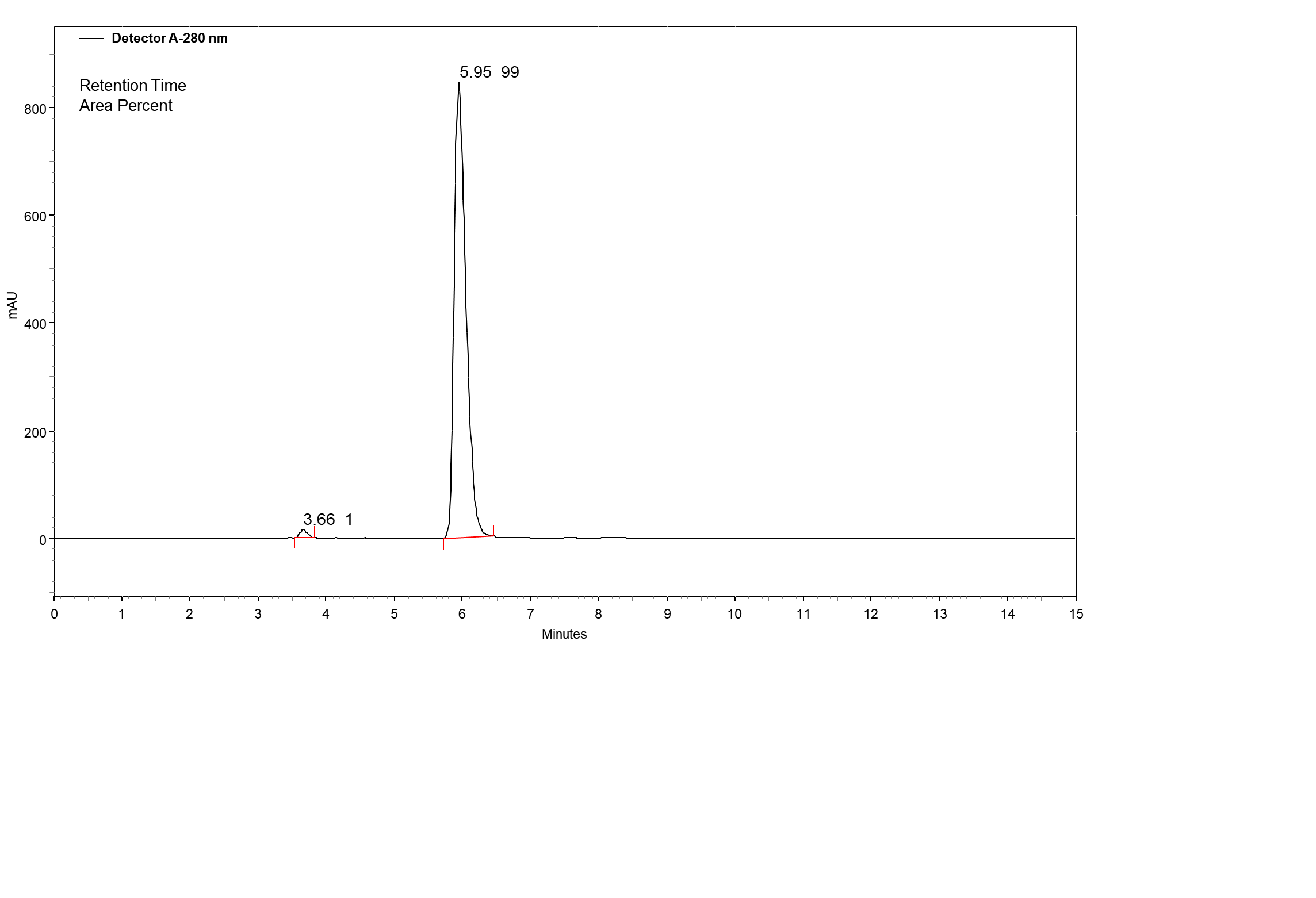


**Figure S87.** HPLC chromatogram of (*E*)-*N*-(4-methoxyphenyl)-4-((2-(4-phenylthiazol-2-yl)hydrazineylidene)methyl)quinolin-6-amine (**12b**). Mobile phase: MeOH 80:20 TFA 0.1%.


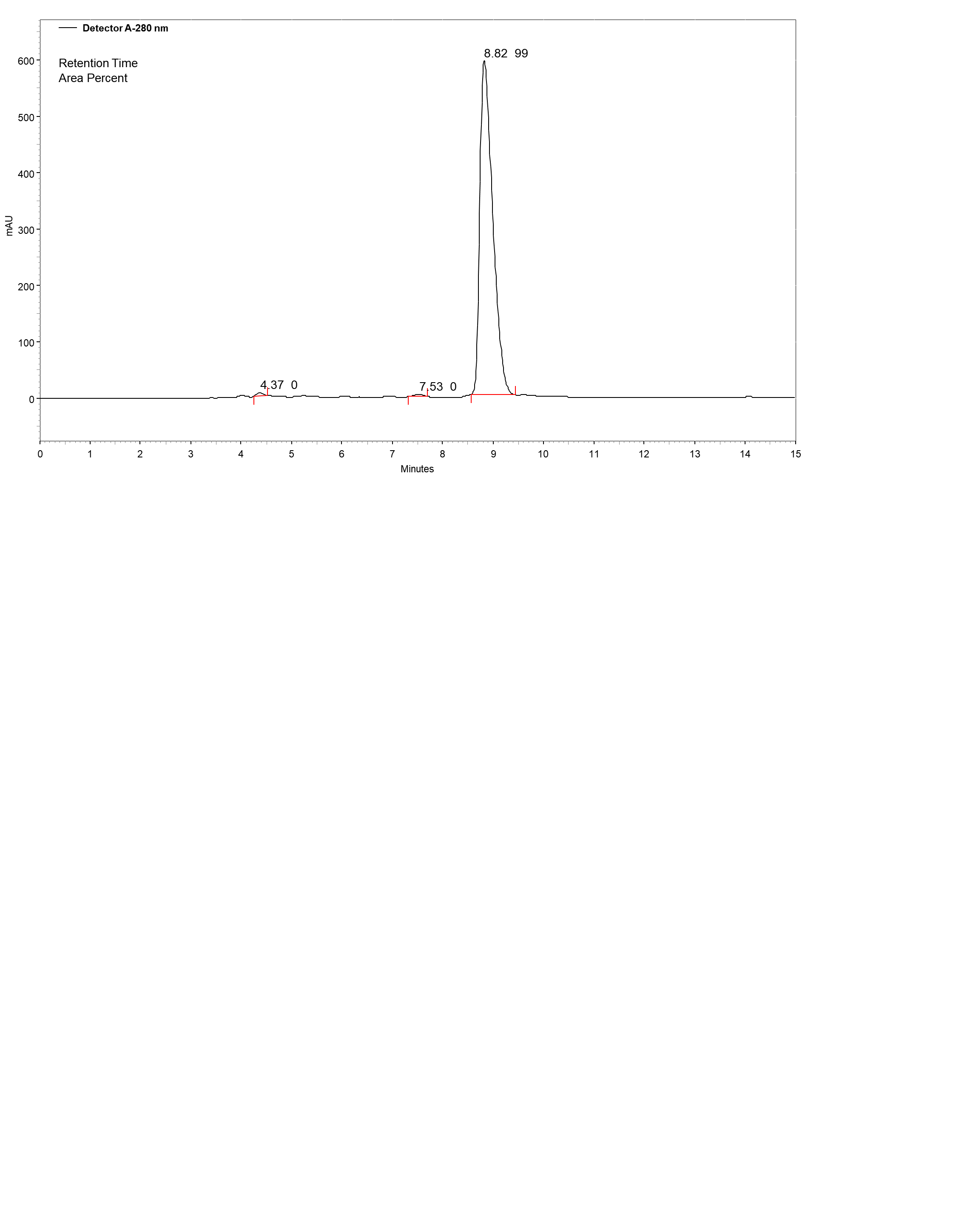


**Figure S88.** HPLC chromatogram of (*E*)-*N*-(3,5-dimethylphenyl)-4-((2-(4-phenylthiazol-2-yl)hydrazono)methyl)quinolin-6-amine (**12c**). Mobile phase: MeOH 80:20 TFA 0.1%.


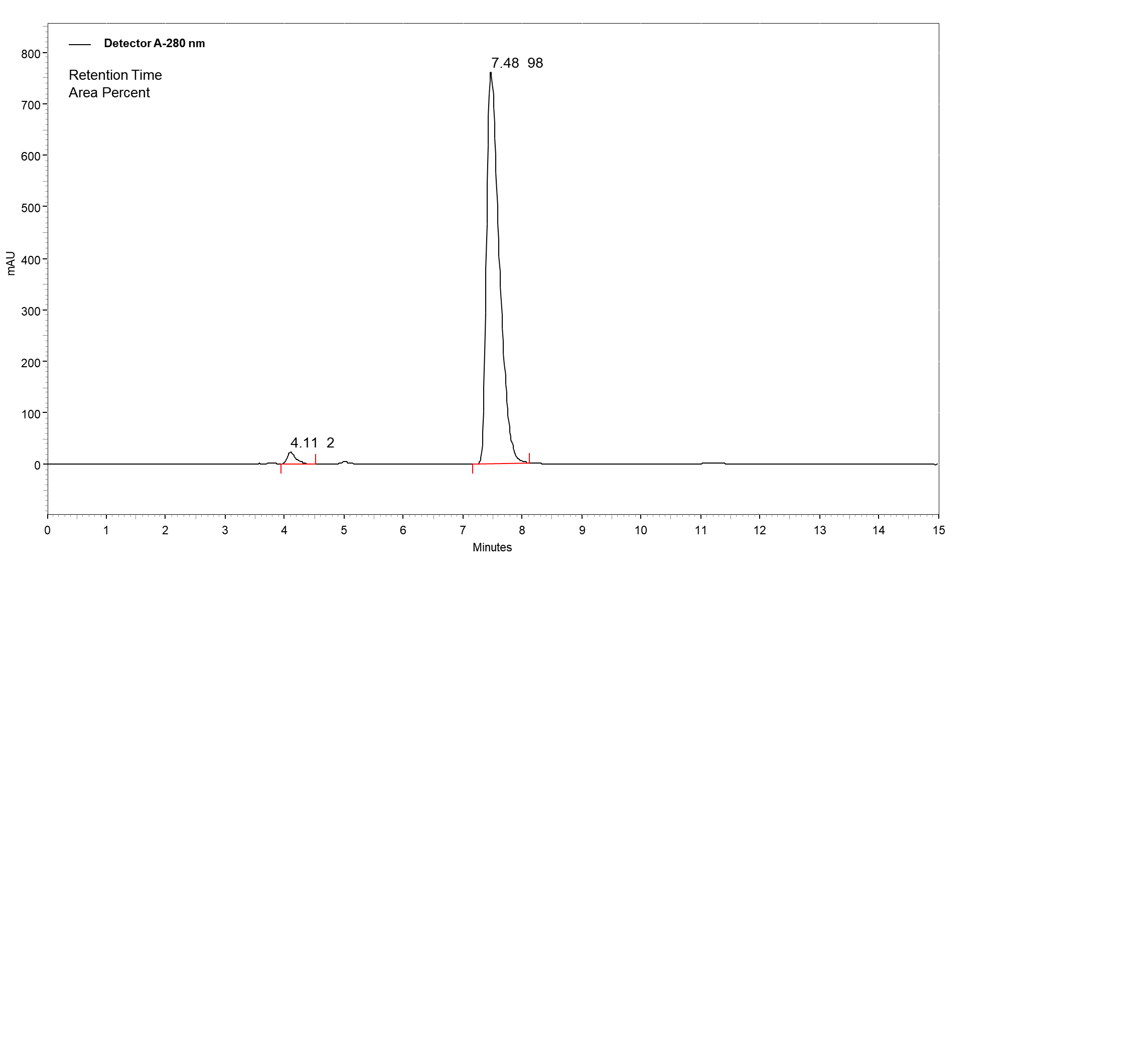


**Figure S89.** HPLC chromatogram of (*E*)-*N*-(4-chlorophenyl)-4-((2-(4-phenylthiazol-2-yl)hydrazineylidene)methyl)quinolin-6-amine (**12d**). Mobile phase: MeOH 80:20 TFA 0.1%.


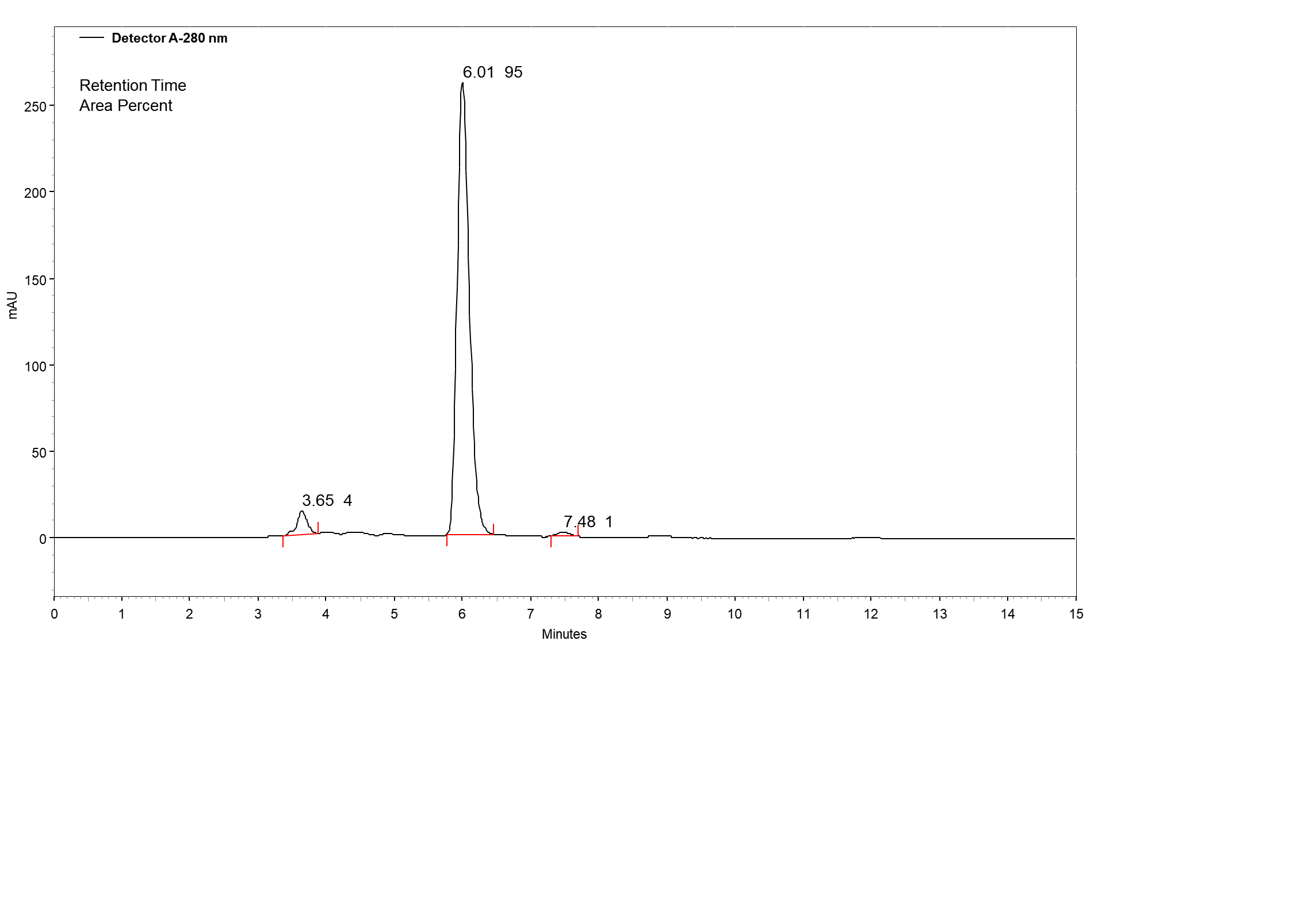


**Figure S90.** HPLC chromatogram of (*E*)-4-methyl-*N*-(6-((4-((2-(4-phenylthiazol-2-yl)hydrazineylidene)methyl)quinolin-6-yl)amino)pyridin-2-yl)benzenesulfonamide (**12e**). Mobile phase: MeOH 80:20 TFA 0.1%.


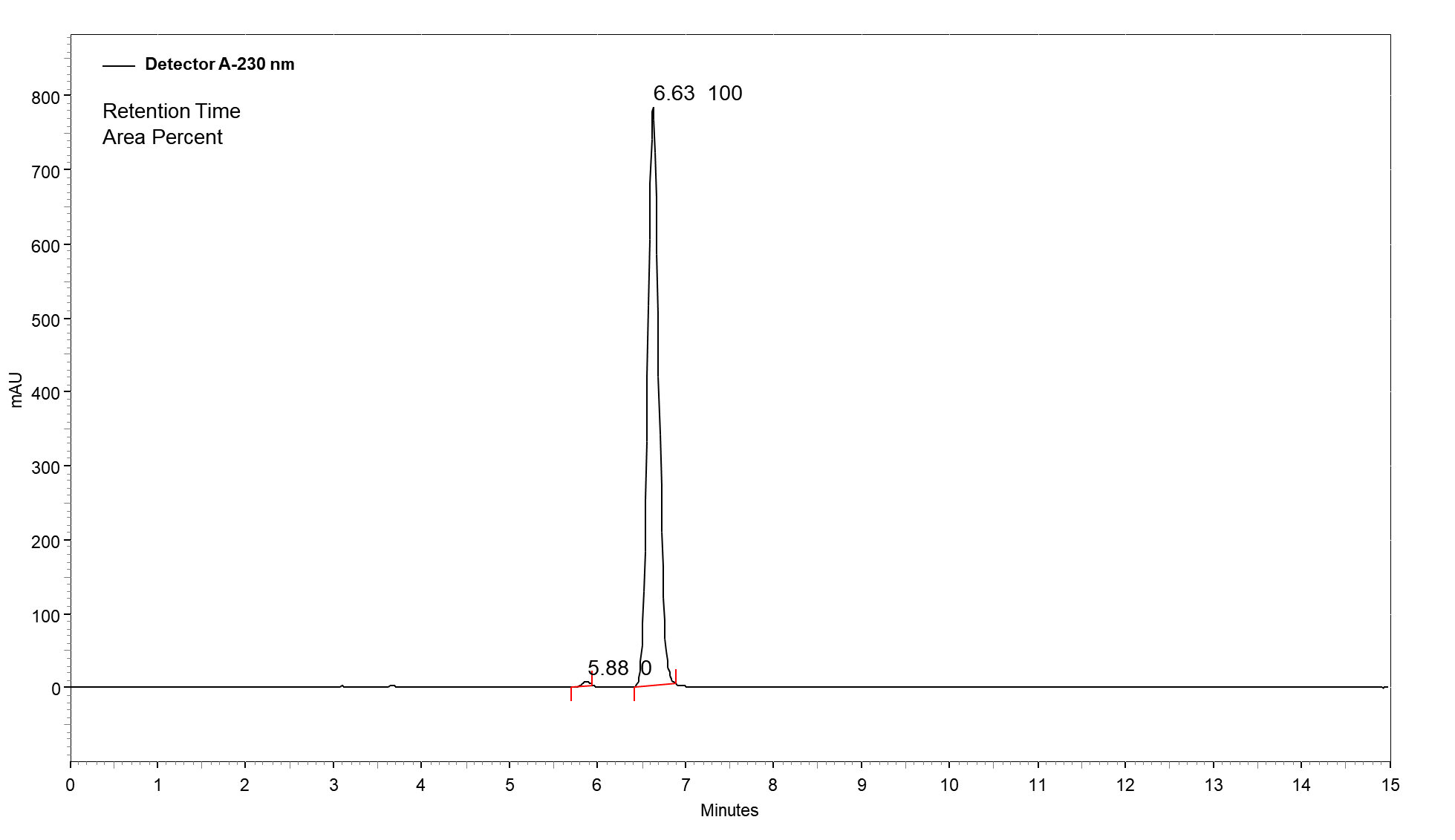


**Figure S91.** HPLC chromatogram of *N*-(5-amino-6-chloropyridin-3-yl)-4-methylbenzenesulfonamide (**16**). Mobile phase: ACN 50:50 TFA 0.1%.


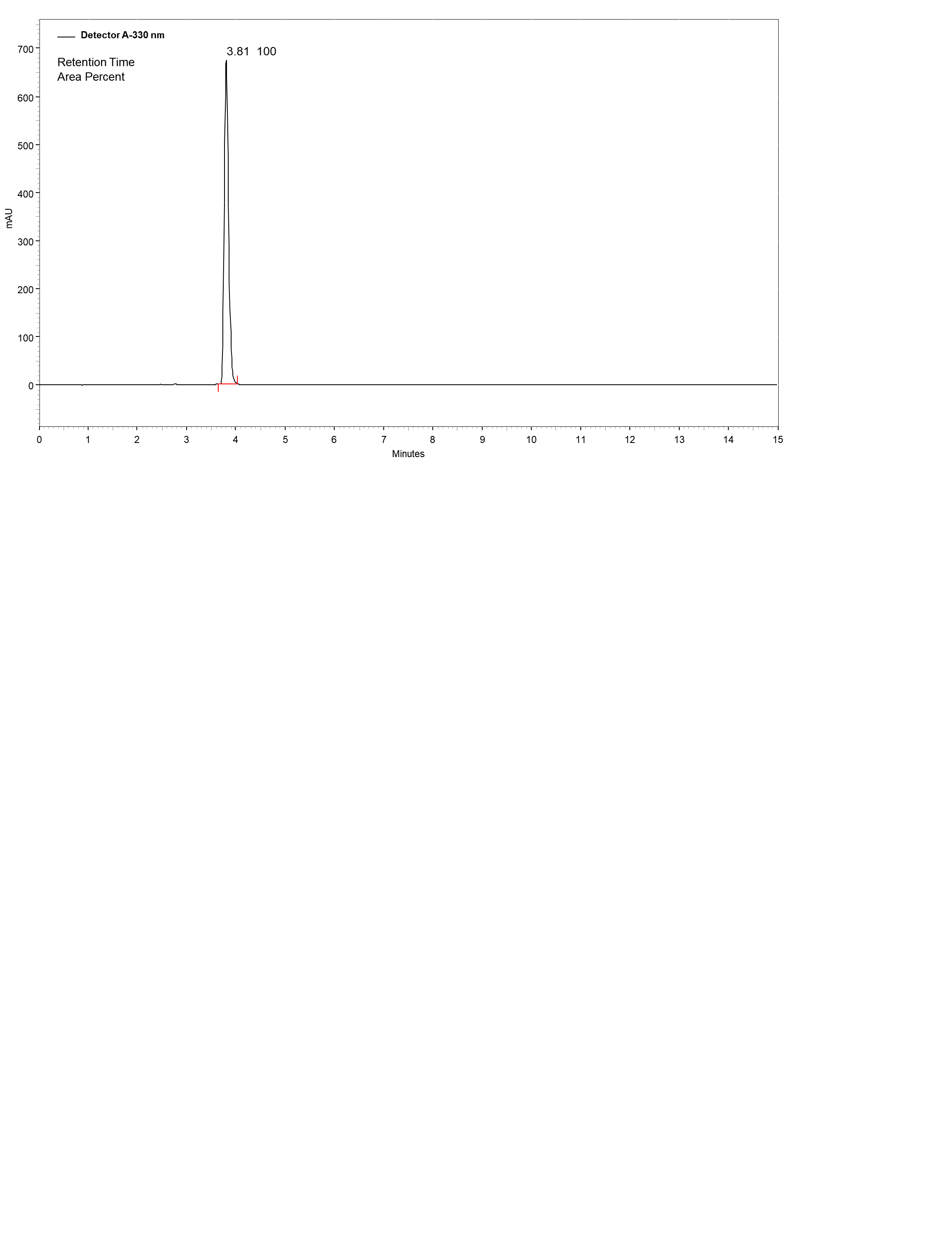


**Figure S92.** HPLC chromatogram of *N*-(6-aminopyridin-2-yl)-4-methylbenzenesulfonamide (**18**). Mobile phase: ACN 50:50 TFA 0.1%.

**REFERENCES**

[1] BIOVIA, Dassault Systèmes, Discovery Studio Visualizer, v25.1.0, **2025**, San Diego: Dassault Systèmes, 2024.
