# Supplementary material for: New Quinoline Kinase Inhibitors With Good Selectivity for NAK Kinases and Anti‐Tumor Activity Against Ewing Sarcoma
Source: Arch Pharm (Weinheim). 2026 Jan 10;359(1):e70184. doi: 10.1002/ardp.70184 (PMC12790329; doi:10.1002/ardp.70184)
Supplement: Supplementary file 2 — Supporting_Publishing_InChI. [file ARDP-359-e70184-s001.docx]

**Supplemental Material: Novel Compounds and Biological Screening Results**

New quinoline kinase inhibitors with good selectivity for NAK kinases and anti-tumor activity against Ewing Sarcoma

Caroline de Bem Gentz^1,2^, Thais Helena Maciel Fernandes^1,2^, Marcela Silva Lopes^3,4^, Lewis Elson^5^, Andreas Krämer^5^, Lucas Rodrigo de Souza^6^, Isadora Serraglio Fortes^1,2,4^, Geórgia Silva Pinto^2^, Martha Cestari Silva Martins^1,2^, Henrique Barros de Lima^1,2^, André da Silva Santiago^6^, Lauro José Gregianin^4,7,8,9^, Katlin Brauer Massirer^6^, Mário Henrique Bengtson^6^, Rafael Roesler^3,4,7^, Stefan Knapp^5^, Stefan A. Laufer^10*^, Saulo Fernandes de Andrade^1,2,4*^

1 Pharmaceutical Sciences Graduate Program, Universidade Federal do Rio Grande do Sul (UFRGS), Porto Alegre, RS, Brazil;

2 Pharmaceutical Synthesis Group (PHARSG), School of Pharmacy, Universidade Federal do Rio Grande do Sul (UFRGS), Porto Alegre, RS, Brazil;

3 Department of Pharmacology, Institute of Basic Health Sciences, Universidade Federal do Rio Grande do Sul (UFRGS), Porto Alegre, RS, Brazil;

4 National Science and Technology Institute for Children’s Cancer Biology and Pediatric Oncology - INCT BioOncoPed, Porto Alegre, Brazil;

5 Institute of Pharmaceutical Chemistry, Goethe University, Frankfurt am Main, Germany;

6 Center for Medicinal Chemistry (CQMED), Center for Molecular Biology and Genetic Engineering (CBMEG), University of Campinas (UNICAMP), Campinas, SP, Brazil;

7 Cancer and Neurobiology Laboratory, Experimental Research Center, Clinical Hospital (CPE-HCPA), Universidade Federal do Rio Grande do Sul, Porto Alegre, RS, Brazil;

8 Department of Pediatrics, School of Medicine, Universidade Federal do Rio Grande do Sul, Porto Alegre, RS, Brazil;

9 Pediatric Oncology Service, Clinical Hospital, Universidade Federal do Rio Grande do Sul, Porto Alegre, RS, Brazil;

10 Department of Pharmaceutical and Medicinal Chemistry, Institute of Pharmaceutical Sciences, University of Tübingen, Tübingen, Germany.

*Correspondence:

Prof, Saulo Fernandes de Andrade, Pharmaceutical Sciences Graduate Program, Universidade Federal do Rio Grande do Sul, Porto Alegre, Rio Grande do Sul, Brazil. 90010-150. Pharmaceutical Synthesis Group (PHARSG), School of Pharmacy, Universidade Federal do Rio Grande do Sul (UFRGS), Porto Alegre, RS, Brazil; National Science and Technology Institute for Children’s Cancer Biology and Pediatric Oncology - INCT BioOncoPed, Porto Alegre, Brazil.

Email: saulo.fernandes@ufrgs.br

Prof, Stefan A. Laufer - Department of Pharmaceutical and Medicinal Chemistry, Institute of Pharmaceutical Sciences, University of Tübingen, Tübingen, 72074, Germany.

Email: stefan.laufer@uni-tuebingen.de

| **Compound No.** | **InChI** | **% inhibition RD-ES (10 μM)^a^** | **% inhibition GAK (1 μM)^b^** |
| --- | --- | --- | --- |
| **12a** | InChI=1S/C24H18N6S/c1-2-6-17(7-3-1)22-16-31-24(29-22)30-27-15-18-11-13-25-21-10-9-19(14-20(18)21)28-23-8-4-5-12-26-23/h1-16H,(H,26,28)(H,29,30)/b27-15+ | 25.1 ± 0.0% | 4.9% ± 8.2% |
| **12b** | InChI=1S/C26H21N5OS/c1-32-22-10-7-20(8-11-22)29-21-9-12-24-23(15-21)19(13-14-27-24)16-28-31-26-30-25(17-33-26)18-5-3-2-4-6-18/h2-17,29H,1H3,(H,30,31)/b28-16+ | 21.1 ± 0.2% | 12.1% ± 10.2% |
| **12c** | InChI=1S/C27H23N5S/c1-18-12-19(2)14-23(13-18)30-22-8-9-25-24(15-22)21(10-11-28-25)16-29-32-27-31-26(17-33-27)20-6-4-3-5-7-20/h3-17,30H,1-2H3,(H,31,32)/b29-16+ | 33.2 ± 0.1% | 13.4% ± 11.4% |
| **12d** | InChI=1S/C25H18ClN5S/c26-19-6-8-20(9-7-19)29-21-10-11-23-22(14-21)18(12-13-27-23)15-28-31-25-30-24(16-32-25)17-4-2-1-3-5-17/h1-16,29H,(H,30,31)/b28-15+ | 49.6 ± 0.2% | 23.3% ± 5.4% |
| **11a** | InChI=1S/C22H17N5O/c28-22(16-6-2-1-3-7-16)27-25-15-17-11-13-23-20-10-9-18(14-19(17)20)26-21-8-4-5-12-24-21/h1-15H,(H,24,26)(H,27,28)/b25-15+ | 52.8 ± 0.1% | 62.8% ± 9.4% |
| **11b** | InChI=1S/C24H20N4O2/c1-30-21-10-7-19(8-11-21)27-20-9-12-23-22(15-20)18(13-14-25-23)16-26-28-24(29)17-5-3-2-4-6-17/h2-16,27H,1H3,(H,28,29)/b26-16+ | 57.9 ± 0.2% | 40.1% ± 2% |
| **11c** | InChI=1S/C25H22N4O/c1-17-12-18(2)14-22(13-17)28-21-8-9-24-23(15-21)20(10-11-26-24)16-27-29-25(30)19-6-4-3-5-7-19/h3-16,28H,1-2H3,(H,29,30)/b27-16+ | 38.4 ± 0.1% | 33.6% ± 5.7% |
| **11d** | InChI=1S/C23H17ClN4O/c24-18-6-8-19(9-7-18)27-20-10-11-22-21(14-20)17(12-13-25-22)15-26-28-23(29)16-4-2-1-3-5-16/h1-15,27H,(H,28,29)/b26-15+ | 49.6 ± 0.1% | 27.1% ± 6.6% |
| **11e** | InChI=1S/C21H16N6O/c28-20(15-5-2-1-3-6-15)27-25-14-16-9-12-22-19-8-7-17(13-18(16)19)26-21-23-10-4-11-24-21/h1-14H,(H,27,28)(H,23,24,26)/b25-14+ | 56.6 ± 0.0% | 66.1% ± 7.4% |
| **11f** | InChI=1S/C23H20N6O3/c1-31-20-13-21(32-2)28-23(27-20)26-17-8-9-19-18(12-17)16(10-11-24-19)14-25-29-22(30)15-6-4-3-5-7-15/h3-14H,1-2H3,(H,29,30)(H,26,27,28)/b25-14+ | 57.7 ± 0.0% | 37.2% ± 5.5% |
| **3b** | InChI=1S/C18H17N5OS/c1-24-15-5-2-13(3-6-15)22-14-4-7-17-16(10-14)12(8-9-20-17)11-21-23-18(19)25/h2-11,22H,1H3,(H3,19,23,25)/b21-11+ | 48.5 ± 0.0% | 77.3% ± 5.5% |
| **3c** | InChI=1S/C19H19N5S/c1-12-7-13(2)9-16(8-12)23-15-3-4-18-17(10-15)14(5-6-21-18)11-22-24-19(20)25/h3-11,23H,1-2H3,(H3,20,24,25)/b22-11+ | 74.3 ± 0.1% | 88.7% ± 4.5% |
| **3d** | InChI=1S/C17H14ClN5S/c18-12-1-3-13(4-2-12)22-14-5-6-16-15(9-14)11(7-8-20-16)10-21-23-17(19)24/h1-10,22H,(H3,19,23,24)/b21-10+ | 36.9 ± 0.1% | 60.4% ± 9.8% |
| **3e** | InChI=1S/C23H20ClN7O2S2/c1-14-2-5-18(6-3-14)35(32,33)31-17-11-21(22(24)27-13-17)29-16-4-7-20-19(10-16)15(8-9-26-20)12-28-30-23(25)34/h2-13,29,31H,1H3,(H3,25,30,34)/b28-12+ | 6.2 ± 0.1% | 8.8% ± 6.6% |
| **3f** | InChI=1S/C23H21N7O2S2/c1-15-5-8-18(9-6-15)34(31,32)30-22-4-2-3-21(28-22)27-17-7-10-20-19(13-17)16(11-12-25-20)14-26-29-23(24)33/h2-14H,1H3,(H3,24,29,33)(H2,27,28,30)/b26-14- | 40.6 ± 0.0% | 7.9% ± 5.9% |

^a^The compounds were added to the plates containing the RD-ES strain at 10 µM (previously diluted in culture media), and the plates were then incubated at 5% CO2 at 37 °C. Following a 48 hour period, the viable cell concentration was calculated using MTT solution (5 mg/mL in PBS). The optical density was measured in a spectrophotometer at 570 nm.

^b^ The inhibition assay (compounds at 1 µM) was conducted using ADP-Glo™ assay kit with purified GAK and ATP (100 µM =~2 x GAK KmATP).
